# Swimming downstream: statistical analysis of differential transcript usage following Salmon quantification

**DOI:** 10.12688/f1000research.15398.3

**Published:** 2018-10-01

**Authors:** Michael I. Love, Charlotte Soneson, Rob Patro

**Affiliations:** 1 Department of Biostatistics, University of North Carolina at Chapel Hill, Chapel Hill, NC, 27516, USA; 2 Department of Genetics, University of North Carolina at Chapel Hill, Chapel Hill, NC, 27516, USA; 3 Institute of Molecular Life Sciences, University of Zurich, Zurich, Switzerland; 4 SIB Swiss Institute of Bioinformatics, Zurich, Switzerland; 5 Department of Computer Science, Stony Brook University, Stony Brook, NY, 11794, USA

**Keywords:** RNA-seq, workflow, differential transcript usage, Salmon, DRIMSeq, DEXSeq, stageR, tximport

## Abstract

Detection of differential transcript usage (DTU) from RNA-seq data is an important bioinformatic analysis that complements differential gene expression analysis. Here we present a simple workflow using a set of existing R/Bioconductor packages for analysis of DTU. We show how these packages can be used downstream of RNA-seq quantification using the Salmon software package. The entire pipeline is fast, benefiting from inference steps by Salmon to quantify expression at the transcript level. The workflow includes live, runnable code chunks for analysis using DRIMSeq and DEXSeq, as well as for performing two-stage testing of DTU using the stageR package, a statistical framework to screen at the gene level and then confirm which transcripts within the significant genes show evidence of DTU. We evaluate these packages and other related packages on a simulated dataset with parameters estimated from real data.

## Introduction

RNA-seq experiments can be analyzed to detect differences across groups of samples in total gene expression – the total expression produced by all isoforms of a gene – and additionally differences in transcript isoform usage within a gene. If the amount of expression switches among two or more isoforms of a gene, the total gene expression may not change by a detectable amount, but the differential transcript usage (DTU) is nevertheless biologically relevant. DTU is common when comparing expression across cell types: recent analysis of the Genotype-Tissue Expression Project (GTEx) ^1^ dataset demonstrated that half of all expressed genes contained tissue-specific isoforms ^2^. DTU may produce functionally different gene products through alternative splicing and changes to the coding sequence of the transcript, and may also result in transcripts with different untranslated regions on the 5’ or 3’ end of the transcript, which can affect binding sites of RNA binding proteins. Reyes and Huber ^2^ found that alternative usage of transcription start and termination sites was a more common event than alternative splicing when examining DTU events across tissues in GTEx. Specific patterns of DTU have been identified in a number of diseases, including cancer, retinal diseases, and neurological disorders, among others ^3^. Large-scale analyses of cancer transcriptomic data, comparing tumor to normal samples, have identified that protein domain losses are a common feature of DTU in cancer, including domains involved in protein-protein interactions ^4, 5^.

While many tutorials and workflows in the Bioconductor project address differential gene expression, there are fewer workflows for performing a differential transcript usage analysis, which provides critical and complementary information to a gene-level analysis. Some of the existing Bioconductor packages and functions that can be used for statistical analysis of DTU include *DEXSeq* (originally designed for differential exon usage) ^6^, diffSpliceDGE from the *edgeR* package ^7, 8^, diffSplice from the *limma* package ^9, 10^, and *DRIMSeq* ^11^. Other Bioconductor packages which are designed around a DTU analysis include *stageR* ^12^, *SGSeq* ^13^, and *IsoformSwitchAnalyzeR* ^14^. *stageR* can be used for post-processing of transcript- and gene-level p-values from DTU detection methods, and will be discussed in this workflow. *SGSeq* can be used to visualize splice events, and leverages *DEXSeq* or *limma* for differential testing of splice variant usage. The Bioconductor package *IsoformSwitchAnalyzeR* is well documented and the vignette available from the IsoformSwitchAnalyzeR landing page can be seen as an alternative to this workflow. *IsoformSwitchAnalyzeR* is designed for the downstream analysis of functional consequences of identified isoform switches. It allows for import of data from various quantification methods, including *Salmon*, and allows for statistical inference using *DRIMSeq*. In addition, *IsoformSwitchAnalyzeR* includes functions for obtaining the nucleotide and amino acid sequence consequences of isoform switching, which is not covered in this workflow. Other packages related to splicing can be found at the *BiocViews* links for DifferentialSplicing and AlternativeSplicing. For more information about the Bioconductor project and its core infrastructure, please refer to the overview by Huber *et al*. ^15^.

We note that there are numerous other methods for detecting differential transcript usage outside of the Bioconductor project. The *DRIMSeq* publication is a good reference for these, having descriptions and comparisons with many current methods ^11^. This workflow will build on the methods and vignettes from three Bioconductor packages: *DRIMSeq*, *DEXSeq*, and *stageR*. This Bioconductor workflow article does not contain any new statistical methods for detection of DTU or DGE, but instead leverages existing statistical methods and software packages.

Previously, some of the developers of the Bioconductor packages *edgeR* and *DESeq2* have collaborated to develop the *tximport* package ^16^ for summarizing the output of fast transcript-level quantifiers, such as *Salmon* ^17^, *Sailfish* ^18^, and *kallisto* ^19^. The *tximport* package focuses on preparing estimated transcript-level counts, abundances and effective transcript lengths, for gene-level statistical analysis using *edgeR* ^7^, *DESeq2* ^20^ or *limma-voom* ^10^. *tximport* produces an offset matrix to accompany gene-level counts, that accounts for a number of RNA-seq biases as well as differences in transcript usage among transcripts of different length that would bias an estimator of gene fold change based on the gene-level counts ^21^. *tximport* can alternatively produce a matrix of data that is roughly on the scale of counts, by scaling transcript-per-million (TPM) abundances to add up to the total number of mapped reads. This counts-from-abundance approach directly corrects for technical biases and differential transcript usage across samples, obviating the need for the accompanying offset matrix.

Complementary to an analysis of differential gene expression, one can use *tximport* to import transcript-level estimated counts, and then pass these counts to packages such as *DRIMSeq* or *DEXSeq* for statistical analysis of differential transcript usage. Following a transcript-level analysis, one can aggregate evidence of differential transcript usage to the gene level. The *stageR* package in Bioconductor provides a statistical framework to *screen* at the gene level for differential transcript usage with gene-level adjusted p-values, followed by *confirmation* of which transcripts within the significant genes show differential usage with transcript-level adjusted p-values ^12^. The method controls the *overall false discovery rate* (OFDR) ^22^ for such a two-stage procedure, which will be discussed in more detail later in the workflow. We believe that *stageR* represents a principled approach to analyzing transcript usage changes, as the methods can be evaluated against a target error rate in a manner that mimics how the methods will be used in practice. That is, following rejection of the null hypothesis at the gene level, investigators would likely desire to know which transcripts within a gene participated in the differential usage.

**Figure 1.**
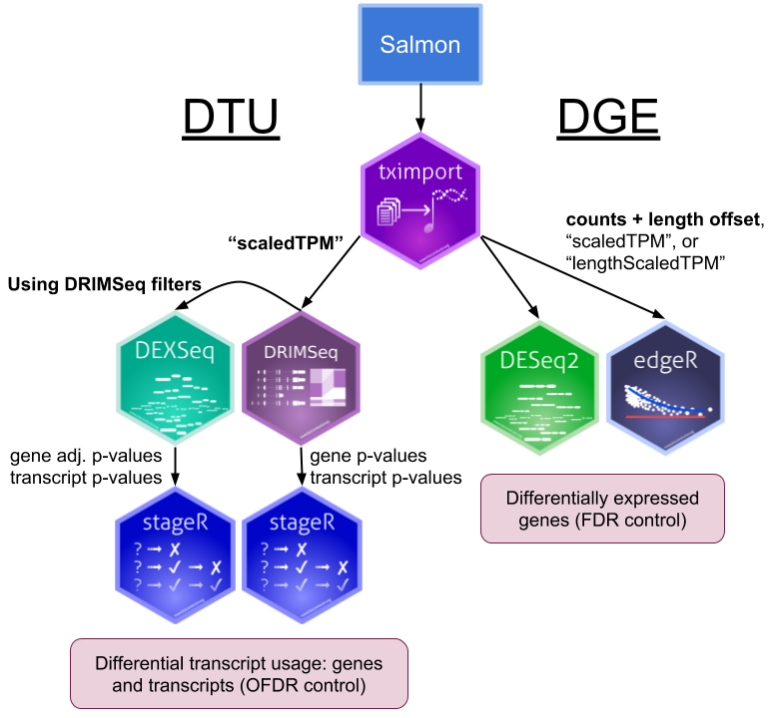
Diagram of the methods presented in this workflow. The left side shows two paths for performing differential transcript usage (DTU) using Bioconductor packages and the right side shows two paths for performing differential gene expression (DGE). DTU and DGE are complementary analyses of changes in transcription across conditions. This workflow focuses mostly on DTU, as there are a number of other published Bioconductor workflows for DGE. In bold are the recommended choices for quantification and filtering Salmon transcript-level data as input to the statistical methods. The recommended filters implemented in DRIMSeq, and applied upstream of DRIMSeq and DEXSeq, are discussed in this workflow.

Here we provide a basic workflow for detecting differential transcript usage using Bioconductor packages, following quantification of transcript abundance using the *Salmon* method ( Figure 1). This workflow includes live, runnable code chunks for analysis using *DRIMSeq* and *DEXSeq*, as well as for performing stage-wise testing of differential transcript usage using the *stageR* package. For the workflow, we use data that is simulated, so that we can also evaluate the performance of methods for differential transcript usage, as well as differential gene and transcript expression. The simulation was constructed using distributional parameters estimated from the GEUVADIS project RNA-seq dataset ^23^ quantified by the *recount2* project ^24^, including the expression levels of the transcripts, the amount of biological variability of gene expression levels across samples, and realistic coverage of reads along the transcripts.

### Structure of this article

1. In the Methods, we describe the simulation dataset, the quantification data generated by *Salmon* and imported via *tximport*, and the two statistical models for DTU, *DRIMSeq* and *DEXSeq*, that are highlighted in this workflow.
2. We present an end-to-end Workflow for detection of DTU, starting from sequenced reads files (FASTQ) and ending with sets of genes and transcripts determined to exhibit evidence of DTU by the statistical methods, DRIMSeq and DEXSeq. We demonstrate how *stageR* can be used with the output of DRIMSeq or DEXSeq to control the OFDR across genes and transcripts. Finally, we present code for performing differential gene expression (DGE) analysis using DESeq2 and edgeR, and show how to create a scatter plot that compares DTU and DGE results across all genes.
3. We present an Evaluation of the methods presented in the workflow along with other popular methods for detection of DTU, DGE, and differential transcript expression (DTE) on the simulated data. While the evaluations rely on simulated data, and are therefore relevant only to the extent that the simulation model and parameters reflect characteristics of real data, we feel the evaluations are useful for a rough comparison of method performance, and for observing relative changes in performance for a given method as sample size increases.
4. We conclude with a Discussion of the methods used in the workflow, including benefits and limitations, and our set of recommendations from the evaluation of the simulated data.

## Methods

### Simulation

First we describe details of the simulated data, which will be used in the following workflow and in the evaluation of methods. Understanding the details of the simulation will be useful for assessing the methods in the later sections. All of the code used to simulate RNA-seq experiments and write paired-end reads to FASTQ files can be found at an associated GitHub repository for the simulation code ^25^, and the reads and quantification files can be downloaded from Zenodo ^26– 29^. *Salmon* ^17^ was used to estimate transcript-level abundances for a single sample ( ERR188297) of the GEUVADIS project ^23^, and this was used as a baseline for transcript abundances in the simulation. Transcripts that were associated with estimated counts less than 10 had abundance thresholded to 0, all other transcripts were considered “expressed” (n=46,933). *alpine* ^30^ was used to estimate realistic fragment GC bias from 12 samples from the GEUVADIS project, all from the same sequencing center (the first 12 samples from CNAG-CRG in Supplementary Table 2 from Love *et al*. ^30^). *DESeq2* ^20^ was used to estimate mean and dispersion parameters for a Negative Binomial distribution for gene-level counts for 458 non-duplicated GEUVADIS samples provided by the *recount2* project ^24^, accounting for variation associated with sequencing center and human population. Note that, while gene-level dispersion estimates were used to generate underlying transcript-level counts, additional uncertainty on the transcript-level data is a natural consequence of the simulation, as the transcript-level counts must be estimated (the underlying transcript counts are not provided to the methods).

*polyester* ^31^ was used to simulate paired-end RNA-seq reads for two groups of 12 samples each, with realistic fragment GC bias, and with dispersion on transcript-level counts drawn from the joint distribution of mean and dispersion values estimated from the GEUVADIS samples. We will call this the “main simulation”. To compare *DRIMSeq* and *DEXSeq* in further detail, we generated an additional simulation in which dispersion parameters were assigned to genes via matching on the gene-level count, and then all transcripts of a gene had counts generated using the same per-gene dispersion. We will call this the “fixed per-gene dispersion” simulation. The first sample for group 1 and the first sample for group 2 followed the realistic GC bias profile of the same GEUVADIS sample, and so on for all 12 samples. This pairing of the samples was used to generate balanced data, but not used in the statistical analysis. *countsimQC* ^32^ was used to examine the properties of the simulation relative to the dataset used for parameter estimation ( Supplementary Figure 1). The simulation contains 24 samples, and the relevant parameters for *countsimQC* (per-gene mean and dispersion) were estimated over 458 samples. The full *countsimQC* report can be accessed at the associated GitHub repository for simulation code ^25^.

**Figure 2.**
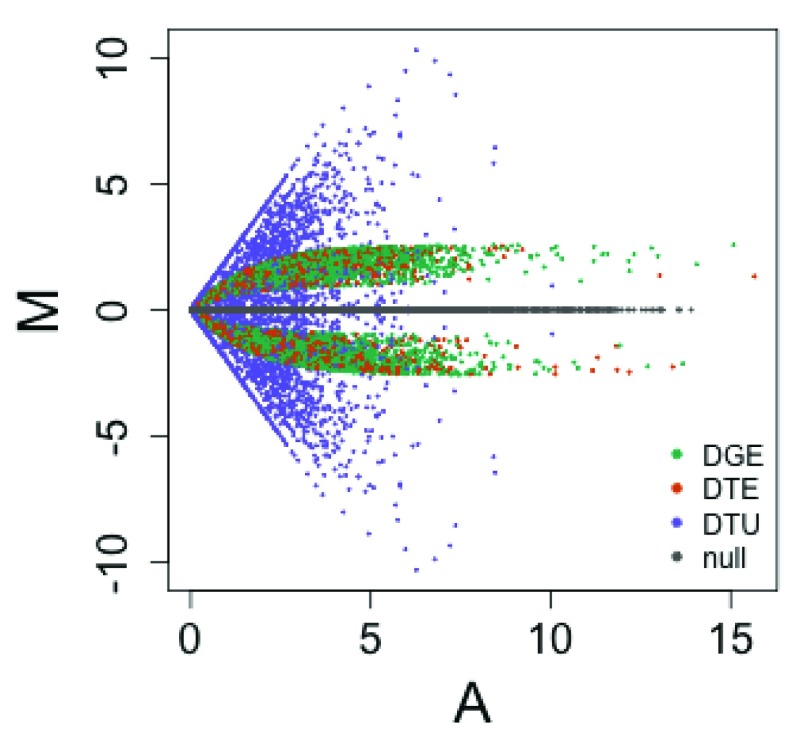
MA plot of simulated abundances. Each point depicts a transcript, with the average log2 abundance in transcripts-per-million (TPM) on the x-axis and the difference between the two groups on the y-axis. Of the 35,850 transcripts which are expressed with TPM > 1 in at least one group, 77% (n=27,429) are null transcripts (grey), which fall by construction on the M=0 line, and 23% (n=8,421) are differentially expressed (green, orange, or purple). This filtering of 1 TPM is for visualization only and unrelated to the DRIMSeq filtering used in the workflow. As transcripts can belong to multiple categories of differential gene expression (DGE), differential transcript expression (DTE), and differential transcript usage (DTU), here the transcripts are colored by which genes they belong to (those selected to be DGE-, DTE-, or DTU-by-construction).

Differential expression across two groups was generated as follows: The 46,933 expressed transcripts as defined above belonged to 15,017 genes. 70% (n=10,514) of these genes with expressed transcripts were set as null genes, where abundance was not changed across the two groups. For 10% (n=1,501) of genes, all isoforms were differentially expressed at a log fold change between 1 and 2.58 (fold change between 2 and 6). The set of transcripts in these genes was classified as DGE (differential gene expression) by construction, and the expressed transcripts were also DTE (differential transcript expression), but they did not count as DTU (differential transcript usage), as the proportions within the gene remained constant. To simulate balanced differential expression, one of the two groups was randomly chosen to be the baseline, and the other group would have its counts multiplied by the fold change. For 10% (n=1,501) of genes, a single expressed isoform was differentially expressed at a log fold change between 1 and 2.58. This set of transcripts was DTE by construction. If the chosen transcript was the only expressed isoform of a gene, this counted also as DGE and not as DTU, but if there were other isoforms that were expressed, this counted for both DGE and DTU, as the proportion of expression among the isoforms was affected. For 10% (n=1,501) of genes, differential transcript usage was constructed by exchanging the TPM abundance of two expressed isoforms, or, if only one isoform was expressed, exchanging the abundance of the expressed isoform with a non-expressed one. This counted for DTU and DTE, but not for DGE. An MA plot of the simulated transcript abundances for the two groups is shown in Figure 2.

### Quantification and data import

As described in the Introduction, this workflow uses transcript-level quantification estimates produced by *Salmon* ^17^ and imported into R/Bioconductor with *tximport* ^16^. Details about how to run *Salmon*, and which type of transcript-level estimated counts should be imported, is covered in the Workflow, with the exact code used to run the DTU analysis. *Salmon* estimates the relative abundance of each annotated transcript for each sample in units of transcripts-per-million (TPM); the estimated TPM values should be proportional to the abundance of the transcripts in the population of cells that were assayed. One critical point is that *Salmon* only considers the transcripts that are provided in the annotation; it is not able to detect expression of any novel transcripts. If many un-annotated transcripts are expressed in the particular set of samples, successful application of this workflow may require first building out a more representative set of annotated transcripts via transcriptome assembly or full transcript sequencing.

In addition to relative abundance, *Salmon* estimates the *effective length* of each transcript, which can take into account a number of sample-specific technical biases including fragment size distribution (default), fragment GC content, random hexamer priming bias, and positional bias along the transcript. If a transcript had certain properties, related to its length and its sequence content, that made it difficult to produce cDNA fragments and sequence reads from these fragments, then its effective length should be lower for that sample, than if these technical biases were absent. The estimates of TPM and the effective lengths can be used to estimate the number of fragments that each transcript produced, which will be called *estimated counts* in this workflow.

Different types of estimated counts may be correlated with effective transcript length, and so we will discuss in the Workflow our recommended type to use for DTU and DGE analysis (also diagrammed in Figure 1).

### DTU testing

We focus on two statistical models for DTU testing in this workflow, *DRIMSeq* ^11^ and *DEXSeq* ^6^. *DEXSeq* was published first, as a statistical model for detecting differences in exon usage across samples in different conditions. Most of the *DEXSeq* functions and documentation refer specifically to *exons* or *exonic parts* within a *gene*, while the final results table refers more generally to these as *features* within a *group*. It is this more general usage that we will employ in this workflow, substituting estimated transcript counts in place of exonic part counts.

*DEXSeq* assumes a Negative Binomial (NB) distribution for the feature counts, and considers the counts for each feature (originally, the exonic parts) relative to counts for all other features of the group (the gene), using an interaction term in a generalized linear model (GLM). The GLM framework is an extension of the linear model (LM), but shares with LM the usage of a design matrix, typically represented by **X**, which is made up of columns of covariates that are multiplied by scalar coefficients, typically represented by *β*. The design matrix with its multiple coefficients is useful for extending statistical models beyond simple group comparisons, allowing for more complex situations, such as within-patient comparisons, batch correction, or testing of ratios.

*DEXSeq* models each feature independently in the GLM fitting stage, and so does not take into account any correlation among the counts across features in a group (e.g. transcript counts within a gene), except insofar as such correlation is accounted for by columns in the design matrix. This last point is important, as correlation induced by DTU across condition groups, or even DTU that can be associated with cell-type heterogeneity, can be modeled in the *DEXSeq* framework by interaction terms with relevant covariates introduced into the design matrix. In the current workflow, we provide an example of capturing DTU across condition using *DEXSeq*, but future iterations of this workflow may also include controlling for additional covariates, such as estimated cell type proportions. *DEXSeq* was evaluated on transcript counts by Soneson *et al*. ^33^ and then later by Nowicka and Robinson ^11^, where it was shown in both cases that *DEXSeq* could be used to detect DTU in this configuration, though isoform pre-filtering greatly improved its performance in reducing the observed false discovery rate (FDR) closer to its nominal level.

In contrast to the NB model, *DRIMSeq* assumes an Dirichlet Multinomial model (DM) for each gene, where the total count for the gene is considered fixed, and the quantity of interest is the proportion for the transcript within a gene for each sample. If the proportion for one transcript increases, it must result in a decrease for the proportions of the other transcripts of the gene. Genes that are detected as having statistically significant DTU are those in which the proportion changes observed across condition were large, considering the variation in proportions seen within condition. The variation in proportions across biological replicates is modeled using a single *precision* parameter per gene, which will be discussed in the workflow below. *DRIMSeq* also uses a design matrix, and so can be used to analyze DTU for complex experimental designs, including within-patient comparisons, batch correction, or testing of ratios.

A critical difference between the two models is that *DRIMSeq* directly models the correlations among transcript counts within a gene via the DM distribution, and so can capture these correlations across exchangeable samples *within a condition*. *DEXSeq* with the NB distribution only can capture correlations among transcript counts within a gene when the DTU is *across sample groups* defined by covariates in the design matrix. On the other hand, *DRIMSeq* is limited in modeling a single precision parameter per gene. If there are two transcripts within a gene with very different biological variability – perhaps they have separate promoters under different regulatory control – then *DEXSeq* may do a better job modeling such heterogeneity in the biological variability of transcript expression, as it estimates a separate dispersion parameter for each transcript.

### Operation

This workflow was designed to work with R 3.5, and the *DRIMSeq*, *DEXSeq*, *stageR*, and *tximport* packages from Bioconductor version 3.7. As the code for this article is linked to Bioconductor version 3.7 (released April 2018), please consult the live Bioconductor workflow as the correct code for running the packages may change over time. Bioconductor packages should always be installed following the official instructions. The workflow uses a subset of all genes to speed up the analysis, but the Bioconductor packages can easily be run for this dataset on all human genes on a laptop in less than an hour. Compute time for the various packages is included in the respective evaluation sections.

## Workflow

### Salmon quantification

We used *Salmon* version 0.10.0 to quantify abundance and effective transcript lengths for all of the 24 simulated samples. For this workflow, we will use the first six samples from each group. We quantified against the GENCODE human annotation version 28, which was the same reference used to generate the simulated reads. We used the transcript sequences FASTA file that contains “Nucleotide sequences of all transcripts on the reference chromosomes”. When downloading the FASTA file, it is useful to download the corresponding GTF file, as this will be used in later sections.

To build the *Salmon* index, we used the following command. Recent versions of *Salmon* will discard identical sequence duplicate transcripts, and keep a log of these within the index directory. The –gencode command trims the GENCODE FASTA headers to only include the transcript identifier.

salmon index --gencode -t gencode.v28.transcripts.fa \
      -i gencode.v28_salmon-0.10.0

To quantify each sample, we used the following command, which says to quantify with six threads using the GENCODE index, with inward and unstranded paired end reads, using fragment GC bias correction, writing out to the directory sample and using as input these two reads files. The library type is specified by -l IU (inward and unstranded) and the options are discussed in the Salmon documentation. Recent versions of Salmon can automatically detect the library type by setting -l A. Such a command can be automated in a bash loop using bash variables, or one can use more advanced workflow management systems such as Snakemake ^34^ or Nextflow ^35^.

salmon quant -p 6 -i gencode.v28_salmon-0.10.0 -l IU \
      --gcBias -o sample -1 sample_1.fa.gz -2 sample_2.fa.gz

### Importing counts into R/Bioconductor

We can use *tximport* to import the estimated counts, abundances and effective transcript lengths into R. The *tximport* package offers three methods for producing count matrices from transcript-level quantification files, as described in detail in Soneson *et al*. ^16^, and already mentioned in the introduction. To recap these are: (1) original estimated counts with effective transcript length information used as a statistical offset, (2) generating “counts from abundance” by scaling TPM abundance estimates per sample such that they sum to the total number of mapped reads ( *scaledTPM*), or generating “counts from abundance” by scaling TPM abundance estimates first by the average effective transcript length over samples, and then per sample such that they sum to the total number of mapped reads ( *lengthScaledTPM*). We will use *scaledTPM* for DTU detection, with the statistical motivation described below, and the original estimated counts with length offset for DGE detection.

We recommend constructing a CSV file that keeps track of the sample identifiers and any relevant variables, e.g. condition, time point, batch, and so on. Here we have made a sample CSV file and provided it along with this workflow’s R package, *rnaseqDTU*. If a user is applying the code in this workflow with her own RNA-seq data, she does not need to load the *rnaseqDTU* package. If a user is running through the code in this workflow with the workflow simulated data, she does need to load the *rnaseqDTU* package.

In order to find this CSV file, we first need to know where on the machine this workflow package lives, so we can point to the extdata directory where the CSV file is located. These two lines of code load the workflow package and find this directory on the machine. Again, these two lines of code would therefore not be part of a *typical* analysis using one’s own RNA-seq data.

library(rnaseqDTU)
csv.dir <- system.file("extdata", package="rnaseqDTU")

The CSV file records which samples are condition 1 and which are condition 2. The columns of this CSV file can have any names, although sample_id will be used later by *DRIMSeq*, and so using this column name allows us to pass this *data.frame* directly to *DRIMSeq* at a later step.

samps <- read.csv(file.path(csv.dir, "samples.csv"))
head(samps)

##   sample_id condition
## 1      s1_1         1
## 2      s2_1         1
## 3      s3_1         1
## 4      s4_1         1
## 5      s5_1         1
## 6      s6_1         1

samps$condition <- factor(samps$condition)
table(samps$condition)

##
## 1 2
## 6 6

files <- file.path("/path/to/dir", samps$sample_id, "quant.sf")
names(files) <- samps$sample_id
head(files)

##                         s1_1                         s2_1
## "/path/to/dir/s1_1/quant.sf" "/path/to/dir/s2_1/quant.sf"
##                         s3_1                         s4_1
## "/path/to/dir/s3_1/quant.sf" "/path/to/dir/s4_1/quant.sf"
##                         s5_1                         s6_1
## "/path/to/dir/s5_1/quant.sf" "/path/to/dir/s6_1/quant.sf"

We can then import transcript-level counts using *tximport*. For DTU analysis, we suggest generating counts from abundance, using the scaledTPM method described by Soneson *et al*. ^16^. The countsFromAbundance option of *tximport* uses estimated abundances to generate roughly count-scaled data, such that each column will sum to the number of reads mapped for that library. By using scaledTPM counts, the estimated proportions fit by *DRIMSeq*, which are generated from counts, will be equivalent to proportions of the abundance of the isoforms.

If instead of scaledTPM, we used the original estimated transcript counts ( countsFromAbundance="no"), or if we used lengthScaledTPM transcript counts, then a change in transcript usage among transcripts of different length could result in a changed total count for the gene, even if there is no change in total gene expression. For more detail and a diagram of this effect, we refer the reader to Figure 1 of Trapnell *et al*. ^21^. Briefly, this is because the original transcript counts and lengthScaledTPM transcript counts scale with transcript length, while scaledTPM transcript counts do not. A change in the total count for the gene, not corrected by an offset, could then bias the calculation of proportions and therefore confound DTU analysis. For testing DTU using *DRIMSeq* and *DEXSeq*, it is convenient if the count-scale data do *not* scale with transcript length within a gene. This could be corrected by an offset, but this is not easily implemented in the current DTU analysis packages. For *gene-level* analysis (DGE), we can use gene-level original counts with an average transcript length offset, gene-level scaledTPM counts, or gene-level lengthScaledTPM counts, as all of these three methods control for the length bias described by Trapnell *et al*. ^21^ and Soneson *et al*. ^16^. Our DTU and DGE countsFromAbundance recommendations are summarized in Figure 1.

A final note is that, the motivation for using scaledTPM counts hinges on the fact that estimated fragment counts scale with transcript length in fragmented RNA-seq data. If a different experiment is performed and a different quantification method used to produce counts per transcript which *do not* scale with transcript length, then the recommendation would be to use these counts per transcript directly. Examples of experiments producing counts per transcript that would potentially not scale with transcript length include counts of full-transcript-length or nearly-full-transcript-length reads, or counts of 3’ tagged RNA-seq reads aggregated to transcript groups. In either case, the statistical methods for DTU could be provided directly with the transcript counts.

The following code chunk is what one would use in a typical analysis, but is not evaluated in this workflow because the quantification files are not provided in the *rnaseqDTU* package due to size restrictions. Instead we will load a pre-constructed matrix of counts below. In a typical workflow, the code below would be used to generate the matrix of counts from the quantification files. All of the quantification files and simulated reads for this dataset have been made publicly available on *Zenodo*; see the *Data availability* section at the end of this workflow.

library(tximport)
txi <- tximport(files, type="salmon", txOut=TRUE,
                  countsFromAbundance="scaledTPM")
cts <- txi$counts
cts <- cts[rowSums(cts) > 0,]

### Transcript-to-gene mapping

Bioconductor offers numerous approaches for building a *TxDb* object, a transcript database that can be used to link transcripts to genes (among other uses). The following code chunks were used to generate a *TxDb*, and then use the select function with the *TxDb* to produce a corresponding *data.frame* called txdf which links transcript IDs to gene IDs. In this *TxDb*, the transcript IDs are called TXNAME and the gene IDs are called GENEID. The version 28 human GTF file was downloaded from the GENCODE website when downloading the transcripts FASTA file. Due to size restrictions, neither the gencode.v28.annotation.gtf.gz file nor the generated .sqlite file are included in the *rnaseqDTU* package.

library(GenomicFeatures)
gtf <- "gencode.v28.annotation.gtf.gz"
txdb.filename <- "gencode.v28.annotation.sqlite"
txdb <- makeTxDbFromGFF(gtf)
saveDb(txdb, txdb.filename)

Once the *TxDb* database has been generated and saved, it can be quickly reloaded:

txdb <- loadDb(txdb.filename)
txdf <- select(txdb, keys(txdb, "GENEID"), "TXNAME", "GENEID")
tab <- table(txdf$GENEID)
txdf$ntx <- tab[match(txdf$GENEID, names(tab))]

### DRIMSeq

We load the cts object as created in the *tximport* code chunks. This contains count-scale data, generated from abundance using the scaledTPM method. The column sums are equal to the number of mapped paired-end reads per experiment. The experiments have between 31 and 38 million paired-end reads that were mapped to the transcriptome using *Salmon*.

data(salmon_cts)
cts[1:3,1:3]

##                         s1_1       s2_1       s3_1
## ENST00000488147.1 179.798908 184.437348 229.046306
## ENST00000469289.1   0.000000   0.000000   0.000000
## ENST00000466430.5   5.004159   3.627831   9.463167

range(colSums(cts)/1e6)

## [1] 31.37738 38.47173

We also have the txdf object giving the transcript-to-gene mappings (for construction, see previous section). This is contained in a file called simulate.rda that contains a number of R objects with information about the simulation, that we will use later to assess the methods’ performance.

data(simulate)
head(txdf)

##               GENEID            TXNAME ntx
## 1 ENSG00000000003.14 ENST00000612152.4   5
## 2 ENSG00000000003.14 ENST00000373020.8   5
## 3 ENSG00000000003.14 ENST00000614008.4   5
## 4 ENSG00000000003.14 ENST00000496771.5   5
## 5 ENSG00000000003.14 ENST00000494424.1   5
## 6  ENSG00000000005.5 ENST00000373031.4   2

all(rownames(cts) %in% txdf$TXNAME)

## [1] TRUE

txdf <- txdf[match(rownames(cts),txdf$TXNAME),]
all(rownames(cts) == txdf$TXNAME)

## [1] TRUE

In order to run *DRIMSeq*, we build a *data.frame* with the gene ID, the feature (transcript) ID, and then columns for each of the samples:

counts <- data.frame(gene_id=txdf$GENEID,
                       feature_id=txdf$TXNAME,
                       cts)

We can now load the *DRIMSeq* package and create a *dmDSdata* object, with our counts and samps *data.frames*. Typing in the object name and pressing return will give information about the number of genes:

library(DRIMSeq)
d <- dmDSdata(counts=counts, samples=samps)
d

## An object of class dmDSdata
## with 16612 genes and 12 samples
## * data accessors: counts(), samples()

The *dmDSdata* object has a number of specific methods. Note that the rows of the object are gene-oriented, so pulling out the first *row* corresponds to all of the transcripts of the first gene:

methods(class=class(d))

## [1] [           coerce      counts      dmFilter      dmPrecision length
## [7] names       plotData    show
## see ’?methods’ for accessing help and source code

counts(d[1,])[,1:4]

##              gene_id        feature_id       s1_1       s2_1
## 1 ENSG00000000419.12 ENST00000371588.9 1394.71411 1210.12539
## 2 ENSG00000000419.12 ENST00000466152.5  135.15850   18.20031
## 3 ENSG00000000419.12 ENST00000371582.8  154.77943   35.39425
## 4 ENSG00000000419.12 ENST00000371584.8   42.85733   86.04958
## 5 ENSG00000000419.12 ENST00000413082.1    0.00000    0.00000

It will be useful to first filter the object, before running procedures to estimate model parameters. This greatly speeds up the fitting and removes transcripts that may be troublesome for parameter estimation, e.g. estimating the proportion of expression among the transcripts of a gene when the total count is very low. We first define n to be the total number of samples, and n.small to be the sample size of the smallest group. We use all three of the possible filters: for a transcript to be retained in the dataset, we require that (1) it has a count of at least 10 in at least n.small samples, (2) it has a relative abundance proportion of at least 0.1 in at least n.small samples, and (3) the total count of the corresponding gene is at least 10 in all n samples. We used all three possible filters, whereas only the two count filters are used in the *DRIMSeq* vignette example code.

It is important to consider what types of transcripts may be removed by the filters, and potentially adjust depending on the dataset. If n was large, it would make sense to allow perhaps a few samples to have very low counts, so lowering min_samps_gene_expr to some factor multiple ( *<* 1) of n, and likewise for the first two filters for n.small. The second filter means that if a transcript does not make up more than 10% of the gene’s expression for at least n.small samples, it will be removed. If this proportion seems too high, for example, if very lowly expressed isoforms are of particular interest, then the filter can be omitted or the min_feature_prop lowered. For a concrete example, if a transcript goes from a proportion of 0% in the control group to a proportion of 9% in the treatment group, this would be removed by the above 10% filter. After filtering, this dataset has 7,764 genes.

n <- 12
n.small <- 6
d <- dmFilter(d,
                min_samps_feature_expr=n.small, min_feature_expr=10,
                min_samps_feature_prop=n.small, min_feature_prop=0.1,
                min_samps_gene_expr=n, min_gene_expr=10)
d

## An object of class dmDSdata
## with 7764 genes and 12 samples
## * data accessors: counts(), samples()

The *dmDSdata* object only contains genes that have more that one isoform, which makes sense as we are testing for differential transcript usage. We can find out how many of the remaining genes have *N* isoforms by tabulating the number of times we see a gene ID, then tabulating the output again:

table(table(counts(d)$gene_id))

##
##    2    3    4    5    6    7
## 4062 2514  931  222   34    1

We create a design matrix, using a design formula and the sample information contained in the object, accessed via *samples*. Here we use a simple design with just two groups, but more complex designs are possible. For some discussion of complex designs, one can refer to the vignettes of the *limma*, *edgeR*, or *DESeq2* packages.

design_full <- model.matrix(~condition, data=DRIMSeq::samples(d))
colnames(design_full)

## [1] "(Intercept)" "condition2"

Only for speeding up running the live code chunks in this workflow, we subset to the first 250 genes, representing about one thirtieth of the dataset. This step would not be run in a typical workflow.

d <- d[1:250,]
7764 / 250

## [1] 31.056

We then use the following three functions to estimate the model parameters and test for DTU. We first estimate the *precision*, which is related to the dispersion in the Dirichlet Multinomial model via the formula below. Because precision is in the denominator of the right hand side of the equation, they are inversely related. Higher *dispersion* – counts more variable around their expected value – is associated with lower *precision*. For full details about the *DRIMSeq* model, one should read both the detailed software vignette and the publication ^11^. After estimating the precision, we fit regression coefficients and perform null hypothesis testing on the coefficient of interest. Because we have a simple two-group model, we test the coefficient associated with the difference between condition 2 and condition 1, called condition2. The following code takes about half a minute, and so a full analysis on this dataset takes about 15 minutes on a laptop.

$$\mathrm{dispersion}\;=\;\frac{1}{1+\mathrm{precision}}$$

set.seed(1)
system.time({
  d <- dmPrecision(d, design=design_full)
  d <- dmFit(d, design=design_full)
  d <- dmTest(d, coef="condition2")
})

## ! Using a subset of 0.1 genes to estimate common precision !

## ! Using common_precision = 21.2862 as prec_init !

## ! Using 0 as a shrinkage factor !

##    user  system elapsed
##  34.213   0.450  35.846

To build a results table, we run the results function. We can generate a single p-value per gene, which tests whether there is any differential transcript usage within the gene, or a single p-value per transcript, which tests whether the proportions for this transcript changed within the gene:

res <- DRIMSeq::results(d)
head(res)

##              gene_id        lr df       pvalue   adj_pvalue
## 1 ENSG00000000457.13  1.493561  4 8.277814e-01 9.120246e-01
## 2 ENSG00000000460.16  1.068294  3 7.847330e-01 9.101892e-01
## 3 ENSG00000000938.12  4.366806  2 1.126575e-01 2.750169e-01
## 4 ENSG00000001084.11  1.630085  3 6.525877e-01 8.643316e-01
## 5 ENSG00000001167.14 28.402587  1 9.853354e-08 5.007113e-07
## 6 ENSG00000001461.16  9.815460  1 1.730510e-03 6.732766e-03

res.txp <- DRIMSeq::results(d, level="feature")
head(res.txp)

##              gene_id         feature_id         lr df    pvalue adj_pvalue
## 1 ENSG00000000457.13 ENST00000367771.10 0.16587607  1 0.6838032  0.9171007
## 2 ENSG00000000457.13  ENST00000367770.5 0.01666448  1 0.8972856  0.9788571
## 3 ENSG00000000457.13  ENST00000367772.8 1.02668495  1 0.3109386  0.6667146
## 4 ENSG00000000457.13  ENST00000423670.1 0.06046507  1 0.8057624  0.9323782
## 5 ENSG00000000457.13  ENST00000470238.1 0.28905766  1 0.5908250  0.8713427
## 6 ENSG00000000460.16  ENST00000496973.5 0.83415788  1 0.3610730  0.7232298

Because the pvalue column may contain NA values, we use the following function to turn these into 1’s. The NA values would otherwise cause problems for the stage-wise analysis. From investigating these NA p-value cases for *DRIMSeq*, they all occur when one condition group has all zero counts for a transcript, but sufficient counts from the other condition group, and sufficient counts for the gene. *DRIMSeq* will not estimate a precision for such a gene. These all happen to be true positive genes for DTU in the simulation, where the isoform switch is total or nearly total. *DEXSeq*, shown in a later section, does not produce NA p-values for these genes. A potential fix would be to use a plug-in common or trended precision for such genes, but this is not implemented in the current version of *DRIMSeq*.

no.na <- function(x) ifelse(is.na(x), 1, x)
res$pvalue <- no.na(res$pvalue)
res.txp$pvalue <- no.na(res.txp$pvalue)

**Figure 3.**
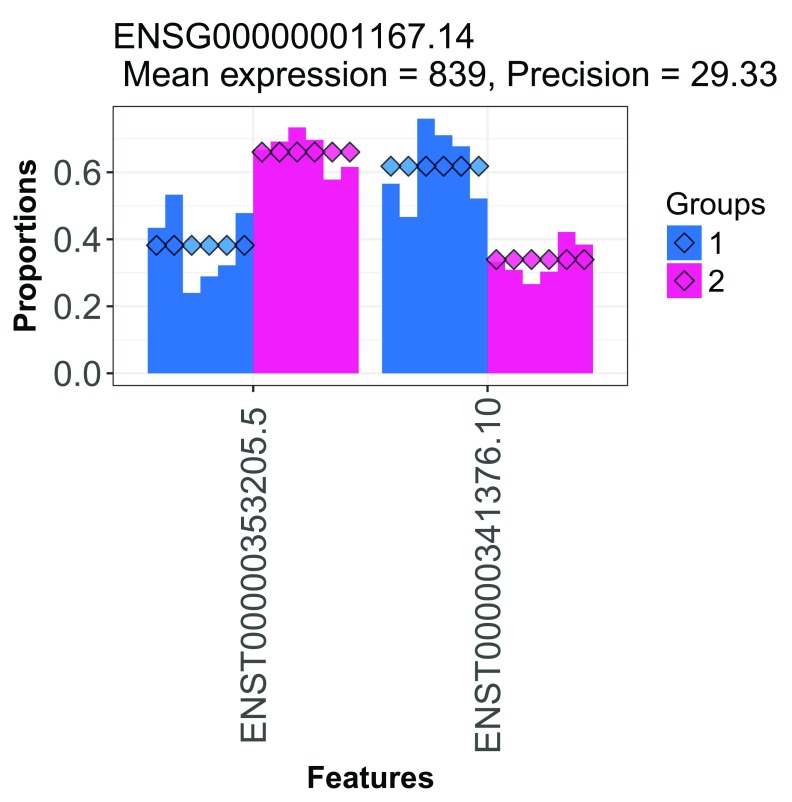
Estimated transcript proportions for one of the significant genes.

We can plot the estimated proportions for one of the significant genes, where we can see evidence of switching ( Figure 3).

idx <- which(res$adj_pvalue < 0.05)[1]
res[idx,]

##              gene_id       lr df       pvalue    adj_pvalue
## 5 ENSG00000001167.14 28.40259  1 9.853354e-08  5.007113e-07

plotProportions(d, res$gene_id[idx], "condition")

### stageR following DRIMSeq

Because we have been working with only a subset of the data, we now load the results tables that would have been generated by running *DRIMSeq* functions on the entire dataset.

data(drim_tables)
nrow(res)

## [1] 7764

nrow(res.txp)

## [1] 20711

A typical analysis of differential transcript usage would involve asking first: “which genes contain any evidence of DTU?”, and secondly, “which transcripts in the genes that contain some evidence may be participating in the DTU?” Note that a gene may pass the first stage without exhibiting enough evidence to identify one or more transcripts that are participating in the DTU. The *stageR* package is designed to allow for such two-stage testing procedures, where the first stage is called a *screening* stage and the second stage a *confirmation* stage ^12^. The methods are general, and can also be applied to testing, for example, changes across a time series followed by investigation of individual time points, as shown in the *stageR* package vignette. We show below how *stageR* is used to detect DTU and how to interpret its output.

We first construct a vector of p-values for the screening stage. Because of how the *stageR* package will combine transcript and gene names, we need to strip the gene and transcript version numbers from their Ensembl IDs (this is done by keeping only the first 15 characters of the gene and transcript IDs).

pScreen <- res$pvalue
strp <- function(x) substr(x,1,15)
names(pScreen) <- strp(res$gene_id)

We construct a one column matrix of the confirmation p-values:

pConfirmation <- matrix(res.txp$pvalue, ncol=1)
rownames(pConfirmation) <- strp(res.txp$feature_id)

We arrange a two column *data.frame* with the transcript and gene identifiers.

tx2gene <- res.txp[,c("feature_id", "gene_id")]
for (i in 1:2) tx2gene[,i] <- strp(tx2gene[,i])

The following functions then perform the *stageR* analysis. We must specify an alpha, which will be the *overall false discovery rate* target for the analysis, defined below. Unlike typical adjusted p-values or q-values, we cannot choose an arbitrary threshold later: after specifying alpha=0.05, we need to use 5% as the target in downstream steps. There are also convenience functions *getSignificantGenes* and *getSignificantTx*, which are demonstrated in the *stageR* vignette.

library(stageR)
stageRObj <- stageRTx(pScreen=pScreen, pConfirmation=pConfirmation,
                         pScreenAdjusted=FALSE, tx2gene=tx2gene)
stageRObj <- stageWiseAdjustment(stageRObj, method="dtu", alpha=0.05) 
suppressWarnings({
  drim.padj <- getAdjustedPValues(stageRObj, order=FALSE,
                                      onlySignificantGenes=TRUE)
})
head(drim.padj)


##            geneID            txID         gene transcript
## 1 ENSG00000001167 ENST00000341376 1.446731e-05   0.000000
## 2 ENSG00000001167 ENST00000353205 1.446731e-05   0.000000
## 3 ENSG00000001461 ENST00000003912 8.263160e-03   0.000000
## 4 ENSG00000001461 ENST00000339255 8.263160e-03   0.000000
## 5 ENSG00000001631 ENST00000394507 1.287012e-04   0.060474
## 6 ENSG00000001631 ENST00000475770 1.287012e-04   1.000000

The final table with adjusted p-values summarizes the information from the two-stage analysis. Only genes that passed the filter are included in the table, so the table already represents *screened* genes. The transcripts with values in the column, transcript, less than 0.05 pass the *confirmation* stage on a target 5% *overall false discovery rate*, or OFDR. This means that, in expectation, no more than 5% of the genes that pass screening will either (1) not contain any DTU, so be falsely screened genes, or (2) contain a falsely confirmed transcript. A falsely confirmed transcript is a transcript with an adjusted p-value less than 0.05 which does not exhibit differential usage across conditions. The *stageR* procedure allows us to look at both the genes that passed the screening stage and the transcripts with adjusted p-values less than our target alpha, and understand what kind of *overall* error rate this procedure entails. This cannot be said for an arbitrary procedure of looking at standard gene adjusted p-values and transcript adjusted p-values, where the adjustment was performed independently.

### Post-hoc filtering on the standard deviation in proportions

We found that *DRIMSeq* was sensitive to detect DTU, but could exceed its false discovery rate (FDR) bounds, particularly on the transcript-level tests, and that a post-hoc, non-specific filtering of the *DRIMSeq* transcript p-values and adjusted p-values improved the FDR and OFDR control. We considered the standard deviation (SD) of the per-sample proportions as a filtering statistic. This statistic does not use the information about which samples belong to which condition group. We set the p-values and adjusted p-values for transcripts with small per-sample proportion SD to 1. We do not recompute adjusted p-values, although we will provide the filtered p-values to the *stageR* procedure.

We note that the p-values are no longer necessarily uniform after filtering out small effect size transcripts and genes, although we find that in this simulation at least, the filtering made the procedure *more conservative*: excluding transcripts with small SD of the per-sample proportions brought both the observed FDR and the observed OFDR closer to their nominal targets, as will be shown in the evaluations below.

res.txp.filt <- DRIMSeq::results(d, level="feature")
smallProportionSD <- function(d, filter=0.1) {
  cts <- as.matrix(subset(counts(d), select=-c(gene_id, feature_id)))
  gene.cts <- rowsum(cts, counts(d)$gene_id)
  total.cts <- gene.cts[match(counts(d)$gene_id, rownames(gene.cts)),]
  props <- cts/total.cts
  propSD <- sqrt(rowVars(props))
  propSD < filter
}
filt <- smallProportionSD(d)
res.txp.filt$pvalue[filt] <- 1
res.txp.filt$adj_pvalue[filt] <- 1

The above post-hoc filter is not part of the *DRIMSeq* modeling steps, and to avoid interfering with the modeling, we run it after *DRIMSeq*. The other three filters used before have been tested by the *DRIMSeq* package authors, and are therefore a recommended part of an analysis before the modeling begins. We do not apply this post-hoc filter to *DEXSeq* in this workflow, as *DEXSeq* seemed to be closer to controlling its FDR and OFDR in the evaluations, after using the *DRIMSeq* filters recommended in this workflow.

### DEXSeq

The *DEXSeq* package was originally designed for detecting differential exon usage ^6^, but can also be adapted to run on estimated transcript counts, in order to detect DTU. Using *DEXSeq* on transcript counts was evaluated by Soneson *et al*. ^33^, showing the benefits in FDR control from filtering lowly expressed transcripts for a transcript-level analysis. We benchmarked *DEXSeq* here, beginning with the *DRIMSeq* filtered object, as these filters are intuitive, they greatly speed up the analysis, and such filtering was shown to be beneficial in FDR control.

The two factors of (1) working on isoform counts rather than individual exons and (2) using the *DRIMSeq* filtering procedure dramatically increase the speed of *DEXSeq*, compared to running an exon-level analysis. Another advantage is that we benefit from the sophisticated bias models of *Salmon*, which account for drops in coverage on alternative exons that can otherwise throw off estimates of transcript abundance ^30^. A disadvantage over the exon-level analysis is that we must know in advance all of the possible isoforms that can be generated from a gene locus, all of which are assumed to be contained in the annotation files (FASTA and GTF).

We first load the *DEXSeq* package and then build a *DEXSeqDataSet* from the data contained in the *dmDStest* object (the class of the *DRIMSeq* object changes as the results are added). The design formula of the *DEXSeq-DataSet* here uses the language “exon” but this should be read as “transcript” for our analysis. *DEXSeq* will test – after accounting for total gene expression for this sample and for the proportion of this transcript relative to the others – whether there is a condition-specific difference in the transcript proportion relative to the others.

library(DEXSeq)
sample.data <- DRIMSeq::samples(d)
count.data <- round(as.matrix(counts(d)[,-c(1:2)]))
dxd <- DEXSeqDataSet(countData=count.data,
                       sampleData=sample.data,
                       design=~sample + exon + condition:exon,
                       featureID=counts(d)$feature_id,
                       groupID=counts(d)$gene_id)

The following functions run the *DEXSeq* analysis. While we are only working on a subset of the data, the full analysis for this dataset took less than 3 minutes on a laptop.

system.time({
  dxd <- estimateSizeFactors(dxd)
  dxd <- estimateDispersions(dxd, quiet=TRUE)
  dxd <- testForDEU(dxd, reducedModel=~sample + exon)
})

##    user system elapsed
##   7.451  0.032   7.488

We then extract the results table, not filtering on mean counts (as we have already conducted filtering via *DRIMSeq* functions). We compute a per-gene adjusted p-value, using the *perGeneQValue* function, which aggregates evidence from multiple tests within a gene to a single p-value for the gene and then corrects for multiple testing across genes ^6^. Other methods for aggregative evidence from the multiple tests within genes have been discussed in a recent publication and may be substituted at this step ^36^. Finally, we build a simple results table with the per-gene adjusted p-values.

dxr <- DEXSeqResults(dxd, independentFiltering=FALSE)
qval <- perGeneQValue(dxr)
dxr.g <- data.frame(gene=names(qval),qval)

For size consideration of the workflow R package, we reduce also the transcript-level results table to a simple *data.frame*:

columns <- c("featureID","groupID","pvalue")
dxr <- as.data.frame(dxr[,columns])
head(dxr)
##	                                          featureID	          groupID
## ENSG00000000457.13:ENST00000367771.10 ENST00000367771.10    ENSG00000000457.13
## ENSG00000000457.13:ENST00000367770.5	  ENST00000367770.5    ENSG00000000457.13
## ENSG00000000457.13:ENST00000367772.8	  ENST00000367772.8    ENSG00000000457.13
## ENSG00000000457.13:ENST00000423670.1	  ENST00000423670.1    ENSG00000000457.13
## ENSG00000000457.13:ENST00000470238.1	  ENST00000470238.1    ENSG00000000457.13
## ENSG00000000460.16:ENST00000496973.5	  ENST00000496973.5    ENSG00000000460.16
##	                                    pvalue
## ENSG00000000457.13:ENST00000367771.10 0.5620081
## ENSG00000000457.13:ENST00000367770.5	 0.8399434
## ENSG00000000457.13:ENST00000367772.8	 0.5675043
## ENSG00000000457.13:ENST00000423670.1	 0.7032904
## ENSG00000000457.13:ENST00000470238.1	 0.8476920
## ENSG00000000460.16:ENST00000496973.5	 0.2108527

### stageR following DEXSeq

Again, as we have been working with only a subset of the data, we now load the results tables that would have been generated by running *DEXSeq* functions on the entire dataset.

data(dex_tables)

If the *stageR* package has not already been loaded, we make sure to load it, and run code very similar to that used above for *DRIMSeq* two-stage testing, with a target alpha=0.05.

library(stageR)
strp <- function(x) substr(x,1,15)
pConfirmation <- matrix(dxr$pvalue,ncol=1)
dimnames(pConfirmation) <- list(strp(dxr$featureID),"transcript")
pScreen <- qval
names(pScreen) <- strp(names(pScreen))
tx2gene <- as.data.frame(dxr[,c("featureID", "groupID")])
for (i in 1:2) tx2gene[,i] <- strp(tx2gene[,i])

The following three functions provide a table with the OFDR control described above. To repeat, the set of genes passing screening should not have more than 5% of either genes which have in fact no DTU or genes which contain a transcript with an adjusted p-value less than 5% which do not participate in DTU.

stageRObj <- stageRTx(pScreen=pScreen, pConfirmation=pConfirmation,
                        pScreenAdjusted=TRUE, tx2gene=tx2gene)
stageRObj <- stageWiseAdjustment(stageRObj, method="dtu", alpha=0.05)
suppressWarnings({
  dex.padj <- getAdjustedPValues(stageRObj, order=FALSE,
                                     onlySignificantGenes=TRUE)
})
head(dex.padj)

##            geneID            txID         gene transcript
## 1 ENSG00000001167 ENST00000341376 1.379695e-05         0
## 2 ENSG00000001167 ENST00000353205 1.379695e-05          0
## 3 ENSG00000001461 ENST00000003912 1.011322e-03          0
## 4 ENSG00000001461 ENST00000339255 1.011322e-03          0
## 5 ENSG00000001630 ENST00000003100 4.979296e-03         0
## 6 ENSG00000001630 ENST00000450723 4.979296e-03          0

### Citing methods in published research

This concludes the DTU section of the workflow. If you use *DRIMSeq* ^11^, *DEXSeq* ^6^, *stageR* ^12^, *tximport* ^16^, or *Salmon* ^17^ in published research, please cite the relevant methods publications, which can be found in the References section of this workflow.

### DGE analysis with DESeq2

In the final section of the workflow containing live code examples, we demonstrate how differential transcript usage, summarized to the gene level, can be visualized with respect to differential gene expression analysis results. We use *tximport* and summarize counts to the gene level and compute an average transcript length offset for count-based methods ^16^. We will then show code for using *DESeq2* and *edgeR* to assess differential gene expression. Because we have simulated the genes according to three different categories, we can color the final plot by the true simulated state of the genes. We note that we will pair *DEXSeq* with *DESeq2* results in the following plot, and *DRIMSeq* with *edgeR* results. However, this pairing is arbitrary, and any DTU method can reasonably be paired with any DGE method.

The following line of code is unevaluated, but was used to generate an object txi.g which contains the gene-level counts, abundances and average transcript lengths.

txi.g <- tximport(files, type="salmon", tx2gene=txdf[,2:1])

For the workflow, we load the txi.g object which is saved in a file salmon_gene_txi.rda. We then load the *DESeq2* package and build a *DESeqDataSet* from txi.g, providing also the sample information and a design formula.

data(salmon_gene_txi)
library(DESeq2)
dds <- DESeqDataSetFromTximport(txi.g, samps, ~condition)

## using counts and average transcript lengths from tximport

The following two lines of code run the *DESeq2* analysis ^20^.

dds <- DESeq(dds)
dres <- DESeq2::results(dds)

Because we happen to know the true status of each of the genes, we can make a scatterplot of the results, coloring the genes by their status (whether DGE, DTE, or DTU by construction).

all(dxr.g$gene %in% rownames(dres))


## [1] TRUE

dres <- dres[dxr.g$gene,]
# we can only color because we simulated...
col <- rep(8, nrow(dres))
col[rownames(dres) %in% dge.genes] <- 1
col[rownames(dres) %in% dte.genes] <- 2
col[rownames(dres) %in% dtu.genes] <- 3

**Figure 4.**
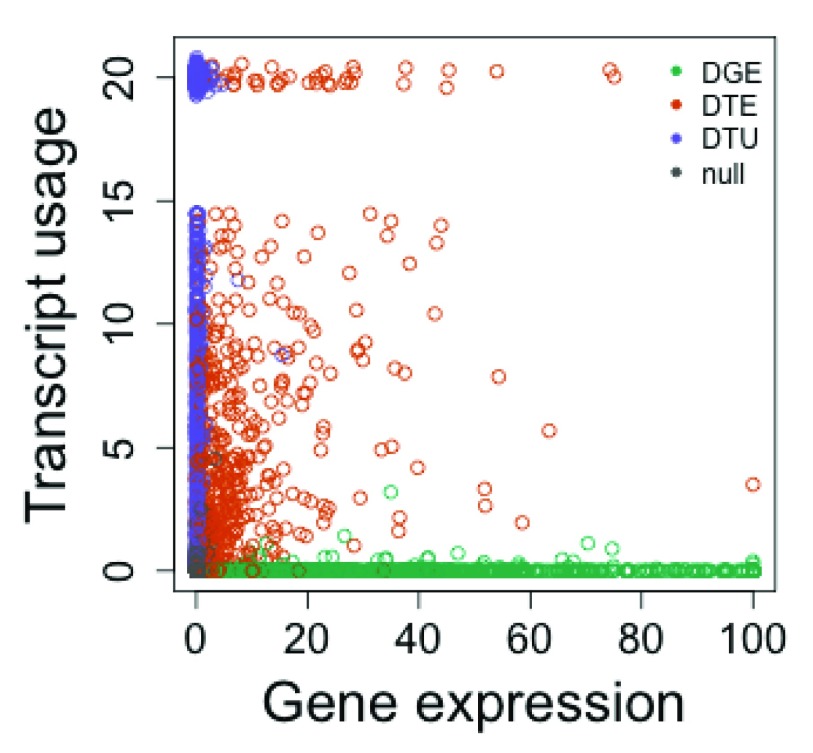
Transcript usage over gene expression plot. Each point represents a gene, and plotted are -log10 adjusted p-values for DEXSeq’s test of differential transcript usage (y-axis) and DESeq2’s test of differential gene expression (x-axis). Because we simulated the data we can color the genes according to their true category.

Figure 4 displays the evidence for differential transcript usage over that for differential gene expression. We can see that the DTU genes cluster on the y-axis (mostly not captured in the DGE analysis), and the DGE genes cluster on the x-axis (mostly not captured in the DTU analysis). The DTE genes fall in the middle, as all of them represent DGE, and some of them additionally represent DTU (if the gene had other expressed transcripts). Because *DEXSeq* outputs an adjusted p-value of 0 for some of the genes, we set these instead to a jittered value around 10 ^−20^, so that their number and location on the x-axis could be visualized. These jittered values should only be used for visualization.

bigpar()
# here cap the smallest DESeq2 adj p-value
cap.padj <- pmin(-log10(dres$padj), 100)
# this vector only used for plotting
jitter.padj <- -log10(dxr.g$qval + 1e-20)
jp.idx <- jitter.padj == 20
jitter.padj[jp.idx] <- rnorm(sum(jp.idx),20,.25)
plot(cap.padj, jitter.padj, col=col,
      xlab="Gene expression",
      ylab="Transcript usage")
legend("topright",
        c("DGE","DTE","DTU","null"),
        col=c(1:3,8), pch=20, bty="n")        

### DGE analysis with edgeR

We can also perform differential gene expression analysis using *edgeR* as the inference engine ^7^. The following code incorporates the average transcript length matrix as an offset for an *edgeR* analysis.

library(edgeR)
cts.g <- txi.g$counts
normMat <- txi.g$length
normMat <- normMat / exp(rowMeans(log(normMat)))
o <- log(calcNormFactors(cts.g/normMat)) + log(colSums(cts.g/normMat))
y <- DGEList(cts.g)
y <- scaleOffset(y, t(t(log(normMat)) + o))
keep <- filterByExpr(y)
y <- y[keep,]

The basic *edgeR* model fitting and results extraction can be accomplished with the following lines:

y <- estimateDisp(y, design_full)
fit <- glmFit(y, design_full)
lrt <- glmLRT(fit)
tt <- topTags(lrt, n=nrow(y), sort="none")[[1]]

Again, we can color the genes by their true status in the simulation:

common <- intersect(res$gene_id, rownames(tt))
tt <- tt[common,]
res.sub <- res[match(common, res$gene_id),]
# we can only color because we simulated...
col <- rep(8, nrow(tt))
col[rownames(tt) %in% dge.genes] <- 1
col[rownames(tt) %in% dte.genes] <- 2
col[rownames(tt) %in% dtu.genes] <- 3

**Figure 5.**
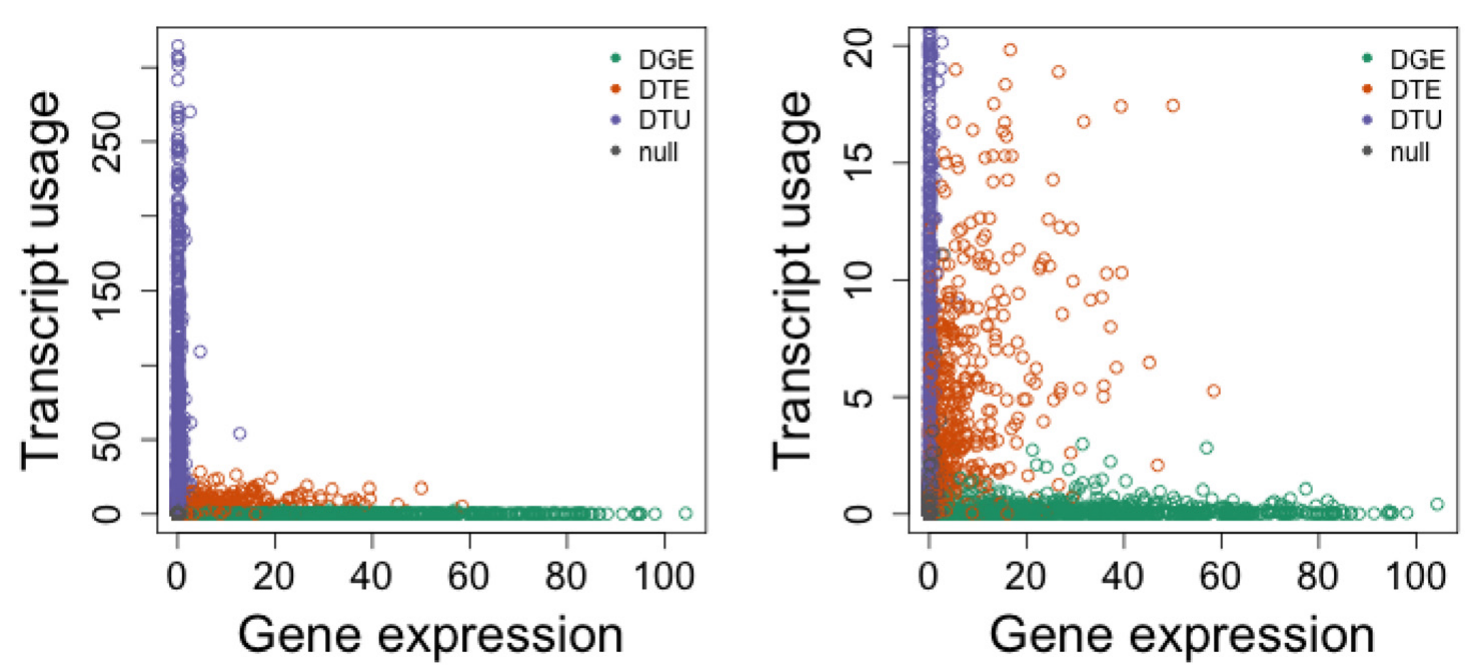
Transcript usage over gene expression plot, as previously, but for DRIMSeq and edgeR. The right panel shows the same data as the left panel but zooming in on the y-axis.

Figure 5 displays the evidence for differential transcript usage over that for differential gene expression, now using *DRIMSeq* and *edgeR*. One obvious contrast with Figure 4 is that *DRIMSeq* outputs lower non-zero adjusted p-values than *DEXSeq* does, where *DEXSeq* instead outputs 0 for many genes. The plots look more similar when zooming in on the *DRIMSeq* y-axis, as can be seen in the right panel of Figure 5.

bigpar()
plot(-log10(tt$FDR), -log10(res.sub$adj_pvalue), col=col,
     xlab="Gene expression",
     ylab="Transcript usage")
legend("topright",
        c("DGE","DTE","DTU","null"),
        col=c(1:3,8), pch=20, bty="n")

bigpar()
plot(-log10(tt$FDR), -log10(res.sub$adj_pvalue), col=col,
     xlab="Gene expression",
     ylab="Transcript usage", ylim=c(0,20))
legend("topright",
        c("DGE","DTE","DTU","null"),
        col=c(1:3,8), pch=20, bty="n")

### End of workflow section

This marks the end of the *workflow* section of the article. The following sections provide an *evaluation* of the methods presented in this workflow for DTU and DGE, alongside evaluation of other popular methods for DTU and for DGE. We additionally provide an evaluation of popular methods for DTE. While the workflow does not contain any code for performing DTE, we felt it was valuable to include an evaluation at this level of analysis as well. In practice, for count-based methods such as *DESeq2* and *edgeR*, performing DTE uses the same code as for DGE, but the counts are provided at the transcript level rather than summarized to the gene level. All of the analysis code used in the evaluations is provided in the associated GitHub repository ^25^.

## Evaluation

We investigated the performance of the Bioconductor packages used in the workflow above, *DRIMSeq* and *DEXSeq* for DTU, *DESeq2* and *edgeR* for DGE, relative to other popular methods for DTU and DGE. It is useful to assess the performance of methods for DGE in a simulation which also includes DTU – to see whether there is potentially an enrichment of false positives for certain types of genes according to the simulation. We also considered the question of DTE, and evaluated a number of methods designed for DGE – as well as methods designed for either DGE or DTE – by testing at the transcript level. DTE is not one of the analyses included in the workflow, but it is straightforward to perform with many of the DGE methods as well as with the methods explicitly designed to perform DGE or DTE.

As in the last plots presented in the workflow, in the evaluation we categorized genes by their simulation type, using the terms “DGE”, “DTE”, and “DTU”. When referring to the gene types in the simulation: these refer to the 10% of genes wherein all expressed transcripts had a constant fold change across condition (DGE), the 10% of genes where a single expressed transcript had a fold change across condition (DTE), and the 10% of genes where two transcripts had their expression switched across condition (DTU). Thus, the DTU genes counted as false positives for the DGE analysis, and vice versa. The DTE genes counted as true positives for the DGE analysis (because the total expression changed), and counted as true positives for DTU analysis if there were other expressed transcripts in the gene, or a false positive for DTU analysis if there were no other expressed transcripts (and so the proportions did not change).

We used three types of plots to explore the results. For assessing overall method performance for DTU, DGE, and DTE analysis, the *iCOBRA* package ^37^ was used to construct plots to assess the true positive rate (TPR) over the false discovery rate (FDR) at three nominal FDR thresholds: 1%, 5%, and 10%. We additionally used bar plots to show the number of false positives for each method across simulated gene-type categories (these plots referred to here as *breakdown plots*). We can do this at both the gene and transcript level: a false positive transcript can be categorized according to the type of gene to which it belongs. Finally, we created an *OFDR plot* for assessing the use of *stageR* for constructing gene-transcript OFDR sets, after applying *stageR* to the output of the DTU detection methods. The OFDR plot displays the observed OFDR on the x-axis and the sensitivity in recovering DTU transcripts on the y-axis. We used a fixed target OFDR for these plots of 5%. The code for evaluating all methods and constructing the *iCOBRA* plots is included in the simulation repository ^25^.

### Other popular methods for DTU

We assessed two other methods for DTU, *SUPPA2* ^38^ and *RATs* ^39^, both of which can take *Salmon* quantifications as input. For statistical testing of DTU, *SUPPA2* computes, for a given transcript, the difference in proportion across condition and the differences in proportion seen between biological replicates. *SUPPA2* then compares the difference in proportion across condition to the distribution of between-replicate differences for transcripts with similar average abundance by TPM. The transcript p-value is the tail probability from this empirical distribution, divided by two. *SUPPA2* is implemented as a command-line software package written in python, with a number of distinct features, including the ability to translate from *Salmon* transcript-level quantifications to individual splicing events, which are cataloged using a specific vocabulary described in the SUPPA2 software usage guide. *SUPPA2* additionally offers differential analysis on the splicing events, which may be more valuable to investigators than per-transcript results, depending on the research goals (similar to the exon-level primary use case of *DEXSeq*).

*RATs* uses a G-test of independence ^40^ at both the gene level and transcript level: at the gene level it compares the sets of abundances for each transcript across condition, and at the transcript level it compares the abundance of each transcript against the pooled abundance of the other transcripts in the gene, similar to the approach of *DEXSeq* in detecting differential exon usage, although with a different statistical test. *RATs* uses gene- and transcript-level expression filters before statistical testing. Unlike the other DTU methods discussed, *RATs* uses the inferential replicates (bootstrap or Gibbs samples) to repeat the testing multiple times, and then calculates the fraction of inferential replicates which achieve statistical significance. *RATs* also repeats the statistical testing multiple times using subsets of samples as a secondary assessment of reproducibility. The *RATs* software version we used additionally performs a filter on effect size, such that only genes or transcripts which were both reproducible according to inferential replicates and sub-sampling, and having a sufficiently large effect size are reported as DTU. *RATs* is implemented as an R package designed to detect DTU from transcript quantification as produced by *Salmon* or *kallisto* ^19^. As mentioned above, it can operate either on estimated counts alone, or on inferential replicates of the counts (bootstrap or Gibbs samples) as generated by either of these quantification tools. It is recommended in the *RATs* software guide to use a counts-from-abundance approach to generate the transcript counts.

We ran *SUPPA2* in its differential transcript usage mode. We enabled a filter to remove transcripts with less than 1 TPM. TPM filtering is a command-line option available during the diffSplice step of *SUPPA2* and this greatly improved the running time without loss of sensitivity (an additional filter to enable direct comparison with other methods is discussed below). We did not use the *SUPPA2* optional gene-correction, which does not correct for false discovery rate across genes, as we wanted to apply the aggregation and correction method perGeneQValue from *DEXSeq* to obtain an FDR-bounded set of genes and transcripts as output. We ran *RATs* with 30 bootstrap replicates from *Salmon*, generating counts from abundance by scaling up TPMs. The bootstrap replicates approach performed similarly to the approach without bootstrap replicates, with a minor improvement in the FDR and OFDR with including the bootstrap replicates. For easier visualization and to avoid overlapping data points, we only include the *RATs* bootstrap results in the evaluation plots.

To facilitate comparisons across methods, we only considered the genes and transcripts passing the *DRIMSeq* filters for minimum gene and transcript counts and minimum proportion. This eliminated genes which had expression too low to have very much statistical power for detecting DTU, and transcripts which were very lowly expressed in both conditions, and so not contributing useful information for DTU. We assessed that excluding these lowly expressed genes and transcripts did not change the relative differences in sensitivity of the methods, as they were not detectable by any of the methods with regards to DTU. For *SUPPA2*, we performed perGeneQValue only on those genes and transcripts passing the *DRIMSeq* filters. For *RATs*, we provided the bootstrap replicate counts-from-abundance for the genes and transcripts that passed the *DRIMSeq* filters. We performed identical stage-wise analysis with *stageR* on *SUPPA2* and *RATs* output, to allow direct comparison with *DRIMSeq* and *DEXSeq* stage-wise results and observed OFDR. Exact code for running *SUPPA2* and *RATs* is provided in the respective directories in the associated GitHub repository ^25^.

### DTU evaluation

In the workflow, we showed a typical analysis for a comparison of 6 vs 6 samples. As we were interested in the performance at various sample sizes, we performed the entire analysis for *DRIMSeq*, *DEXSeq*, *RATs*, and *SUPPA2* at per-group sample sizes of 3, 6, 9, and 12. The following evaluation corresponds to the “main” simulation as described in the Methods.

**Figure 6.**
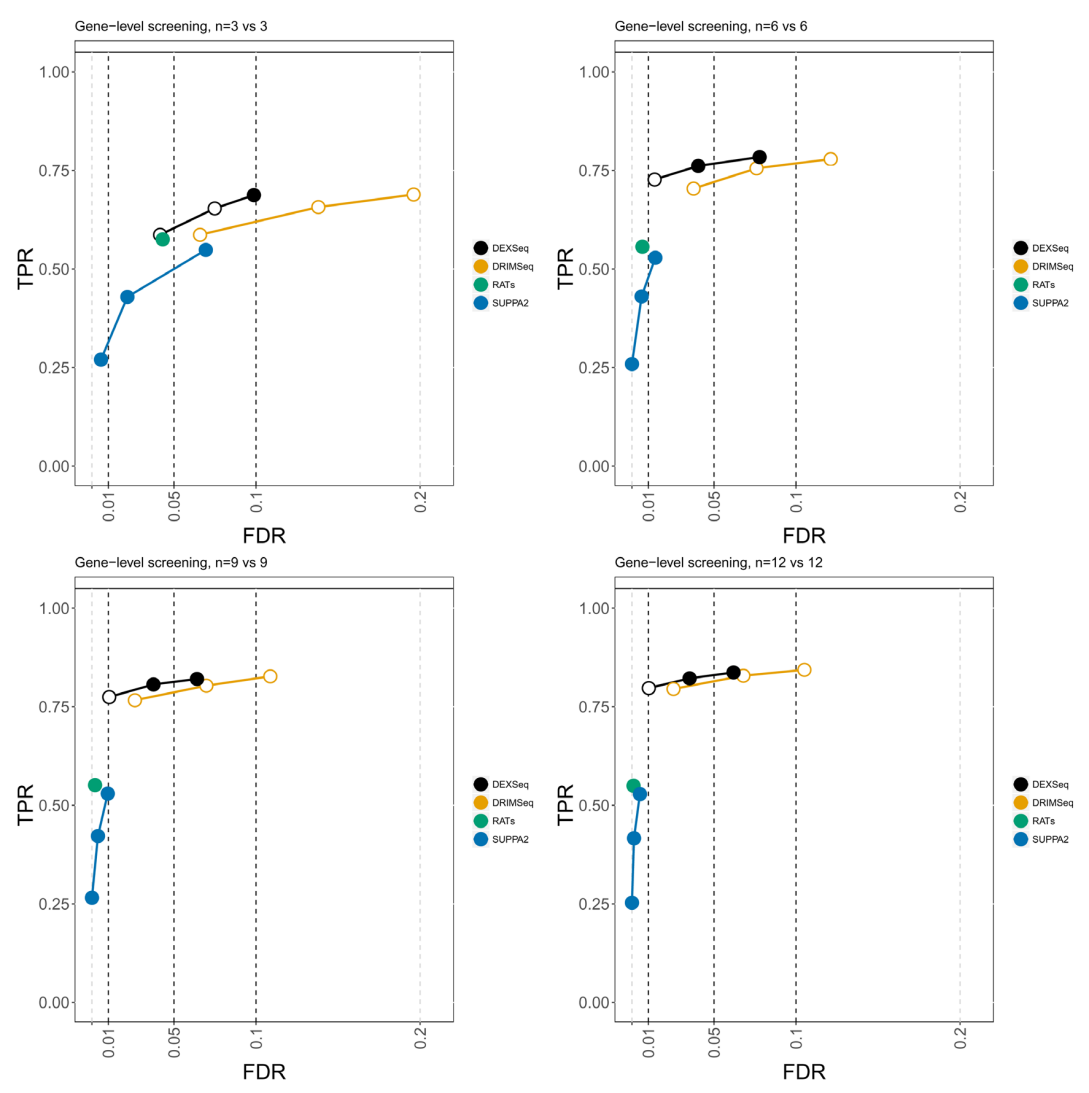
Gene-level screening for differential transcript usage (DTU). True positive rate (y-axis) over false discovery rate (FDR) (x-axis) for DEXSeq, DRIMSeq, RATs, and SUPPA2. The four panels are for per-group sample sizes: 3, 6, 9, and 12, as indicated in the title. Circles indicate thresholds of 1%, 5%, and 10% nominal FDR, which are filled if the observed value is less than the target (dashed vertical lines).

At the gene level, in terms of controlling the nominal FDR, *SUPPA2* always controlled its FDR, *RATs* controlled its FDR except for the 1% threshold for sample size 3, *DEXSeq* controlled its FDR except for the 1% threshold at all sample sizes and 5% threshold for sample size 3, and *DRIMSeq* exceeded its FDR but approached the target for larger sample sizes ( Figure 6). *RATs* gave nearly the same set of genes whether thresholding at 1%, 5%, or 10% nominal FDR, which we found was related to its default filtering procedures. Exceeding the nominal FDR level by a small amount should be considered with a method’s relative sensitivity in mind as well, compared to other methods. For example, for the 6 vs 6 comparison, *DRIMSeq* had an observed FDR of 12% at nominal 10%, meaning that for every 100 genes reported as containing DTU, the method reported 2 more false positive genes than its FDR target would allow. In general, *SUPPA2* and *RATs* were able to strictly control the FDR, while *DRIMSeq* and *DEXSeq* sometimes exceeded their FDR but with a large gain in sensitivity, particularly for per-group sample sizes of 6 or larger.

We further broke down the true positives and false positives at the gene level, for a target 5% FDR, according to the simulated gene type (“DGE”, “DTE”, “DTU”, or “null”). The true positives for most methods matched the gene-type proportion of true genes with transcript usage changes ( Supplementary Figure 2). About two-thirds were from the simulated DTU genes (two transcripts with swapped expression), and one-third were from simulated DTE genes with one transcript differentially expressed but where the proportions did change because at least one other transcript was expressed. *SUPPA2* and *RATs* had a slight decrease in relative sensitivity for the simulated DTE genes. The false positives for methods mostly tracked with the proportion of genes without transcript usage changes ( Supplementary Figure 3). The methods that tended to exceeded the target FDR, *DRIMSeq* and *DEXSeq*, did not have any particular category of simulated gene type that was over-represented in the false positives.

**Figure 7.**
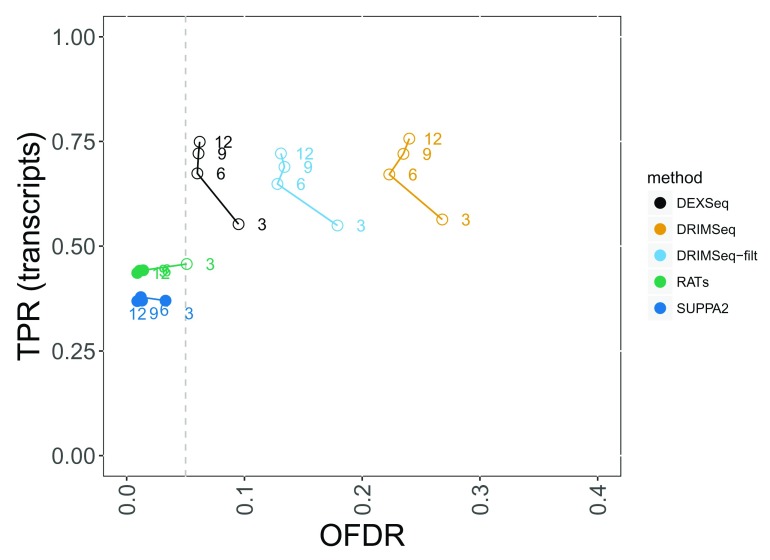
Number of true positives and observed overall false discovery rate (OFDR) using stageR for a 5% target. Each method is drawn as a line, and the numbers to the right of the points indicate the per-group sample size. Adjusted p-values for a nominal 5% OFDR (dashed vertical line) were generated for DEXSeq, DRIMSeq (with and without post-hoc filtering), RATs, and SUPPA2 from gene- and transcript-level p-values using the stageR framework for stage-wise testing.

We assessed the overall false discovery rate (OFDR) procedure implemented with *stageR* using gene- and transcript-level p-values from *DRIMSeq*, *DEXSeq*, *RATs*, and *SUPPA2*, for a 5% target OFDR. *SUPPA2* and *RATs* controlled the target OFDR at all sample sizes, with *RATs* having nearly exactly 5% OFDR at the smallest sample size. *DEXSeq* input to *stageR* was close to the 5% OFDR target except for a sample size of 3, which had an OFDR around 10%. For *DRIMSeq*, we assessed whether raising the p-values to 1 for transcripts with small proportion SD helped to recover OFDR control. The observed OFDR for *DRIMSeq* with proportion SD filtering was at lowest around 12% at per-group sample size of 6 and higher ( Figure 7). Without the filtering, the observed OFDR for *DRIMSeq* was otherwise around 25%. While *SUPPA2* and *RATs* always controlled the OFDR, we noted that the sensitivity in terms of transcripts detected via the *stageR* two-stage procedure did not increase with sample size, unlike *DRIMSeq* and *DEXSeq* which approached 75% sensitivity at the largest sample size.

**Figure 8.**
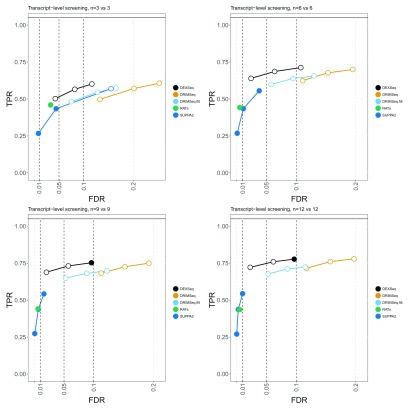
Transcript-level differential transcript usage (DTU) analysis without stage-wise testing. True positive rate (y-axis) over false discovery rate (x-axis) for DEXSeq, DRIMSeq (with and without post-hoc filtering), RATs, and SUPPA2. The four panels are for per-group sample sizes: 3, 6, 9, and 12, as indicated in the title. Circles indicate thresholds of 1%, 5%, and 10% nominal FDR.

Finally, although the workflow showed how to integrate the transcript- and gene-level tests using the *stageR* procedure, we also evaluated the transcript-level adjusted p-values alone for *DRIMSeq*, *DEXSeq*, *RATS*, and *SUPPA2*. This evaluation corresponds to an analysis which does not use any gene-level aggregation, and does not use *stageR*, but considers only the adjusted p-values per transcript from each method. Here we computed the standard FDR, where the unit of false discovery is the *transcript*, in contrast to the OFDR where the unit of false discovery is the *gene*. *SUPPA2* and *RATs* tended to control their FDR as in the gene-level analysis ( Figure 8). *DEXSeq* only slightly exceeded its FDR for sample sizes 6 or larger, eventually controlling the 10% target FDR. *DRIMSeq* with proportion SD filtering approached the target FDR as sample size increased for the 5% and 10% targets, while without filtering, the observed FDR was much higher than the target.

The breakdown of false positives by gene type, for a target 5% FDR, was revealing for the transcript-level analysis, as we noticed that for this simulation *DRIMSeq* tended to have an excess of false positive transcripts that belonged to true DTU genes ( Supplementary Figure 4), relative to what would be expected by random sampling of the transcripts not participating in differential transcript usage. From looking at individual examples, we noticed that *DRIMSeq* would sometimes correctly identify the gene as DTU, but have a low p-value for one or more additional transcripts beyond the two transcripts whose expression was actually swapped. This excess of false positive transcripts from true DTU genes was also observed for *DEXSeq* as sample size increased.

We also assessed all of the above metrics for a sample size of 2 vs 2, including gene-level DTU detection, OFDR, and transcript-level DTU detection ( Supplementary Figure 5). This additional analysis at a very low per-group sample size revealed that most of the methods could not control the gene-level FDR, only *RATs* was able to control a target 10% FDR. *SUPPA2* and *RATs* were the closest at controlling the target 5% OFDR, with observed OFDR around 10%. At the transcript-level, only *RATs* could control the 10% FDR, and with less than 50% sensitivity. This analysis revealed that a per-group sample size of 2 is probably not sufficient to to detect most DTU genes and transcripts.

In Table 1 we include the compute time for each method at various sample sizes. Compute time includes only the call_DTU step of *RATs*, and only the diffSplice step of *SUPPA2* (the other *SUPPA2* steps take less than a minute). For *DRIMSeq* and *DEXSeq*, we include the compute time of the estimation steps (importing counts with *tximport* and filtering takes only a few seconds).

**Table 1.** Compute time of methods for differential transcript usage (DTU) in hours:minutes by per-group sample size, using one core. The fourth row, RATs (count), gives the compute time using the scaledTPM counts, and not the bootstrap replicates.

Method	n=3	n=6	n=9	n=12
*DRIMSeq*	0:15	0:15	0:18	0:18
*DEXSeq*	0:01	0:02	0:05	0:10
*RATs*	1:41	2:34	4:44	6:08
*RATs (count)*	0:10	0:38	1:22	2:32
*SUPPA2*	0:16	1:18	3:48	5:33

### Fixed per-gene dispersion

In order to further investigate performance differences between the two methods highlighted in the workflow section, *DRIMSeq* and *DEXSeq*, we generated an additional simulation we called “fixed per-gene dispersion” in which genes were assigned Negative Binomial dispersion parameters by matching the gene-level count to the joint distribution of mean and dispersions on the GEUVADIS dataset. Then transcript-level counts were generated with all transcripts of a gene being assigned the same Negative Binomial dispersion parameter. This contrasts with the “main” simulation, in which each transcript was assigned its own dispersion parameter, resulting in heterogeneity of dispersion within a gene. As we do not know the degree to which transcripts of a gene would have correlated biological variability in an experimental dataset, we also include the results for the count-based methods that estimate precision/dispersion, *DRIMSeq* and *DEXSeq*, for this additional simulation.

**Figure 9.**
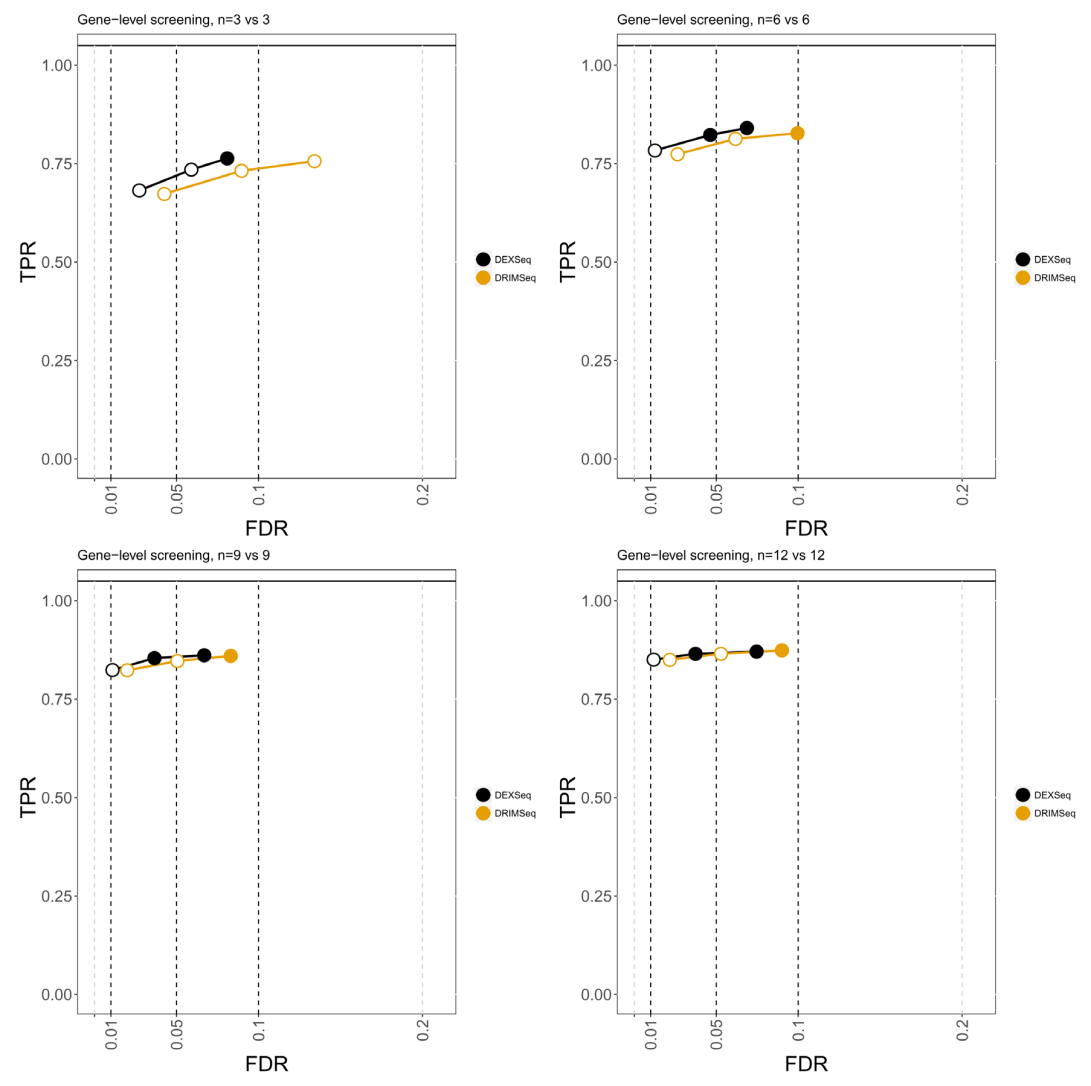
Gene-level screening for differential transcript usage (DTU), on the “fixed per-gene dispersions” simulation. The four panels are for per-group sample sizes: 3, 6, 9, and 12, as indicated in the title. Circles indicate thresholds of 1%, 5%, and 10% nominal FDR.

**Figure 10.**
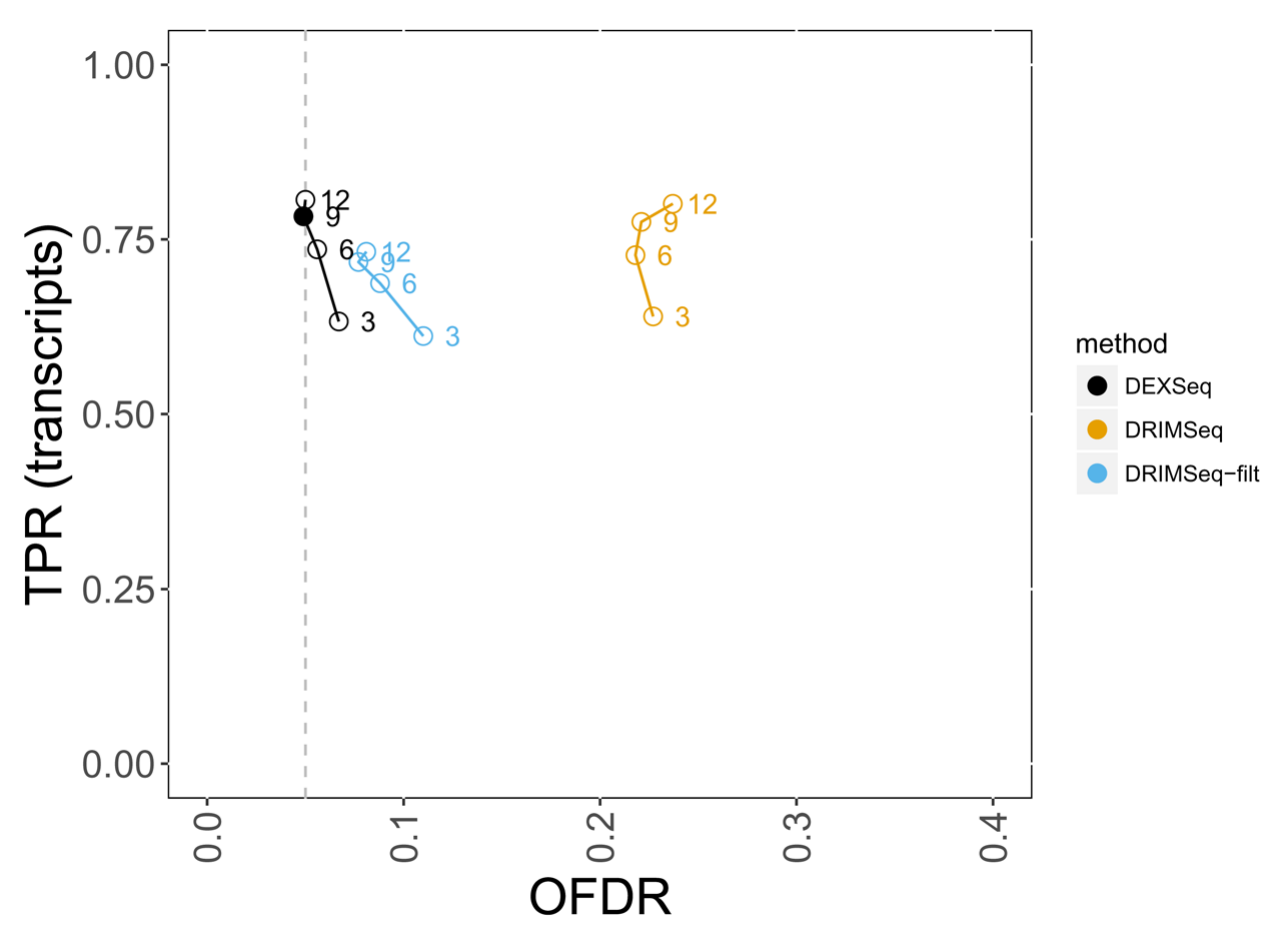
Number of true positives and observed overall false discovery rate (OFDR) using stageR for 5% target, on the “fixed per-gene dispersions” simulation.

**Figure 11.**
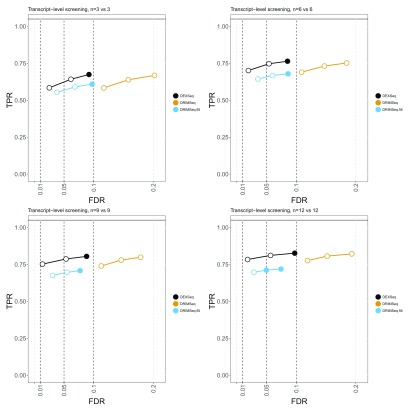
Transcript-level differential transcript usage (DTU) analysis without stage-wise testing, on the “fixed per-gene dispersions” simulation. The four panels are for per-group sample sizes: 3, 6, 9, and 12, as indicated in the title. Circles indicate thresholds of 1%, 5%, and 10% nominal FDR.

*DRIMSeq*, which estimates a single precision parameter per gene, performed better in terms of FDR control on this simulation at the gene level ( Figure 9), although we note that *DRIMSeq* nearly controlled FDR at the gene level already in the first simulation for samples sizes 6 and larger. *DEXSeq* models different dispersion parameters for every transcript, and its performance changes less across the two simulations, although it had better OFDR and FDR control for the smallest sample size. *DRIMSeq* with proportion SD filtering had much better control of OFDR ( Figure 10) and of FDR in the transcript-level analysis ( Figure 11) in the “fixed per-gene dispersion” simulation compared to the “main” simulation. We also assessed the true positive and false positive proportions for the gene level and transcript level for the “fixed per-gene dispersion” simulation, and these were very similar to the true positive and false positive breakdown plots generated for the “main” simulation (data not shown).

### Negative binomial gene-level counts

We additionally compared *DRIMSeq* and *DEXSeq* on an existing human transcriptome simulation dataset generated by Soneson *et al*. ^33^ and analyzed in the *DRIMSeq* publication ^11^. This simulation has similarities to the “fixed per-gene dispersion” simulation in that gene-level estimated mean and dispersion parameters from real datasets were used. However, instead of generating transcript-level counts from a Negative Binomial distribution, the Soneson *et al*. ^33^ simulation generated gene-level counts, converted these to an abundance measure, and then used a Dirichlet distribution to generate random proportion vectors per sample to distribute the abundances to transcript isoforms. To simulate DTU, 1,000 genes were selected and the abundance of the two most abundant transcripts was swapped. Finally RSEM-sim was used to generate reads ^41^. We used the identical *kallisto* ^19^ estimated transcript counts generated by Soneson *et al*. ^33^ and assessed performance via the true DTU status per gene published as supporting data.

**Figure 12.**
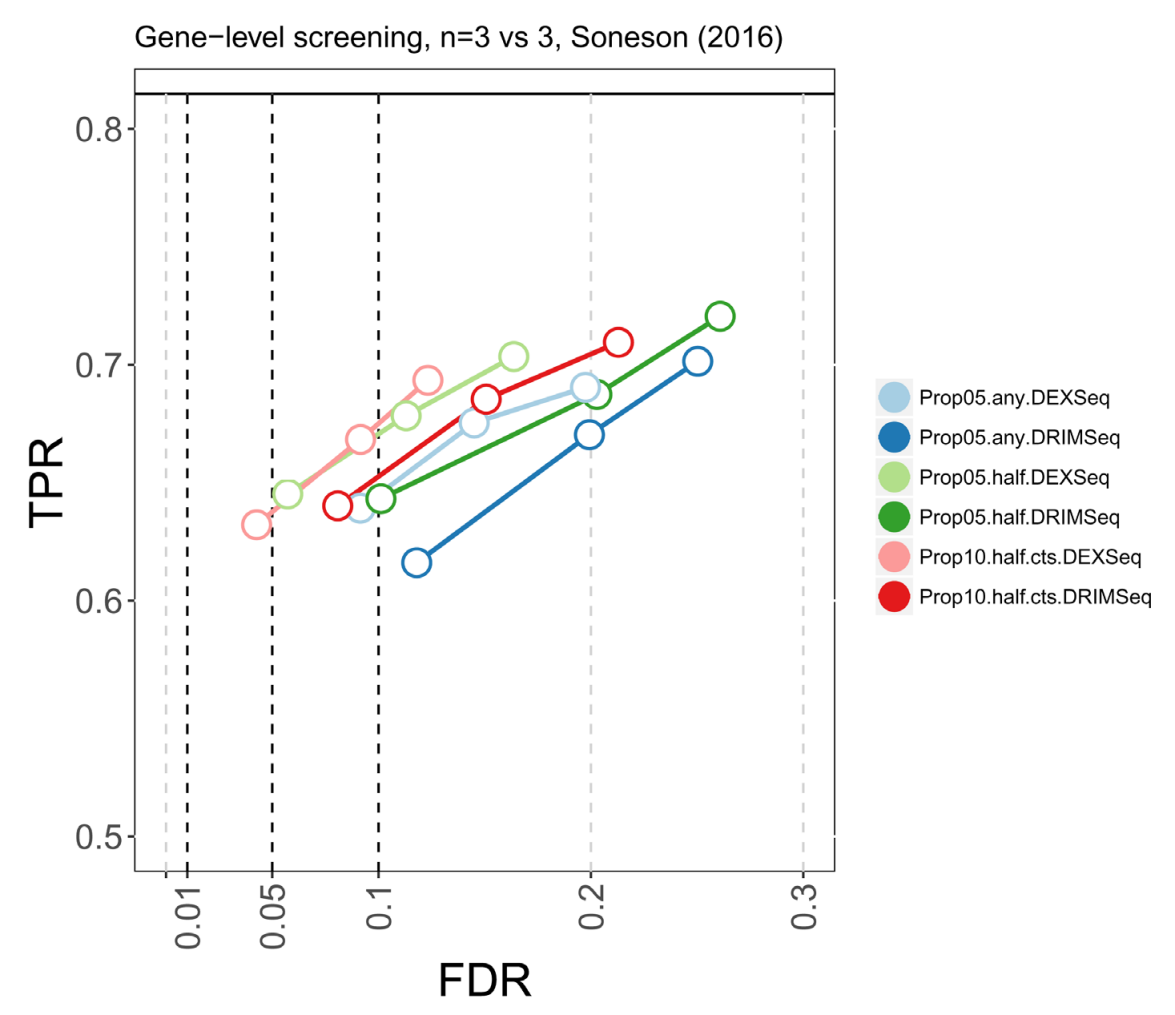
Gene-level screening for differential transcript usage (DTU) analysis on the 3 vs 3 human transcriptome simulated data from Soneson *et al*. ^33^. In the method names, “Prop05.any” refers to proportion filtering such that a single sample must have a proportion higher than 5% for a transcript to be kept. “Prop05.half” refers to proportion filtering such that 3 out of 6 samples must have a proportion higher than 5%. “Prop10.half.cts” refers to the same filters recommended in this workflow: 3 of out 6 samples with proportions higher than 10% to keep a transcript, 3 out of 6 samples with transcript counts greater than 10 to keep a transcript, and all samples with gene counts greater than 10 to keep a gene. Circles indicate thresholds of 1%, 5%, and 10% nominal FDR. Current release versions of methods were used, though results were very similar for “Prop05.any” for DEXSeq version 1.10.1 used in Nowicka and Robinson ^11^.

As reported in previous publications, we found that both *DRIMSeq* and *DEXSeq* had better control of FDR with increased filtering ( Figure 12). The best performance of both methods was observed with the gene-level and transcript-level count filters, and a sample-sized-based proportion filter of 0.1, as recommended in this workflow. The sensitivity (TPR) around 70% is similar to that reported by Nowicka and Robinson ^11^, and similar to what we observed in our “main” and “fixed per-gene dispersion” simulations. We recreated the “5%-any” filtering rule from Nowicka and Robinson ^11^, which kept a transcript if it was observed with a proportion higher than 5% in *any* of the samples. This is in contrast with the recommendation from this workflow and the current *DRIMSeq* vignette which makes use of the *number of replicates per condition* for the transcript-level filters, i.e. requiring 3 out of 6 samples to have proportions higher than a certain threshold for a 3 vs 3 experiment. For the “5%-any” filtering, for target 10% FDR, we observed FDR for *DRIMSeq* and *DEXSeq* at around 25% and 20%, respectively. This is not identical, but comparable to the *∼* 28% FDR for both methods reported by Nowicka and Robinson ^11^.

Again, a caveat of all of our comparative evaluations of *DRIMSeq* and *DEXSeq* is that we do not know whether various real RNA-seq experiments will more closely reflect heterogeneous dispersion or fixed dispersion within genes, or if the counts within a gene are better modeled by distributing gene-level abundance to transcripts via a Dirichlet distribution as in Soneson *et al*. ^33^. However, we have examined simulations reflecting each of these cases, and confirmed that minimum count and minimum proportion filtering benefit both *DRIMSeq* and *DEXSeq* in terms of their FDR and OFDR control.

### Methods for DGE and DTE

In the workflow, we showed how *DESeq2* and *edgeR* can be used to detect differential gene expression with *Salmon* quantifications imported and summarized to the gene level via *tximport*. There are many other methods for testing for DGE. Here we will briefly review some of the methods with well-documented R packages hosted on Bioconductor, CRAN, or GitHub and then compare their performance in detecting DGE and DTE on the “main” simulation. The primary reasons for including this DGE and DTE assessment is that we are interested in how the tools designed for DGE perform when DTU is present, and we are also interested in assessing how the DGE methods, some of which were not designed for DTE, perform when provided with estimated transcript counts.

A number of the methods, *edgeR* ^7^, *edgeR-QL* (using the quasi-likelihood functions) ^42^, *EBSeq* ^43^, and *DESeq2* ^20^, use a Negative Binomial distribution to model the counts, and empirical Bayes techniques to estimate per-gene parameters despite limited sample size. The Negative Binomial is a useful distribution for counts, in that it has a parameter for the location of the mean count, as well as a *dispersion* parameter for the expected spread of counts. For high counts, the dispersion parameter is approximately equal to the square of the coefficient of variation (the standard deviation over the mean), and so can be interpreted for high counts as how much the data can be expected to vary around the mean count, relative to the size of the mean.

*EBSeq*, uniquely among these Negative-Binomial-based models, was also specifically designed to accommodate extra uncertainty in transcript counts when assessing DTE. *EBSeq* has a DTE mode in which the number of transcript isoforms per gene is supplied as a piece of information before running the main analysis function. *edgeR-QL* differs from *edgeR* and *DESeq2* in that it accounts for uncertainty in the dispersion estimate via a quasi-likelihood framework. *limma* with *voom* transformation ^10^ and *sleuth* ^44^ model the log of scaled counts, with *sleuth* additionally taking into account inferential variance on the transcript- and gene-level counts, unlike any of the other DGE or DTE methods we assessed. Finally, *SAMseq* ^45^ scales counts via a resampling approach and applies rank-based statistical tests to detect differences in samples across condition; by operating on ranks, it is much less sensitive to count outliers or in general to mis-specified parametric modeling.

For DGE and DTE, the following filtering functions or rules for each package were used: filterByExpr for *edgeR*, *edgeR-QL*, and *limma* with *voom*, sleuth_prep for *sleuth*, and a custom filter requiring a count of 10 or more for half the samples for *DESeq2*, *EBSeq*, and *SAMSeq*, which do not come with their own filtering functions. For evaluation, all genes (or transcripts in DTE analysis) were included, except those for which no software provided an adjusted p-value.

### DGE evaluation

We assessed the aforementioned R packages for differential gene expression, to determine true positive rate and control of false discovery rate on the “main” simulated dataset. In this analysis, the simulated “DGE” genes (where all transcripts are differentially expressed at the same fold change), and the “DTE” genes (where a single transcript was chosen to be differentially expressed) should count as true positives for differential gene expression, while the simulated “DTU” genes should count as false positives for differential gene expression, as the total expression of the gene remains constant.

We compared *DESeq2*, *EBSeq*, *edgeR*, *limma* with *voom* transformation, *SAMseq*, and *sleuth*. We used *tximport* to summarize *Salmon* abundances to the gene level, and provided all methods other than *DESeq2* and *sleuth* with the lengthScaledTPM count matrix. *sleuth* takes as input the quantification from *kallisto* ^19^, which was run with 30 bootstrap samples and bias correction. For gene-level analysis in *sleuth*, the argument aggregation_column="gene_id" was used. As *DESeq2* has specially designed import functions for taking in estimated gene counts and an offset from *tximport*, we used this approach to provide *Salmon* summarized gene-level counts and an offset. *edgeR* and *edgeR-QL* had the same performance using the counts and offset approach or the lengthScaledTPM approach, so we used the latter for code simplicity. The exact code used to run the different methods can be found at the simulation code repository ^25^. Compute time for the different gene-level methods are presented in Table 2.

**Table 2.** Compute time of methods for differential gene expression (DGE) rounded to the minute by per-group sample size. Compute time includes data import and summarization to gene-level quantities using one core.

Method	n=3	n=6	n=9	n=12
*DESeq2*	<1	<1	<1	<1
*EBSeq*	1	2	2	3
*edgeR*	<1	<1	<1	<1
*edgeR-QL*	<1	<1	<1	<1
*limma*	<1	<1	<1	<1
*SAMseq*	<1	<1	<1	<1
*sleuth*	2	4	5	7

**Figure 13.**
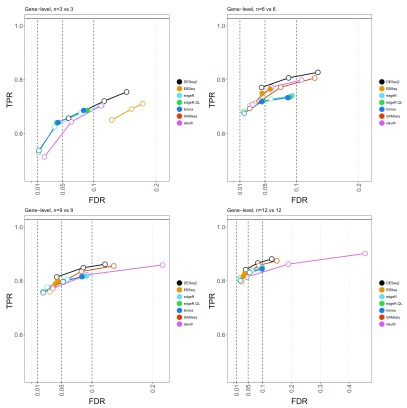
True positive rate over false discovery rate for differential gene expression of the simulated dataset. The four panels are for per-group sample sizes: 3, 6, 9, and 12, as indicated in the title. The y-axis remains fixed but the x-axis changes scale in the bottom panels.

*iCOBRA* plots with true positive rate over false discovery rate for gene-level analysis across four different pergroup sample sizes are presented in Figure 13. For the smallest per-group sample size of 3, all methods except *DESeq2* and *EBSeq* tended to control the FDR, while those two methods had, for example, 15% and 18% FDR respectively at the nominal 10% rate. *SAMseq*, with so few samples, did not have adequate sensitivity to detect DGE. At the per-group sample size of 6, all methods except *DESeq2* and *SAMseq* tended to control the FDR. At this sample size, *EBSeq* controlled its FDR. For the largest per-group sample sizes, 9 and 12, the performance of many methods remained similar as previously, except *sleuth* did not control its nominal FDR. For ease of comparison, we also provide Supplementary Figure 6 where the x-axis remains fixed through the sample sizes. We performed an additional simulation, called “uniform coverage”, to see if the performance of *sleuth* at higher sample sizes was related to the realistic GC bias parameters used in the simulation, but simulating fragments uniformly from the transcripts revealed the same performance at per-group sample sizes 9 and 12 ( Supplementary Figure 7). We then performed another simulation, called “low DE”, wherein we reduced the number of DGE, DTE and DTU genes from 10% to 5% each. In the “low DE” simulation, *sleuth* did recover control of the FDR at the nominal 5% and 10% FDR ( Supplementary Figure 8).

As in the DTU evaluation, for the DGE evaluation we broke down the number of false positives by simulated gene type, for a target 5% FDR ( Supplementary Figure 9). Here there was a slight increase of “DTU” gene types in the gene-level false positives for all methods, relative to what would be expected by random sampling of the genes without differential gene expression.

### DTE evaluation

Finally, we assessed the Bioconductor and R packages used in the DGE evaluation for differential transcript expression analysis. While we believe the separation of differential transcript usage and differential gene expression described in the workflow represents an easily interpretable approach, some investigators may prefer to assess differential expression on a per-transcript basis. For this assessment, all of the simulated non-null transcripts count as true positives of differential transcript expression, whether they originate from the simulated “DGE”, “DTE”, or “DTU” genes. For most of the methods, we simply provided the transcript-level data to the same functions as for the DGE analysis. *EBSeq* was provided with the number of isoforms per gene. The compute time of the methods is presented in Table 3.

**Table 3.** Compute time of methods for differential transcript expression (DTE) rounded to the nearest minute by per-group sample size. Compute time includes data import.

Method	n=3	n=6	n=9	n=12
*DESeq2*	<1	<1	<1	1
*EBSeq*	5	11	18	22
*edgeR*	<1	<1	<1	<1
*edgeR-QL*	<1	<1	<1	<1
*limma*	<1	<1	<1	<1
*SAMseq*	<1	<1	<1	1
*sleuth*	2	2	2	2

**Figure 14.**
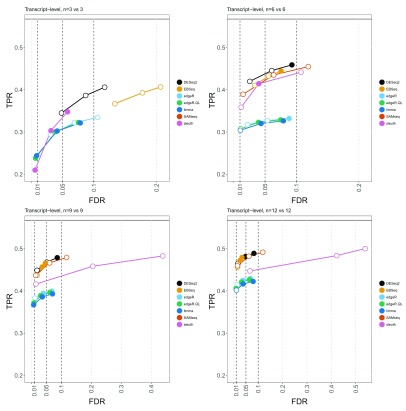
True positive rate over false discovery rate for differential transcript expression of the simulated dataset. The four panels are for per-group sample sizes: 3, 6, 9, and 12, as indicated in the title. The y-axis remains fixed but the x-axis changes scale in the bottom panels.

*iCOBRA* plots with the true positive rate over false discovery rate for the transcript-level analysis are shown in Figure 14. The performance at per-group sample size of 3 was similar to the gene-level analysis, except *DESeq2* came closer to controlling the FDR and *EBSeq* performed slightly worse than before, while the rest of the methods tended to control their FDR. At per-group sample size of 6, all of the evaluated methods tended to control the FDR, though *DESeq2*, *EBSeq*, *SAMseq*, and *sleuth* tended to have higher sensitivity than *edgeR*, *edgeR-QL* and *limma*. The same issue of FDR control for *sleuth* was seen in the transcript-level analysis as in the gene-level analysis, for per-group sample size 9 and 12. For ease of comparison, we also provide Supplementary Figure 10 where the x-axis remains fixed through the sample sizes. We broke down the number of false positives at the transcript level by gene type, for a target 5% FDR ( Supplementary Figure 11). All methods had proportion of false positives similar to what would be expected by random sampling of the non-differentially expressed transcripts.

## Discussion

Here we presented a workflow for analyzing RNA-seq experiments for differential transcript usage across groups of samples. The Bioconductor packages used, *DRIMSeq*, *DEXSeq*, and *stageR*, are simple to use and fast when run on transcript-level data. We show how these can be used downstream of transcript abundance quantification with *Salmon*. We evaluated these methods on a simulated dataset and showed how the transcript usage results complement a gene-level analysis, which can also be run on output from *Salmon*, using the *tximport* package to aggregate quantifications to the gene level. We used the simulated dataset to evaluate Bioconductor and other R packages for differential gene expression, and differential transcript expression.

From the DTU evaluations, we found that *SUPPA2* and *RATs* tended to always control the FDR, at the cost of reduced sensitivity relative to *DRIMSeq* and *DEXSeq* especially as the per-group sample size increased to 6 and higher. *DEXSeq* with minimum transcript- and gene-level count filters, and 10% minimum proportion filter tended to have good control of a target 10% FDR for sample sizes of 6 and higher. *DRIMSeq* with those three filters and post-hoc proportion SD filtering approached control of a target 10% FDR. Both of these methods had increased sensitivity as the sample size increased. Both of these methods make use of linear models and R’s built-in design formula, and so can be extended to complex designs, including within-individual comparisons, blocking for batch effects, or additional interaction terms.

Although statistical power depends obviously on biological variability and on the effect size (amount of change in proportion across conditions), from this simulation study we would recommend per-group sample sizes larger than 3 to achieve greater than 50% sensitivity for detecting DTU. The maximal gene-level DTU sensitivity achieved in the “main” simulation was around 80% at a per-group sample size of 12. This reflects the fact that for some of the DTU genes, the change in proportions across conditions was small, and was not detectable relative to the within-condition biological variability. As the “main” simulation used *gene-level* mean and dispersion estimates from real data to generate transcript-level counts, it is possible that RNA-seq datasets may exhibit even more biological variability on the transcript counts than seen here, thus underscoring the need for sufficient sample size to achieve a reasonably high sensitivity for detecting DTU.

We recommend the use of *stageR* in DTU analysis for its use of a formal statistical procedure involving a screening and confirmation stage, as this fits closely to what we expect a typical analysis to entail. It is likely that an investigator would want both a list of statistically significant genes *and* transcripts participating in DTU, and *stageR* provides error control on this pair of lists, assuming that the underlying tests are well calibrated.

From the DGE and DTE analyses of this particular simulation data, we found that *edgeR* had better control of FDR than *DESeq2*. *DESeq2* approached its target FDR as sample size grew. Popular methods that had relatively high sensitivity and control of FDR across all sample sizes include *limma* with *voom* transformation and *edgeR-QL*, both of which had better control than *edgeR* at per-group sample size of 3.

One potential limitation of this workflow is that, in contrast to other methods such as the standard *DEXSeq* analysis, *SUPPA2*, or *LeafCutter* ^46^, here we considered and detected expression switching between annotated transcripts. Other methods such as *DEXSeq* (exon-based), *SUPPA2*, or *LeafCutter* may benefit in terms of power and interpretability from performing statistical analysis directly on exon usage or splice events. Methods such as *DEXSeq* (exon-based) and *LeafCutter* benefit in the ability to detect un-annotated events. The workflow presented here would require further processing to attribute transcript usage changes to specific splice events, and is limited to considering the estimated abundance of annotated transcripts.

## Session information

The following provides the session information used when compiling this document.

devtools::session_info()

## Session info -------------------------------------------------------------

##  setting  value
##  version  R version 3.5.0 (2018-04-23)
##  system   x86_64, darwin15.6.0
##  ui       X11
##  language (EN)
##  collate  en_US.UTF-8
##  tz       America/New_York
##  date     2018-06-17

## Packages -----------------------------------------------------------------

##  package              * version   date       source
##  acepack                1.4.1     2016-10-29 CRAN (R 3.5.0)
##  annotate               1.58.0    2018-05-01 Bioconductor
##  AnnotationDbi        * 1.42.1    2018-05-08 Bioconductor
##  assertthat             0.2.0     2017-04-11 CRAN (R 3.5.0)
##  backports              1.1.2     2017-12-13 cran (@1.1.2)
##  base                 * 3.5.0     2018-04-24 local
##  base64enc              0.1-3     2015-07-28 CRAN (R 3.5.0)
##  Biobase              * 2.40.0    2018-05-01 Bioconductor
##  BiocGenerics         * 0.26.0    2018-05-01 Bioconductor
##  BiocInstaller        * 1.30.0    2018-05-04 Bioconductor
##  BiocParallel         * 1.14.1    2018-05-06 Bioconductor
##  BiocStyle              2.8.0     2018-05-01 Bioconductor
##  BiocWorkflowTools      1.6.1     2018-05-24 Bioconductor
##  biomaRt                2.36.0    2018-05-01 Bioconductor
##  Biostrings             2.48.0    2018-05-01 Bioconductor
##  bit                    1.1-12    2014-04-09 CRAN (R 3.5.0)
##  bit64                  0.9-7     2017-05-08 CRAN (R 3.5.0)
##  bitops                 1.0-6     2013-08-17 CRAN (R 3.5.0)
##  blob                   1.1.1     2018-03-25 CRAN (R 3.5.0)
##  bookdown               0.7       2018-02-18 CRAN (R 3.5.0)
##  checkmate              1.8.5     2017-10-24 CRAN (R 3.5.0)
##  cluster                2.0.7-1   2018-04-13 CRAN (R 3.5.0)
##  codetools              0.2-15    2016-10-05 CRAN (R 3.5.0)
##  colorspace             1.3-2     2016-12-14 CRAN (R 3.5.0)
##  compiler               3.5.0     2018-04-24 local
##  data.table             1.11.2    2018-05-08 CRAN (R 3.5.0)
##  datasets             * 3.5.0     2018-04-24 local
##  DBI                    1.0.0     2018-05-02 CRAN (R 3.5.0)
##  DelayedArray         * 0.6.0     2018-05-01 Bioconductor
##  DESeq2               * 1.20.0    2018-05-01 Bioconductor
##  devtools             * 1.13.5    2018-02-18 CRAN (R 3.5.0)
##  DEXSeq               * 1.26.0    2018-05-01 Bioconductor
##  digest                 0.6.15    2018-01-28 cran (@0.6.15)
##  DRIMSeq              * 1.8.0     2018-05-01 Bioconductor
##  edgeR                * 3.22.2    2018-05-24 cran (@3.22.2)
##  evaluate               0.10.1    2017-06-24 CRAN (R 3.5.0)
##  foreign                0.8-70    2017-11-28 CRAN (R 3.5.0)
##  Formula                1.2-3     2018-05-03 CRAN (R 3.5.0)
##  genefilter             1.62.0    2018-05-01 Bioconductor
##  geneplotter            1.58.0    2018-05-01 Bioconductor
##  GenomeInfoDb         * 1.16.0    2018-05-01 Bioconductor
##  GenomeInfoDbData       1.1.0     2018-01-10 Bioconductor
##  GenomicRanges        * 1.32.2    2018-05-06 Bioconductor
##  ggplot2                2.2.1     2016-12-30 CRAN (R 3.5.0)
##  git2r                  0.21.0    2018-01-04 CRAN (R 3.5.0)
##  graphics             * 3.5.0     2018-04-24 local
##  grDevices            * 3.5.0     2018-04-24 local
##  grid                   3.5.0     2018-04-24 local
##  gridExtra              2.3       2017-09-09 CRAN (R 3.5.0)
##  gtable                 0.2.0     2016-02-26 CRAN (R 3.5.0)
##  Hmisc                  4.1-1     2018-01-03 CRAN (R 3.5.0)
##  htmlTable              1.11.2    2018-01-20 CRAN (R 3.5.0)
##  htmltools              0.3.6     2017-04-28 CRAN (R 3.5.0)
##  htmlwidgets            1.2       2018-04-19 CRAN (R 3.5.0)
##  httr                   1.3.1     2017-08-20 CRAN (R 3.5.0)
##  hwriter                1.3.2     2014-09-10 CRAN (R 3.5.0)
##  IRanges              * 2.14.9    2018-05-15 Bioconductor
##  knitr                * 1.20      2018-02-20 CRAN (R 3.5.0)
##  labeling               0.3       2014-08-23 CRAN (R 3.5.0)
##  lattice                0.20-35   2017-03-25 CRAN (R 3.5.0)
##  latticeExtra           0.6-28    2016-02-09 CRAN (R 3.5.0)
##  lazyeval               0.2.1     2017-10-29 CRAN (R 3.5.0)
##  limma                * 3.36.1    2018-05-05 Bioconductor
##  locfit                 1.5-9.1   2013-04-20 CRAN (R 3.5.0)
##  magrittr               1.5       2014-11-22 CRAN (R 3.5.0)
##  Matrix                 1.2-14    2018-04-13 CRAN (R 3.5.0)
##  matrixStats          * 0.53.1    2018-02-11 CRAN (R 3.5.0)
##  memoise                1.1.0     2017-04-21 CRAN (R 3.5.0)
##  methods              * 3.5.0     2018-04-24 local
##  munsell                0.4.3     2016-02-13 CRAN (R 3.5.0)
##  nnet                   7.3-12    2016-02-02 CRAN (R 3.5.0)
##  parallel             * 3.5.0     2018-04-24 local
##  pillar                 1.2.2     2018-04-26 CRAN (R 3.5.0)
##  plyr                   1.8.4     2016-06-08 CRAN (R 3.5.0)
##  prettyunits            1.0.2     2015-07-13 CRAN (R 3.5.0)
##  progress               1.1.2     2016-12-14 CRAN (R 3.5.0)
##  R6                     2.2.2     2017-06-17 CRAN (R 3.5.0)
##  rafalib              * 1.0.0     2015-08-09 CRAN (R 3.5.0)
##  RColorBrewer         * 1.1-2     2014-12-07 CRAN (R 3.5.0)
##  Rcpp                   0.12.17   2018-05-18 cran (@0.12.17)
##  RCurl                  1.95-4.10 2018-01-04 CRAN (R 3.5.0)
##  reshape2               1.4.3     2017-12-11 CRAN (R 3.5.0)
##  rlang                  0.2.1     2018-05-30 cran (@0.2.1)
##  rmarkdown            * 1.9       2018-03-01 CRAN (R 3.5.0)
##  rnaseqDTU            * 0.1.0     2018-06-18 local (mikelove/rnaseqDTU@NA)
##  rpart                  4.1-13    2018-02-23 CRAN (R 3.5.0)
##  rprojroot              1.3-2     2018-01-03 cran (@1.3-2)
##  Rsamtools              1.32.0    2018-05-01 Bioconductor
##  RSQLite                2.1.1     2018-05-06 CRAN (R 3.5.0)
##  rstudioapi             0.7       2017-09-07 CRAN (R 3.5.0)
##  S4Vectors            * 0.18.1    2018-05-02 Bioconductor
##  scales                 0.5.0     2017-08-24 CRAN (R 3.5.0)
##  splines                3.5.0     2018-04-24 local
##  stageR               * 1.2.22    2018-06-14 cran (@1.2.22)
##  statmod                1.4.30    2017-06-18 CRAN (R 3.5.0)
##  stats                * 3.5.0     2018-04-24 local
##  stats4               * 3.5.0     2018-04-24 local
##  stringi                1.2.2     2018-05-02 CRAN (R 3.5.0)
##  stringr                1.3.1     2018-05-10 CRAN (R 3.5.0)
##  SummarizedExperiment * 1.10.1    2018-05-11 Bioconductor
##  survival               2.42-3    2018-04-16 CRAN (R 3.5.0)
##  tibble                 1.4.2     2018-01-22 CRAN (R 3.5.0)
##  tinytex                0.5       2018-04-16 CRAN (R 3.5.0)
##  tools                  3.5.0     2018-04-24 local
##  utils                * 3.5.0     2018-04-24 local
##  withr                  2.1.2     2018-03-15 CRAN (R 3.5.0)
##  xfun                   0.1       2018-01-22 CRAN (R 3.5.0)
##  XML                    3.98-1.11 2018-04-16 CRAN (R 3.5.0)
##  xtable                 1.8-2     2016-02-05 CRAN (R 3.5.0)
##  XVector                0.20.0    2018-05-01 Bioconductor
##  yaml                   2.1.19    2018-05-01 CRAN (R 3.5.0)
##  zlibbioc               1.26.0    2018-05-01 Bioconductor

## Software versions

The statistical methods were evaluated using the following software versions: *DRIMSeq* - 1.8.0, *DEXSeq* - 1.26.0, *stageR* - 1.2.21, *tximport* - 1.8.0, *DESeq2* - 1.20.0, *EBSeq* - 1.20.0, *edgeR* - 3.22.2, *limma* - 3.36.1, *RATs* - 0.6.4, *samr* - 2.0, *sleuth* - 0.29.0, *SUPPA2* - 2.3. The samples were quantified with *Salmon* version 0.10.0 and *kallisto* version 0.44.0. *polyester* version 1.16.0 and *alpine* version 1.6.0 were used in generating the simulated dataset.

## Data availability

The simulated paired-end read FASTQ files have been uploaded in three batches of eight samples each to Zenodo-

https://doi.org/10.5281/zenodo.1291375 ^26^

https://doi.org/10.5281/zenodo.1291404 ^27^

https://doi.org/10.5281/zenodo.1291443 ^28^

The quantification files are also available as a separate Zenodo dataset: 10.5281/zenodo.1291522 ^29^.

The scripts used to generate the simulated dataset are available at the simulation GitHub repository ( https://github.com/mikelove/swimdown) and archived here- https://doi.org/10.5281/zenodo.1410443 ^25^.

The counts associated with Soneson *et al*. ^33^ have been deposited to Zenodo ( 10.5281/zenodo.1409201 ^47^).

All data is available under a CC BY 4.0 license.

## Software availability

1. All software used in1 this workflow is available as part of Bioconductor version 3.7.
2. Source code for the workflow: https://github.com/mikelove/rnaseqDTU
3. Link to archived source code as at time of publication: https://doi.org/10.5281/zenodo.1410442 ^48^
4. License: Artistic-2.0

## Supplementary material

Supplementary File 1 - PDF file containing Supplementary Figure 1–Supplementary Figure 11
