# Supplementary material for: Swimming downstream: statistical analysis of differential transcript usage following Salmon quantification
Source: F1000Res. 2018 Oct 1;7:952. Originally published 2018 Jun 27. [Version 3] doi: 10.12688/f1000research.15398.3 (PMC6178912; doi:10.12688/f1000research.15398.3)
Supplement: Supplementary file 1 [file f1000research-7-17982-s0000.tgz › b2116dd5-e022-46f9-89a5-abc1504d20c5_supp_file_1_new.pdf]

Supplementary Figures

Michael I. Love, Charlotte Soneson, Rob Patro

September 7, 2018

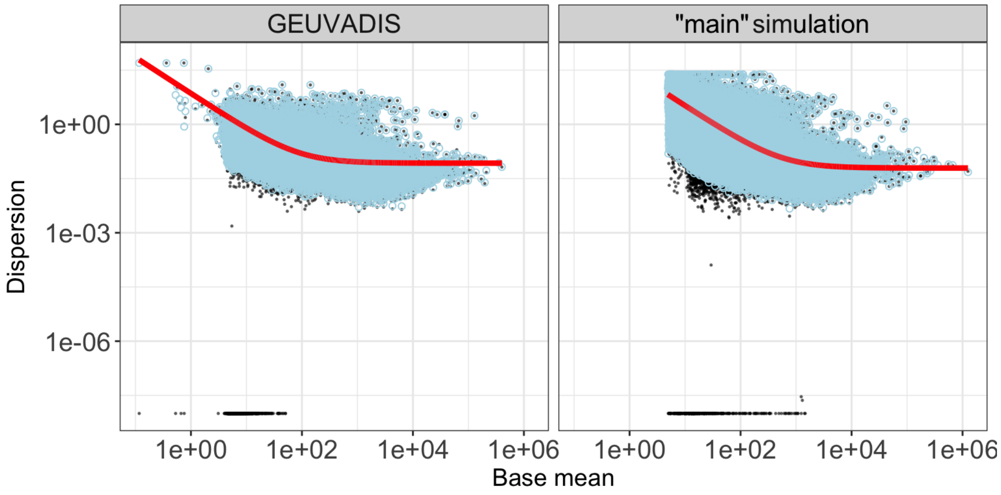

Supplementary Figure 1: Dispersion-over-mean comparison plot produced by *countsimQC*. The left panel shows *DESeq2* estimates of dispersion per gene over the mean of normalized counts from the GEUVADIS project, provided by the Recount2 project ($n = 458$ non-duplicated samples). All of these 458 samples were used to estimate mean and dispersion values for a Negative Binomial distribution, accounting for variation due to sequencing center and human population. The "main" simulation dataset was then constructed by drawing mean and dispersions parameters from this joint distribution of the estimates from 458 samples of the GEUVADIS project. The right panel shows estimates of dispersion per transcript over the mean of normalized counts for *Salmon* estimated transcript counts for the "main" simulated dataset (the 12 vs 12 comparison), showing only the transcripts where the mean of counts over samples was greater than 5. Black points indicate maximum likelihood estimates (Cox-Reid adjusted), blue points indicate posterior estimates, and the red line indicates the parametric trend line. Points at the bottom of the plot indicate maximum likelihood estimates of 10^{-8}. The design formula provided to *countsimQC* included sequencing center and population for GEUVADIS, and the condition variable for the simulated dataset. The full *countsimQC* report can be found at `https://github.com/mikelove/swimdown/tree/master/countsimqc`.

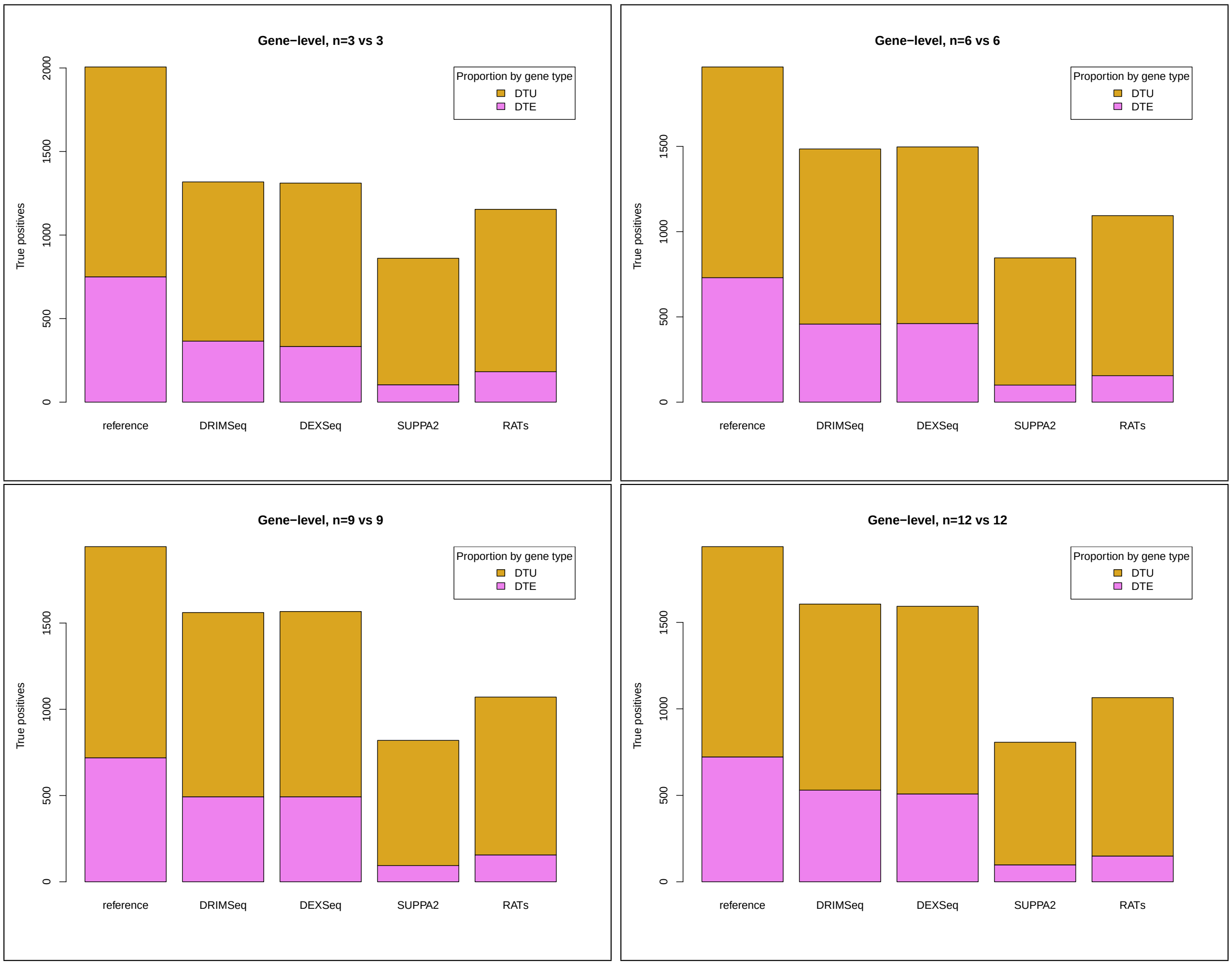

Supplementary Figure 2: Breakdown plots of true positives for differential transcript usage analysis at the gene level for the “main” simulation. From the top left, the panel of plots indicates a per-group sample size of 3, 6, 9, and 12, as indicated in the plot title. The far left bar in each panel, the “reference”, indicates the genes with differential transcript usage and how they are divided between the simulation gene type, whether “DTU” (two transcripts with swapped expression), or “DTE” (one transcript with differential expression, other transcripts with constant expression). Because some fraction of the simulated “DTE” genes had only a single expressed transcript (and so proportions did not change), there were, in the end, more “DTU” genes than “DTE” genes contributing to the total set of genes with differential transcript usage.

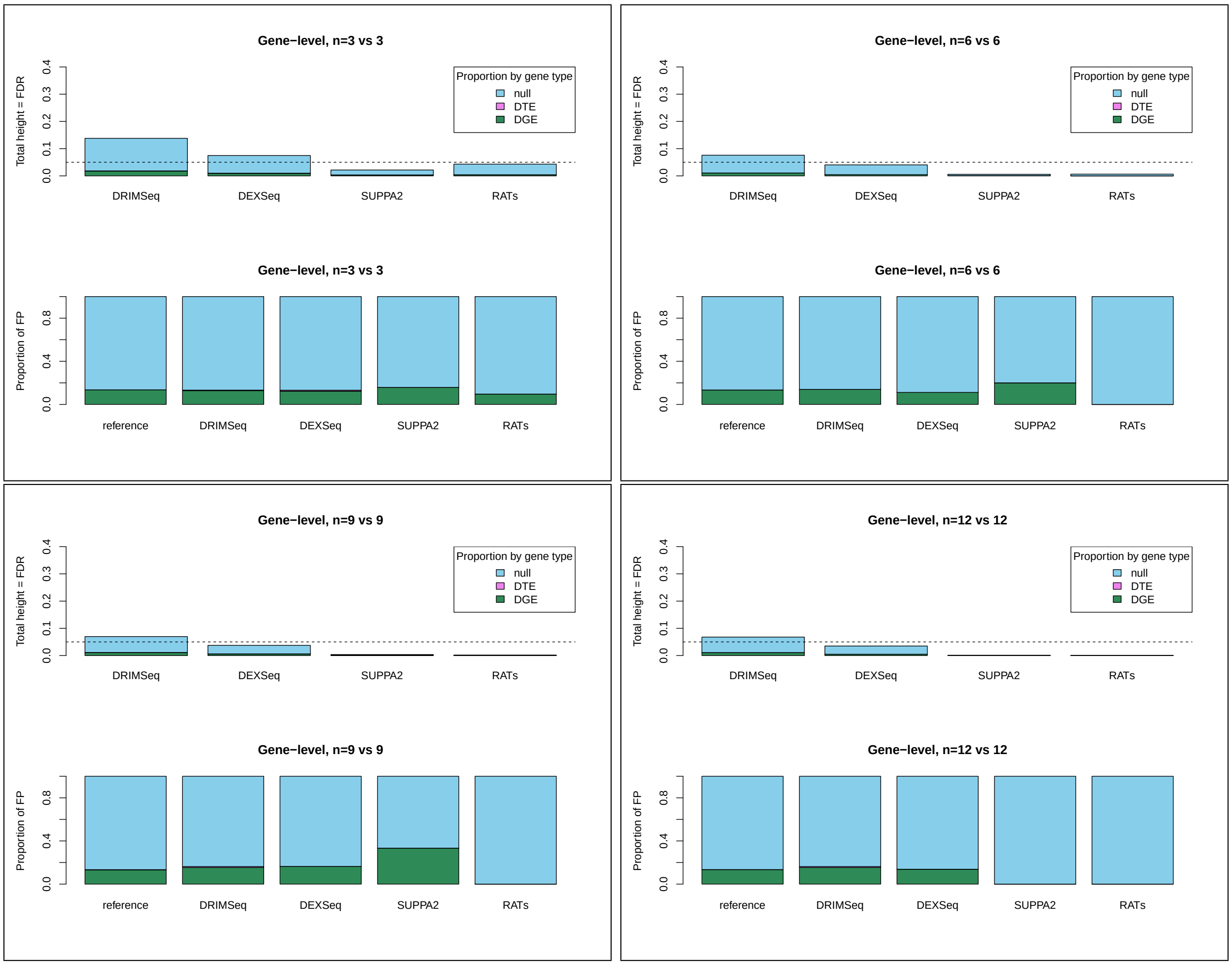

Supplementary Figure 3: Breakdown plots of false positives for differential transcript usage analysis at the gene level for the "main" simulation. From the top left, the panel of plots indicates a per-group sample size of 3, 6, 9, and 12, as indicated in the plot title. Within each panel, the bars in the top bar plot have a total height indicating the observed gene-level FDR for the method, and the bars are divided into the contributions to the false positive count from different simulated gene types: either "null" genes, "DTE" genes in which there was only one transcript expressed, or "DGE" genes. Within each panel, the bottom bar plot gives the standardized proportion per gene type, with a "reference" bar on the far left indicating the proportions of simulated gene type for all of the genes without differential transcript usage.

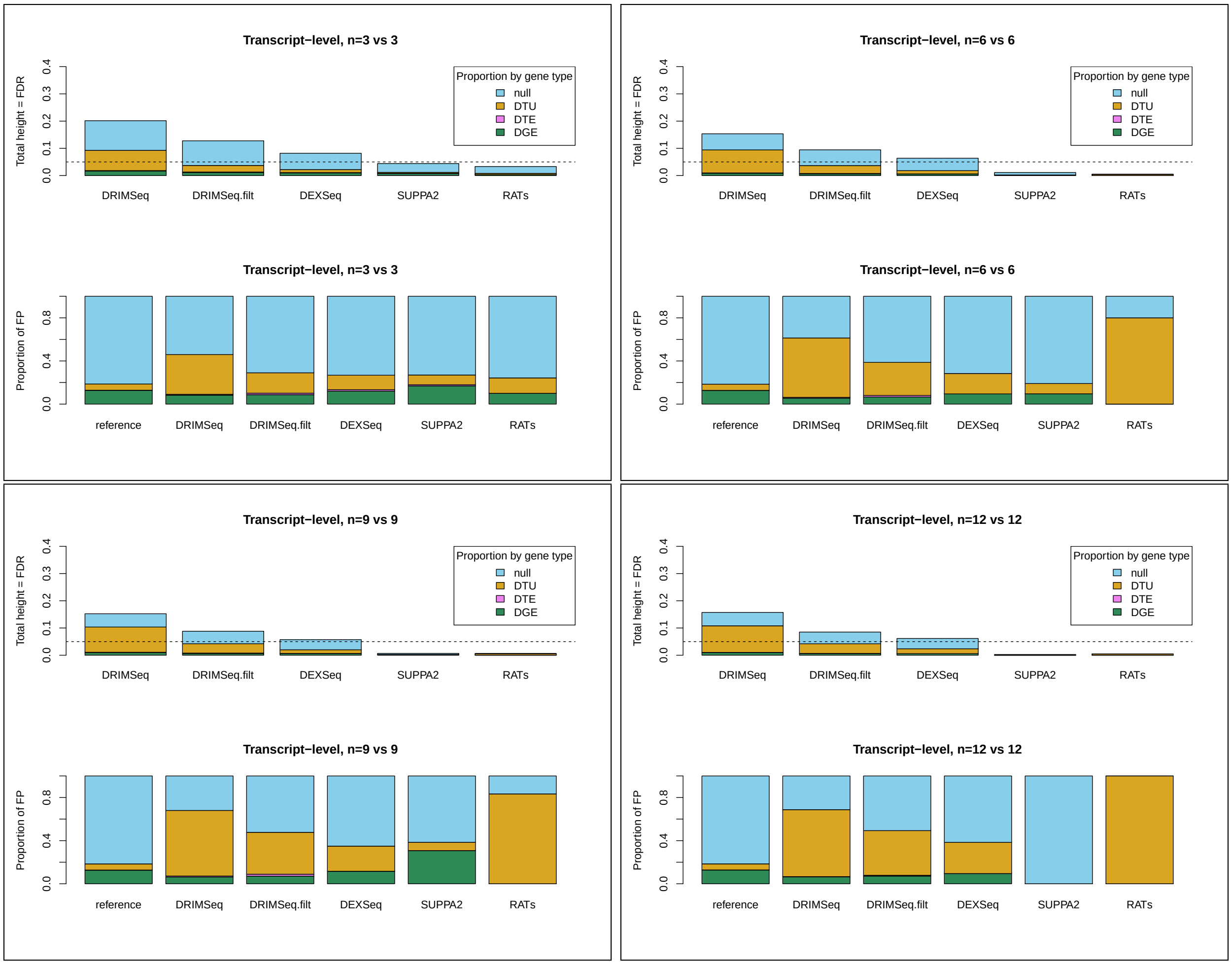

Supplementary Figure 4: Breakdown plots of false positives for differential transcript usage analysis at the transcript level for the "main" simulation. From the top left, the panel of plots indicates a per-group sample size of 3, 6, 9, and 12, as indicated in the plot title. Within each panel, the bars in the top bar plot have a total height indicating the observed transcript-level FDR for the method, and the bars are divided into the contributions to the false positive count from different simulated gene types: either transcripts from "null", "DTU", "DTE" or "DGE" genes. Note that all of the simulated gene types can produce false positive *transcript* calls for differential transcript usage, as the "DTU" genes can have wrongly identified transcripts: transcripts other than the two which had expression values swapped in the simulation. Within each panel, the bottom bar plot gives the standardized proportion per gene type, with a "reference" bar on the far left indicating the proportions of simulated gene type for all of the transcripts that did not participate in differential transcript usage.

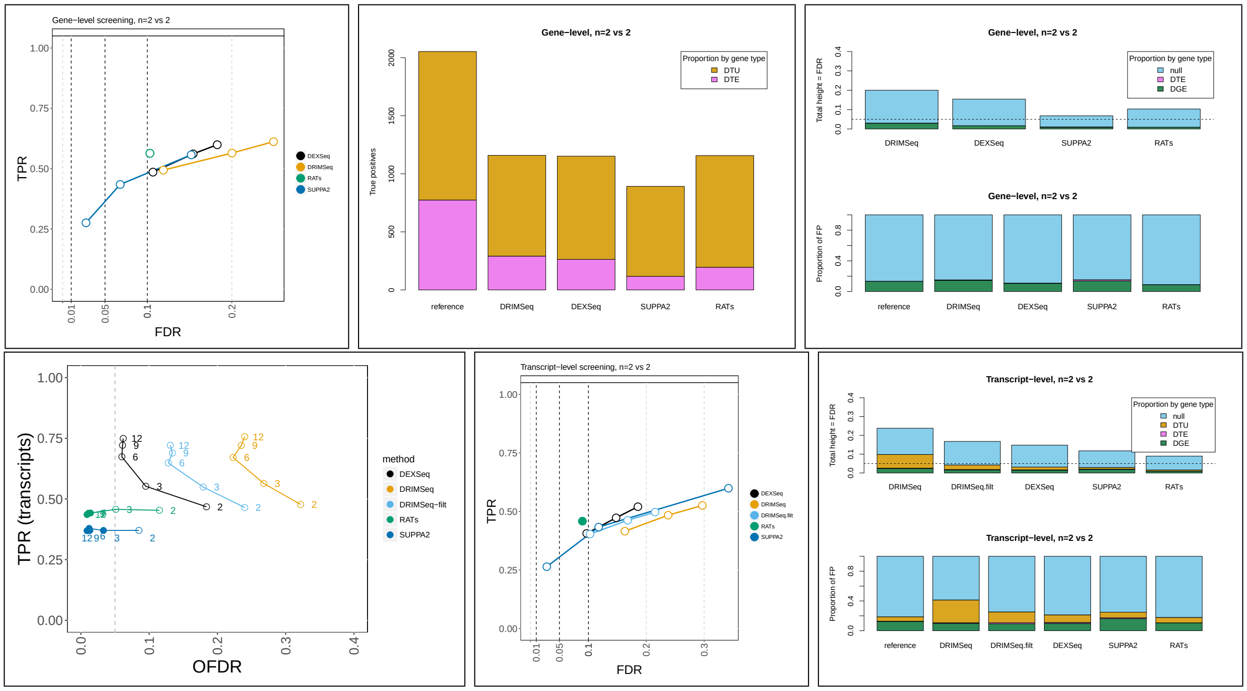

Supplementary Figure 5: Evaluation plots for differential transcript usage for a sample size of 2 vs 2. On the top row: gene-level sensitivity over false discovery rate, gene-level breakdown of true postives, gene-level, breakdown of false postives. On the bottom row: transcript-level sensitivity over OFDR including the 2 vs 2 case, transcript-level sensitivity over FDR, and transcript-level breakdown of false positives. The 2 vs 2 case had much higher observed FDR and lower sensitivity for all methods.

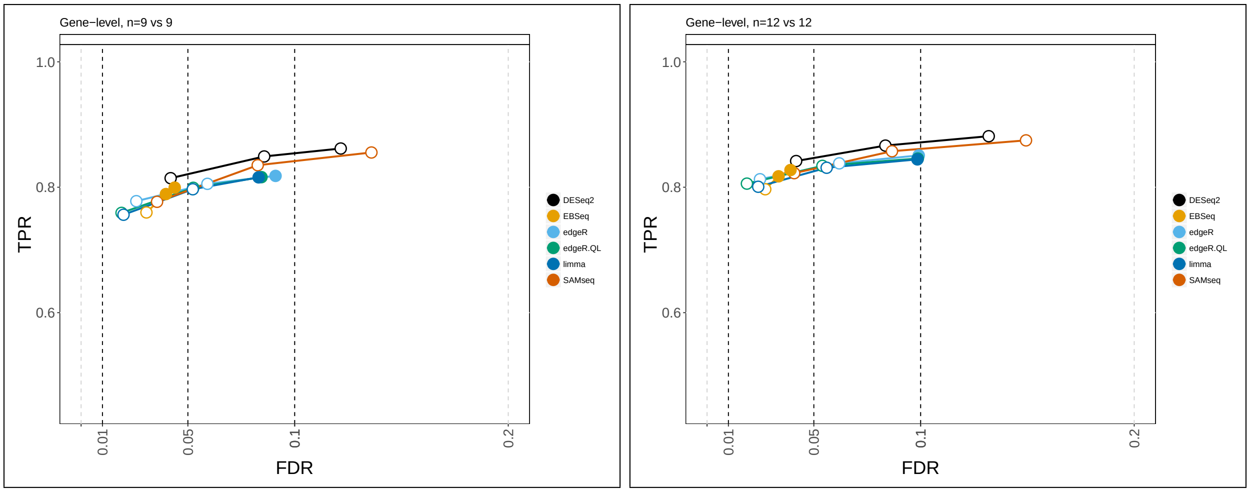

Supplementary Figure 6: Gene-level sensitivity over false discovery rate for the DGE evaluation of the "main" simulation, for sample sizes 9 vs 9 (left) and 12 vs 12 (right). These plots differs from the two in the text in that they fix the x-axis to the range $[0, 0.2]$.

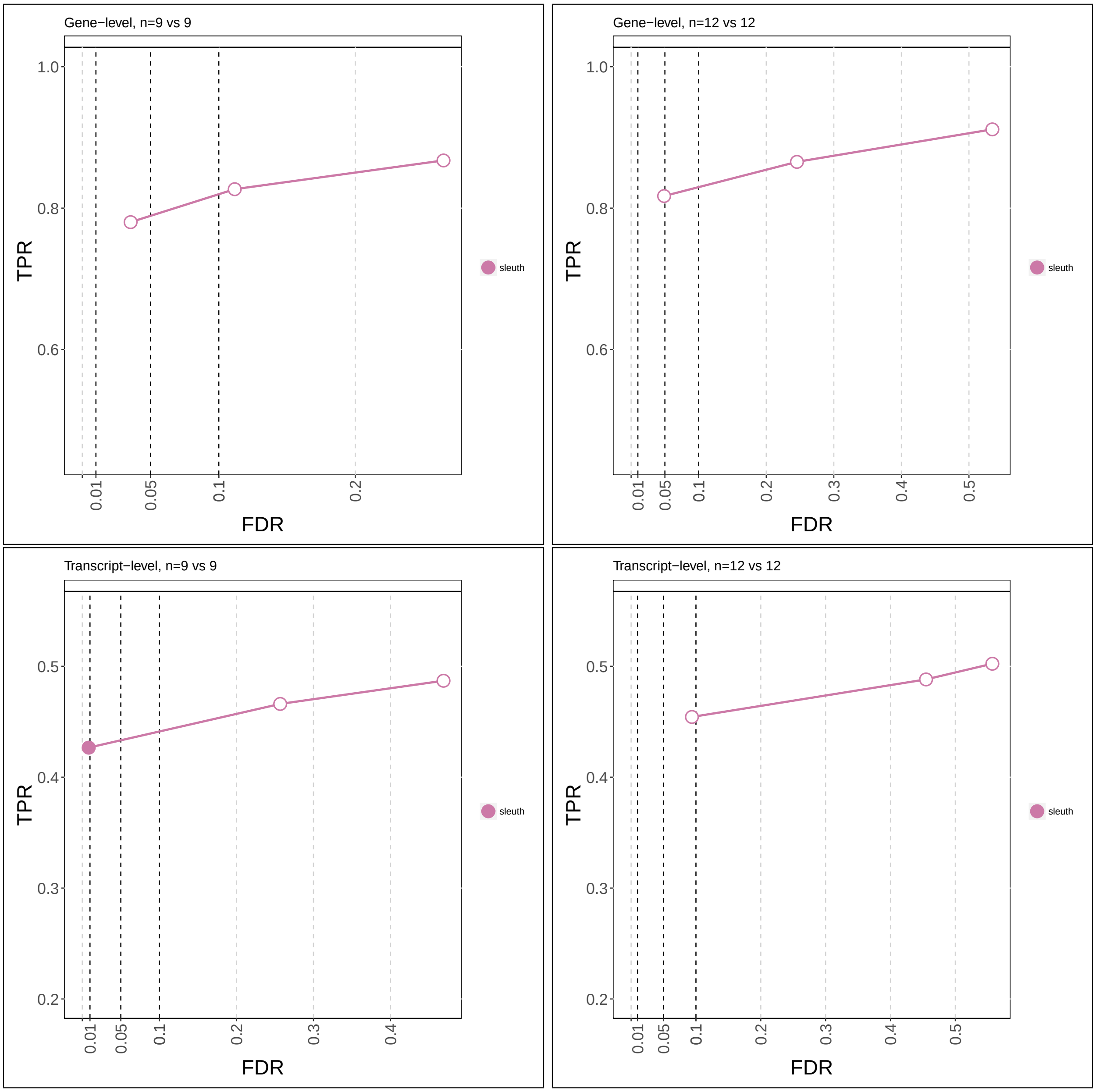

Supplementary Figure 7: An additional "uniform coverage" simulation to assess the false discovery rate control for *sleuth* at per-group sample size of 9 (left column) and 12 (right column), at the gene level (top row) and the transcript level (bottom row). To determine whether the excess observed FDR was due to the inclusion of realistic fragment GC coverage in the "main" simulation, for this "uniform coverage" simulation, fragments were instead drawn uniformly from positions on the transcripts. The dispersion-mean relationship was kept the same, drawing from the joint distribution of estimates on the GEUVADIS dataset ($n = 458$).

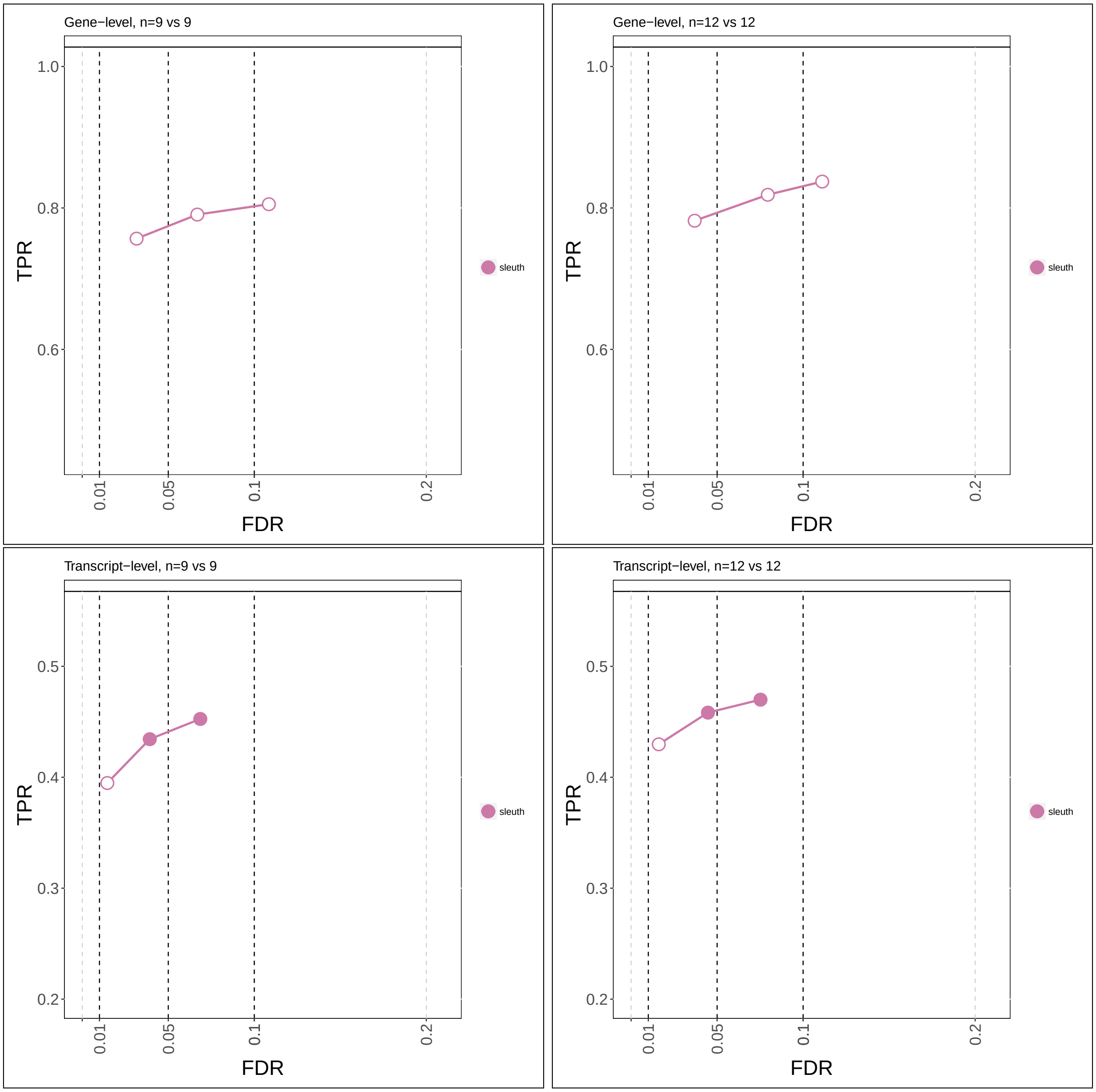

Supplementary Figure 8: An additional "low DE" simulation to assess the false discovery rate control for *sleuth*, at per-group sample size of 9 (left column) and 12 (right column), at the gene level (top row) and transcript level (bottom row). For this "low DE" simulation, realistic fragment GC bias was used as in the "main" simulation, but the percent of genes with DGE, DTE and DTU was lowered from 10% to 5% each. This modification of the simulation by reducing the proportion of transcripts with differential expression helped to regain control of FDR for *sleuth*.

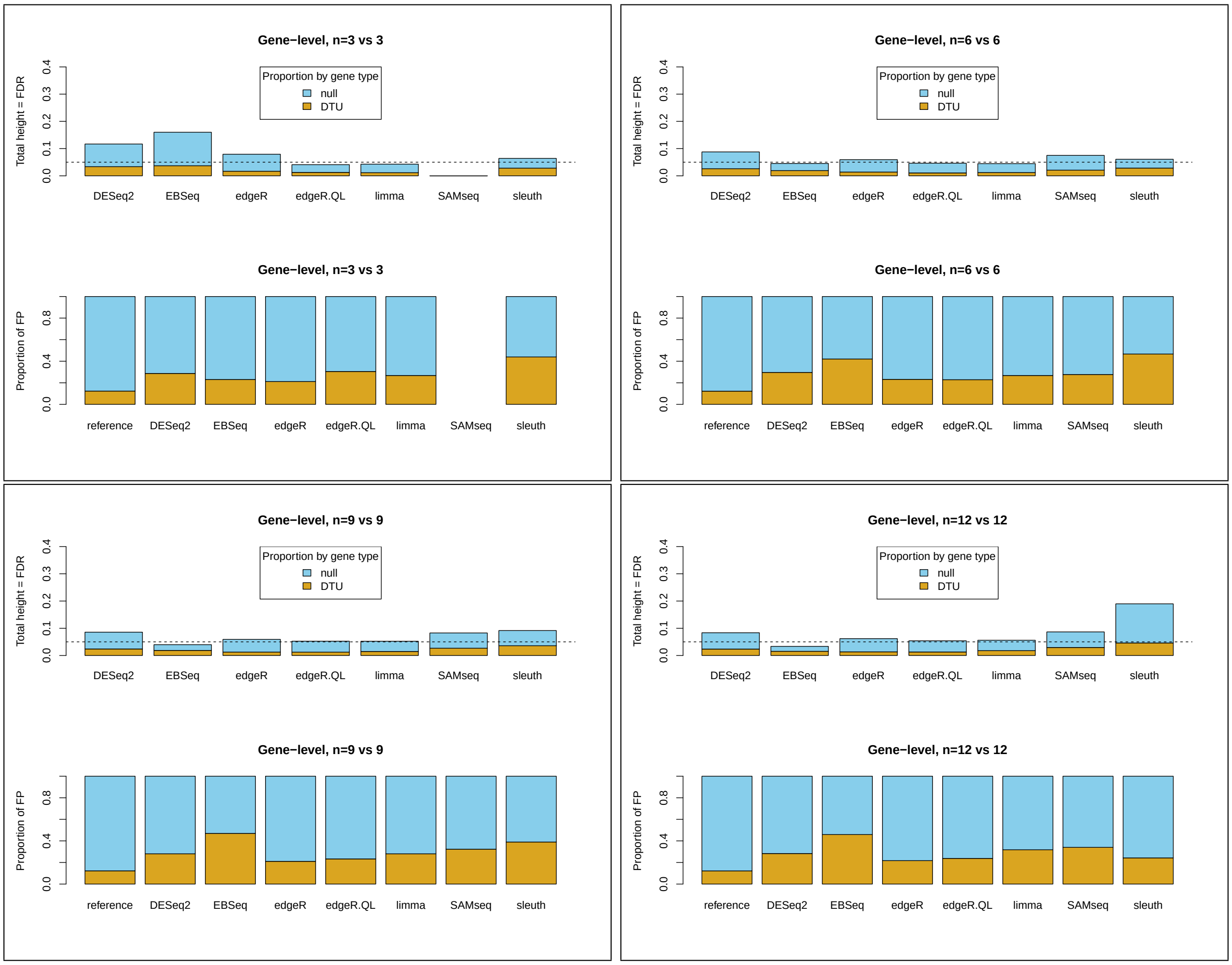

Supplementary Figure 9: Breakdown plots of false positives for differential gene expression analysis for the "main" simulation. From the top left, the panel of plots indicates a per-group sample size of 3, 6, 9, and 12, as indicated in the plot title. Within each panel, the bars in the top bar plot have a total height indicating the observed gene-level FDR for the method, and the bars are divided into the contributions to the false positive count from different simulated gene types: either "null" or "DTU" genes. Within each panel, the bottom bar plot gives the standardized proportion per gene type, with a "reference" bar on the far left indicating the proportions of simulated gene type for all of the genes without changes in total gene expression.

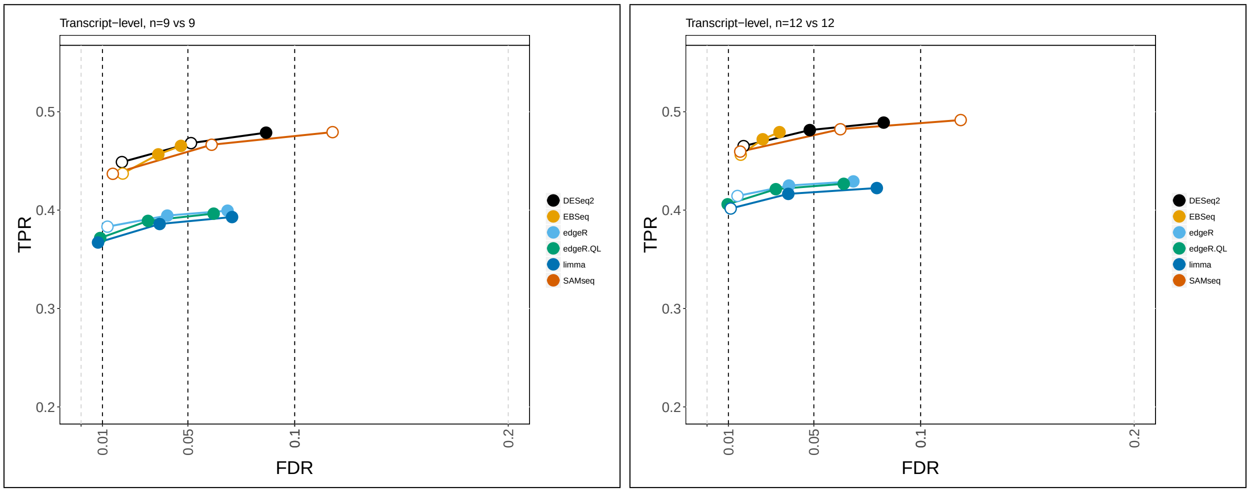

Supplementary Figure 10: Transcript-level sensitivity over false discovery rate for the DTE evaluation of the "main" simulation, for sample sizes 9 vs 9 (left) and 12 vs 12 (right). These plots differs from the two in the text in that they fix the x-axis to the range $[0, 0.2]$.

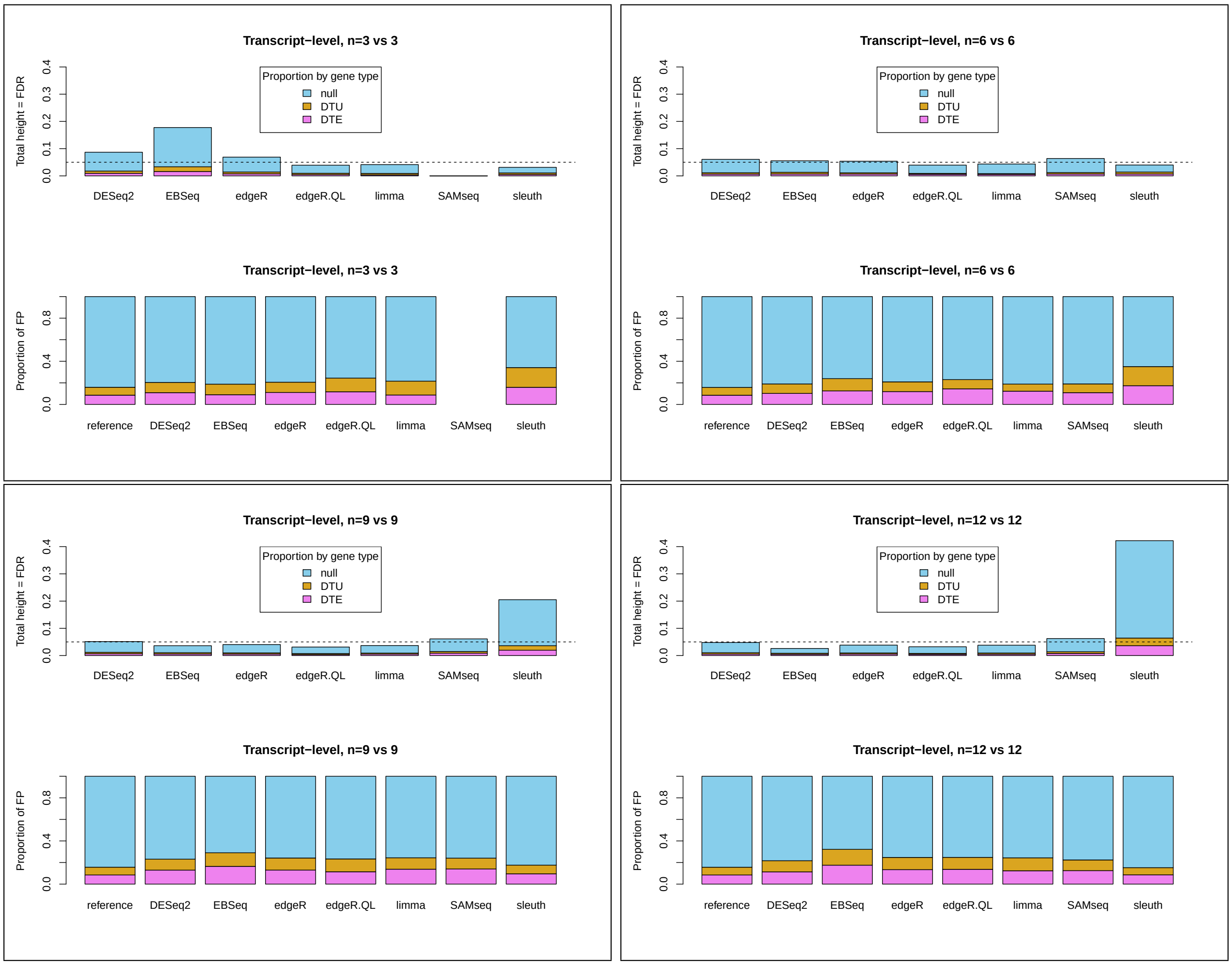

Supplementary Figure 11: Breakdown plots of false positives for differential transcript expression analysis for the "main" simulation. From the top left, the panel of plots indicates a per-group sample size of 3, 6, 9, and 12, as indicated in the plot title. Within each panel, the bars in the top bar plot have a total height indicating the observed transcript-level FDR for the method, and the bars are divided into the contributions to the false positive count from different simulated gene types: either "null", "DTU" or "DTE" genes. Within each panel, the bottom bar plot gives the standardized proportion per gene type, with a "reference" bar on the far left indicating the proportions of simulated gene type for all of the transcripts without differential expression.
