# Supplementary material for: Swimming downstream: statistical analysis of differential transcript usage following Salmon quantification
Source: F1000Res. 2018 Oct 1;7:952. Originally published 2018 Jun 27. [Version 3] doi: 10.12688/f1000research.15398.3 (PMC6178912; doi:10.12688/f1000research.15398.3)
Supplement: Peer review history

# Peer review: Referee response for version 3

**DOI:** 10.5256/f1000research.17982.r38961

**Published:** 2018-11-12

**Authors:** Kristoffer Vitting-Seerup, Malte Thodberg

**Affiliations:** 1 Department of Biology, Biotech Research and Innovation Centre, University of Copenhagen, Copenhagen, Denmark

Although we still believe some of the suggestions from our last report would make the article appeal to a larger audience we agree with the authors that they are not necessary as the changes in the last update does take care of our major concern.

We have read this submission. We believe that we have an appropriate level of expertise to confirm that it is of an acceptable scientific standard.

# Peer review: Referee response for version 3

**DOI:** 10.5256/f1000research.17982.r38962

**Published:** 2018-10-08

**Authors:** Nick Schurch

**Affiliations:** 1 Division of Computational Biology, School of Life Sciences, University of Dundee, Dundee, UK

The authors have made a great improvements to the article along the lines suggested by the referees. 

 I still think there is a considerable amount of work that could be added to expand the study, as I suggested in my original report. I understand, however, that the authors feel that a more expansive exploration of the tool parameter space and the addition of  assessment on real data is beyond the scope of the article. I am of the opinion that, with these suggestions in hand, they are best positioned to define the scope of their own work. In my opinion the improvements they have made round out the article nicely and certainly make it acceptable for publication and indexing.

 Minor comment:

 1) Introduction, line 1: This sentence is awkward. I think total gene expression is self explanatory, but if the authors feel it requires more explanation perhaps they could make the sentense clearer - something like:

 "RNA-seq experiments across groups of samples can be analyzed to detect differences in the total expression of all isoforms of a gene and, additionally, differences in transcript isoform usage within a gene."

 P.S. I particularly like the use of the hex 'stickers' for the tools in the workflow diagram - nice.

I have read this submission. I believe that I have an appropriate level of expertise to confirm that it is of an acceptable scientific standard.

# Peer review: Referee response for version 2

**DOI:** 10.5256/f1000research.17766.r38328

**Published:** 2018-10-01

**Authors:** Alicia Oshlack, Marek Cmero

**Affiliations:** 1 Murdoch Children's Research Institute, Royal Children's Hospital, Parkville, Vic, Australia

We feel the authors have done an excellent job in improving the structure and clarity of the paper. We believe the improved introduction and workflow diagram will be particularly helpful to readers and we accept the paper for indexing.

 A few very minor points remain:

  

- p18, could the authors please rewrite this sentence for clarity: "To repeat, the set of genes passing screening should not have more than 5% of either genes which have in fact no DTU or genes which contain a transcript with an adjusted p-value less than 5% which do not participate in DTU."

- p32, second paragraph: could the authors hypothesise why there was a slight increase of false positives for DTU genes in the DGE analysis?

- p34, fourth paragraph: mentions of SUPPA2, DEXseq and leafcutter are repeated across two sentences in the same context.

We have read this submission. We believe that we have an appropriate level of expertise to confirm that it is of an acceptable scientific standard.

# Peer review: Referee response for version 2

**DOI:** 10.5256/f1000research.17766.r38330

**Published:** 2018-09-28

**Authors:** Kristoffer Vitting-Seerup, Malte Thodberg

**Affiliations:** 1 Department of Biology, Biotech Research and Innovation Centre, University of Copenhagen, Copenhagen, Denmark

**Summary**

 In “Swimming downstream: statistical analysis of differential transcript usage following Salmon quantification” Love *et al* presents a combined workflow and benchmark for differential transcript usage. This is a vital paper as there is no consensus on which differential transcript usage tools works better (here addressed by the benchmark part) and very few people are aware of the feasibility of analysis of differential transcript usage – something the workflow can hopefully help with. Of special note is the extent to which open source have been embraced by Love *et al* – an approach that is commendable (and worthy repeating).

 In the revised version (version 2) the workflow and benchmark have been separated and the workflow has been simplified which, together with the overview figure and the general improvements makes the article much more readable. With regards to analysis especially the benchmark session have been extended. In summary, the revised manuscript version address the majority of our concerns but we still believe the introduction and benchmark could be improved, as detailed below.

**Comments**

- The authors have updated the benchmark to use testForDEU instead of nbinomLRT for the DEXSeq analysis (verified at https://github.com/mikelove/rnaseqDTU) but seem to accidentally have forgotten to correct this in the workflow part of the article (page 17)
- The introduction lacks:
  - A section describing why differential transcript usage are of interest in the first place.
  - A layman introduction to the terms DTU, DTE and DGE. Here it should also be highlighted that DTU can be analyzed both at transcript and gene level (since it is confusing for people not in the field). A more detailed introduction here would also make the concepts behind StageR clearer to readers not familiar with the subject.
- The workflow/benchmark still contains large chunks of text that belongs in the introduction/methods. Specifically we are thinking of:
  - The scaling section (page 9)
    - It could be even further highlighted that the difference between “scaledTPM” and the other scaling methods is whether the issue with transcript lengths are normalized away. This could also be highlighted by making a figure like Fig1 in Trapnell *et al* which instead illustrates the DTU problem.
    - We further recommend that the documentation for tximport with regards to scaling is updated with the descriptions made here.
  - The section regarding the theory behind DRIMSeq (bottom of page 12) (could be with the rest of the theory regarding DRIMSeq (page 7)
  - The section regarding StageR (page 14)
  - The article would benefit from a one or two sentence layman introduction to all the tools (for people not in the field).
- The DTU benchmark should also include an analysis on unmodified simulated data to test how many false positives are found if there truly are no DTU (which might be the case for some datasets). We suggest comparing samples internally in either set 1 or set 2.
- To reflect a very common use case scenario the DGE / DTE benchmark should also be performed with 2 replicates.
- All iCOBRA plots would benefit from zooming in on the y-axis to the min and max of any tool across all samples (currently much of the y-axis range is never used – which just squish all samples together). This is especially problematic for the DGE/DTE benchmark.
- The section on page 30 describing the results in figure 13 is very hard to understand.
- Given the success of repurposing DEXSeq to DTU, the good performance of limma/edgeR for DTE/DGE and the recent incorporation of RATS, the current benchmark could also test a repurposing of limma’s (and edgeR’s) differential exon usage test (diffsplice/diffSpliceDGE). This is optional – but it would be a huge step forward for testing differential isoform usage as it would bring a lot of clarity to the field.

We have read this submission. We believe that we have an appropriate level of expertise to confirm that it is of an acceptable scientific standard, however we have significant reservations, as outlined above.

# Response

**Published:** 2018-09-28

**Authors:** Michael Love

**Affiliations:** University of North Carolina at Chapel Hill, USA

Thank you for reviewing our revised version.

 Yes, unfortunately, while we updated the results in version 2 to use testForDEU and the code in the Bioconductor git repository, we neglected to update the displayed code chunk with nbinomLRT to testForDEU(). A version 3 is already in production which will show the correct code.

 Regarding the placement of the text in various sections, an F1000Research workflow article does not have exactly the same structure as a typical research article, and we feel our current structure is appropriate and fits with the pattern of other Bioconductor workflow article published in F1000Research. In particular, we feel it is appropriate to introduce concepts as they arise in the natural flow of the workflow, rather than all in the Introduction or in the terse Methods section.

 In version 2, we feel we have included sufficient background and citations to research and review articles about the importance of detecting DTU in the Introduction (see the first paragraph of the Introduction).

 For full details on the theory of DRIMSeq, we have instead recommended that readers go to the primary source.

 We will not be adding additional simulations or evaluations to the workflow in the near future, as it already contains much more evaluations and simulation than the comparable workflows published in the Bioconductor channel of F1000Research.

# Peer review: Referee response for version 1

**DOI:** 10.5256/f1000research.16780.r35682

**Published:** 2018-08-13

**Authors:** Nick Schurch

**Affiliations:** 1 Division of Computational Biology, School of Life Sciences, University of Dundee, Dundee, UK

In 'Swimming downstream: statistical analysis of differential transcript usage following Salmon quantification' Love, Sonesson & Patro present both 1) a workflow for identifying the signatures of differential transcript usage between RNA-seq samples in two conditions, based on a suite of tools, and 2) a benchmarking analysis of the performance of these tools based on simulated data. The aims of this work are laudable and I have no doubt it will be a valuable addition to the literature, the resulting paper suffers from several flaws and needs considerable additional work, in my opinion.

**Major comments:**

 1) The intermingling of the benchmarking and workflow sections of this manuscript make the text confused and difficult to read. I'd suggest that the authors either restructure the manuscript beginning with the workflow section and then following with the benchmarking section, or split the work in to two and concentrate separately on the two areas.

 2) This work is listed as a Method article. I am not convinced that an example of stringing existing tools together fits the description required for this section (that is: "Method Articles describe a new experimental, observational, or computational method, test or procedure (basic or clinical research)."). The benchmarking part of the work is better suited to a Research Article, whilst the workflow part is more like a computational protocol and might be better suited for publication as a Study Protocol.

 3) Quantifying transcript expression from RNA-seq data is challenging but has become common-place and relatively straight-forward thanks to the development of high-performance tools such Salmon and Kallisto. These tools typically provide a transcripts-per-million estimation of a transcripts expression. With these quantifications in place the inevitable, and even more challenging, next step is to identify those transcripts where their expression is changing between samples. To date there has not been a clear data-driven exploration of the underlying statistical properties of TPM quantifications (or estimated transcript counts from TPMs) as a function of biological and technical replication - instead, much as was the case for differential gene expression from RNA-seq data until relatively recently - the tools for identifying DTE are built on the strong assumption of a distribution for the quantifications and, typically, assume a negative binomial distribution. Although this looks to be a good assumption in the case of gene expression, it is far from clear to me that the assumption of a negative binomial distribution for the distribution of a transcripts TPM or estimated counts across biological replicates is a good assumption for TPMs or estimated counts from TPMs, particularly given that - in the context of biological DTU - the expression of a transcript can be strongly correlated with the other child transcripts of the gene. The fixed per-gene dispersion section seems like the beginnings of an exploration in this area but this assumption too is without any justification. Perhaps the authors could use some highly replicated data from a complex eukaryote to actually measure these distributions and give clarity on whether these assumptions are valid? Or, failing that, explore the impact of different potential distributions of the tool performance?

 4) The entire discussion section of the benchmarking results is essentially missing and the current discussion section of more like a brief conclusion. Points that I would like to see the authors discuss in detail include:

- The low overall TPRs exhibited by all the tools; 25-80% for DTU, 50-80% for DGE & only 20-50% for DTE. What this means for these field and how might these be improved?
- The TPR/FPR performance of the tools not only as function of the sample size, but also as a function of the annotation used in the original transcript quantitations, as a function of the effect-size threshold used and as a function of the low-count-rate filtering used for each tool. These are all critical parameters in the tools performance.
- An expanded discussion of the extremely poor FPR performance of DRIMseq, that is largely glossed-over in the current text. Why is DRIM-seq performing so poorly? It is more or less dependant on the specific parameters used, or the details of the simulated data, than the other tools - or is it just generically over-sensitive across all the parameter space.
- The overlap between the sets of DTU, DGE & DTE identified by each tool, instead the authors just give us some numbers and the TPR/FPR performance metrics. Are these tools reliably identifying the same thing or are they finding wildly different sets of results? (but please, no Venn diagrams! I can respectfully recommend upsetR for this kind of plot).
- The use of p-values, adjusted or not, as a threshold for subsetting these results for scientific relevance - particularly given Blume et. al 2018 ^1^.
- Some discussion of why the authors limit themselves to discussing DRIMseq, DEXSeq and SUPPA2 despite listing five additional alternative methods in the introduction. Alternatively, the authors could include these tools in their benchmarking, particularly if they decided to split the work into two papers with one of these focussing on the benchmarking.
- Some discussion of the impact that the development of long-read sequencing of native RNAs will have on this field, these tools, and their results in the next few years - perhaps the authors could even use some of the publically available data from the Oxford Nanopore RNA consortium (https://github.com/nanopore-wgs-consortium/NA12878/blob/master/RNA.md) to contrast the performance of this new technology with the tools they examine here for detecting DTE and DTU.
- How do these tools cope with RNA-seq experiments with more complex designs? For example, what about if there are 7 conditions, or a time-series (see for example Calixto et. al., 2018 ^2^? What approaches would the authors then recommend?

5) No effort has been made to test these workflows with real data with validated instances of DTU. These exist in the published literature. For a workflow description this is fine, but for the benchmarking aspect of the work I would like to see the authors use this pipeline in anger, with real data, and see what the results are and how they match up with the validated results.

 6) The introduction does not motivate the importance of identifying DTU in biology. I'd like to see the introduction present the biological relevance of DTU, the relative sparsity of existing validated DTU instances, and the scope DTU has for being an explored layer of regulation for basic biological processes.

 7) The only conclusion from the paper seems to be that the authors recommend the use of stageR - based largely on the fact that its two-stage model matches what the authors think a typical analysis workflow is. This conclusion may be sound advice but a) this paper does not present any compelling *evidence* that this is a typical workflow, and b) stageR is not really what this paper is about" Indeed, here stageR is used as a framework to assist with assessing the performance of the other tools. I'd like to see the authors instead draw some clear conclusions about which tools are the best to use for identifying DTU.

**Minor Comments:**

 1) The workflow section really needs some workflow diagrams to highlight the chain for each tool and where they are similar and different.

 2) The plots in the paper are not as high quality as I'd expect:

  - Figures need to be higher resolution (this may be the journals fault, not the authors)

  - Figures 3,5,6,8,12 & 13 are multi-panel figures with the same axes on each figure. They would benefit from being plotted with shared axes allowing the performance between different samples sizes to be more clearly visible to the reader.

  - Figures 9-11: perhaps consider using a multi-panel 2d histogram to show the density profiles for each group, or at least using a better point symbol.

I have read this submission. I believe that I have an appropriate level of expertise to confirm that it is of an acceptable scientific standard, however I have significant reservations, as outlined above.

## References

[1] BlumeJDD'Agostino McGowanLDupontWDGreevyRA: Second-generation p-values: Improved rigor, reproducibility, & transparency in statistical analyses. *PLoS One*.2018;13(3) : 10.1371/journal.pone.0188299 e0188299 10.1371/journal.pone.0188299 29565985PMC5863943

[2] CalixtoCPGGuoWJamesABTzioutziouNAEntizneJCPanterPEKnightHNimmoHGZhangRBrownJWS: Rapid and Dynamic Alternative Splicing Impacts the Arabidopsis Cold Response Transcriptome. *Plant Cell*.2018;30(7) : 10.1105/tpc.18.00177 1424-1444 10.1105/tpc.18.00177 29764987PMC6096597

# Response

**Published:** 2018-09-11

**Authors:** Michael Love

**Affiliations:** University of North Carolina at Chapel Hill, USA

We thank all reviewers for their insightful comments and suggestions that we feel have greatly improved the readability and usefulness of the workflow. We summarize the main changes and then address reviewer-specific comments point-by-point:

- We have addressed all minor text or grammatical suggestions by the reviewers.
- We have re-organized the article into distinct and more separated Workflow and Evaluation sections, which was suggested by all reviewers. We begin the article with a clear outline, titled: "Structure of this article", which outlines the Workflow part and the Evaluation part. This outline has direct links to relevant sections and subsections which follow. We have also included an overview diagram of the methods and packages included in the Workflow section, and how they are interconnected.
- We have added to the Introduction more motivational text on why a DTU analysis is relevant for biology and biomedical research.
- We have added a large section describing the methods DEXSeq and DRIMSeq, before the Workflow section.
- We have expanded the original sections discussing counts-from-abundance and their use in the workflow, to make our use of the tximport method more clear.
- For the DEXSeq section, we have corrected an earlier incorrect use of nbinomLRT(), which is now replaced with the correct testForDEU(). The practical result is that DEXSeq performs somewhat less conservatively, but the original code was incorrect, and the fix is necessary. The incorrect use of nbinomLRT() in this context will now produce an error in future releases of Bioconductor, to avoid possible incorrect usage.
- We have added RATs to the DTU Evaluation.
- We now apply stageR to all DTU methods that are evaluated: DRIMSeq, DEXSeq, RATs, and SUPPA2. The RATs and SUPPA2 methods are described, but the code is not provided, as these packages are not part of the Workflow.
- We use consistent x-axes and y-axes whenever possible, and use PDF instead of JPG to reduce compression artifacts. When a consistent x-axis is not used in the main text, we include Supplementary Figures with the same plots with outlying methods dropped to keep the x-axis consistent.
- We use a palette in which colors are more discernable for color-blind readers
- In the Evaluation sections, we include additional plots which examine the simulated gene type source of false positives for the DTU, DGE, and DTE analyses.
- We added a new evaluation to examine performance differences between DRIMSeq and DEXSeq, using the identical simulated data that was used in Soneson et al (2016) and Nowicka and Robinson (2016).
- We have added a 2 vs 2 simulation for the DTU Evaluation.
- We added a brief overview description of all methods assessed in the DGE and DTE Evaluations.
- We have added more recommendations in the Discussion.

Reviewer-specific comments:

 1) We have followed the reviewer's suggestion, and have separated the Workflow and Evaluation sections, with an outline at the beginning clearly delineating the two sections, and an overview diagram.

 2) We originally submitted our Bioconductor workflow as a "Research" article, but the Editorial Office recommended to change the categorization to "Method", which is the categorization of many of the other Bioconductor workflows. Bioconductor workflows are not intended to introduce new computational methods or new software packages, but to demonstrate, with live code that resides in an Rmarkdown vignette within an R package structure, how to use a number of different existing Bioconductor packages to analyze a dataset. 

 We asked for comment from the Editorial Office on the recommended categorization of Bioconductor workflows under the F1000Research article types, and they provided us with the following statement:

 " *In general, Bioconductor workflows are classified as Method articles in F1000Research, since Research articles must present novel research findings, and Software Tool articles must present novel software tools. Since this article by Love et al neither presented novel research findings nor a new software tool, the F1000Research editorial office felt that classifying this article as a Method article was most appropriate. The majority of workflows submitted to the Bioconductor gateway will fall into this article type.*" -F1000Research Editorial Office

 3) We have followed the reviewer's suggestion and included, in addition to the fixed per-gene dispersion simulation, an additional simulation from Soneson et al. (2016), to assess differences between DRIMSeq and DEXSeq, the two methods that are the focus of the workflow. This simulation involved generation of Negative Binomial gene counts, and then the expression was distributed from genes to transcripts by per-sample draws from a Dirichlet distribution, with a minority of genes undergoing DTU across condition. Analysis of additional datasets, and a final determination of which type of data-generating process is closer to various real RNA-seq datasets, is beyond the scope of this workflow, but we feel that the existing simulations cover a range of possibilities and are useful to the readers of the workflow. We comment in a number of places on the limitations of the simulation, including in the overview:

 " *While the evaluations rely on simulated data, and are therefore relevant only to the extent that the simulation model and parameters reflect real data, we feel the evaluations are useful for a rough comparison of method performance, and for observing relative changes in performance for a given method as sample size increases.*"

 Also at the end of the DTU Evaluation:

 " *Again, a caveat of all of our comparative evaluations of DRIMSeq and DEXSeq is that we do not know whether various real RNA-seq experiments will more closely reflect heterogeneous dispersion or fixed dispersion within genes, or if the counts within gene are better modeled by distributing gene-level abundance to transcripts via a Dirichlet distribution as in Soneson et al (2016). However, we have examined simulations reflecting each of these cases, and confirmed that minimum count and minimum proportion filtering benefit both DRIMSeq and DEXSeq.*"

 4) We now include more discussion on the results of the evaluations in the Discussion, including a comment on statistical power. We include a breakdown of false positives by the simulated gene type. Further cross-section of all methods' performance by incomplete annotation, effect size filters, and various count or proportion filters is beyond the scope of the article. Complete analysis of overlap of calls across the various simulations and analyses is also beyond the scope of the article.

 We now explore DRIMSeq's performance in the "main" and "fixed per-gene dispersion" simulations, wherein we see that many of the excess false positives at the transcript-level arise from simulated DTU genes, so other transcripts not participating in DTU were being reported as significant. In the “main” simulation, where DRIMSeq has the most problem with FDR control, it only slightly exceeds a target 10% FDR at the gene level at per-group sample sizes 6 and higher. With proportion SD filtering, DRIMSeq at the transcript level also has small inflation of target 10% FDR for per-group sample sizes 6 and higher. 

 We now include RATs as an additional method evaluated on the "main" simulation for DTU analysis. RATs performs similar to SUPPA2, in that it nearly always controls the FDR, although in some cases, it displays higher gene-level sensitivity than SUPPA2. We do not intend the article to be a complete evaluation of all existing methods for DTU, but to compare the two Bioconductor methods that are the focus of the workflow with a few key DTU methods.

 Extended discussion of long-read sequencing is beyond the scope of the article, although we added the following comment to the workflow section on importing counts:

 " *If a different experiment is performed and a different quantification method used to produce counts per transcript which do not scale with transcript length, then the recommendation would be to use these counts per transcript directly. Examples of experiments producing counts per transcript that would potentially not scale with transcript length include counts of full-transcript-length or nearly-full-transcript-length reads, or counts of 3' tagged RNA-seq reads aggregated to transcript groups. In either case, the statistical methods for DTU could be provided directly with the transcript counts.*"

 A relevant quote from Nowicka and Robinson (2016) is:

 " *With emerging technologies that sequence longer DNA fragments (either truly or synthetically), we may see in the near future more direct counting of full-length transcripts, making transcript-level quantification more robust and accurate.*”

 In the "DTU testing" section, we now discuss how DEXSeq and DRIMSeq can be used to evaluate experiments with complex designs, with little limitation as long as the coefficients for each sample can be encoded as a design matrix multiplied by a vector of coefficients.

 5) Comprehensive evaluation of the methods on additional datasets is beyond the scope of the article.

 6) Following this and other reviewers' suggestion, we have now added motivation to the first part of the Introduction as to why DTU is relevant for biological or biomedical research.

 7) We have revised some of our description of the stageR framework to be more clear about why we recommend its use in a DTU workflow:

 " *It is likely that an investigator would want both a list of statistically significant genes and transcripts participating in DTU, and stageR provides error control on this pair of lists, assuming that the underlying tests are well calibrated.*"

 We also provide some more details in the Discussion regarding the various methods and their performance.

 Minor Comments:

 1) We have added an overview diagram as Figure 1.

 2) We have updated figures to be PDF instead of JPG, and made the axes more consistent when possible.

# Peer review: Referee response for version 1

**DOI:** 10.5256/f1000research.16780.r35546

**Published:** 2018-07-30

**Authors:** Alicia Oshlack, Marek Cmero

**Affiliations:** 1 Murdoch Children's Research Institute, Royal Children's Hospital, Parkville, Vic, Australia

A workflow to enable more people to perform differential transcript usage on their RNA-seq data set is a useful addition to the literature. Benchmarking methods and combinations of workflows are also an important part of the literature. In this manuscript, both things have been attempted, which unfortunately makes the manuscript a little blurred in its focus.

 We view a workflow as an instructional manuscript in which a step-by-step analysis can be reproduced with a new data set that a user wants to bring to the analysis. This is presented in the sections Quantification and data import and Statistical analysis of differential transcript usage and, in our view, should be the focus of the manuscript. These are complex analyses combining several packages with several alternative paths. It would really help the user if a flowchart for this analysis could be made that shows the common parts of the workflow (e.g. starting with a Salmon, importing into R), how the alternatives split and which packages are used for alternative parts of the workflow. For example, DRIMseq is an alternative to DEXseq, which can then be followed by stageR, and Suppa is a complete (parallel) workflow.

 The evaluation sections are somewhat useful and interesting in their own right, but rely on simulated data and are therefore not directly applicable to readers who are looking for workflows to guide them in their own data analysis. However, they do help users decide which workflows to choose in their own analysis.

 Overall we wonder if this manuscript could be two separate manuscripts: a workflow for DTU and an evaluation of methods based on simulated data? Another (preferable) alternative would be to only focus on DTU in the evaluation and keep the section Evaluation of methods for DTU as a guide to help the user to choose the workflow (with this clearly stated). We felt there were too many additional analysis introduced after this point which relied on more in-depth understanding of the DGE literature, which was not really the focus of the workflow.

**Minor comments:**

 Several sections should be edited for clarity and flow of ideas. Specifically,

- page 6: "We recommend scaledTPM for differential transcript usage so that the estimated proportions fit by DRIMSeq in the following sections correspond to the proportions of underlying abundance." Could the authors please rewrite/break up this sentence to improve readability?
- page 6, section 'Import counts into R/Bioconductor': the authors should clarify whether the referenced R package is for demonstration purposes only (i.e. should the user install the rnaseqDTU to perform any of the workflow?).
- page 6: could the concept of using counts from abundance be introduced/explained before referring to specific package parameters and settings?
- page 6: "The following code chunk is not evaluated, but instead we will load a pre-constructed matrix of counts". Could the authors please clarify this sentence? We assume this means that instead of constructing a matrix of counts (as in a typical workflow), pre-constructed data is loaded.
- page 7 "We ran the following unevaluated code chunks": does 'unevaluated' refer to not run in a typical workflow?
- page 7, 'Statistic analysis of differential transcript usage', second paragraph: could the description of txdf be moved to the previous section where it is constructed? This would help improve the flow.
- page 12: "(2) contain a transcript with a transcript adjusted p-value less than 0.05 which does not participate in DTU, so contain a falsely confirmed transcript": could the authors please rewrite this sentence for clarity.
- page 13: sentence "The testing of “this” vs “others”..." could be improved for clarity, e.g.: "DEXseq in its original version requires fitting of coefficients for each exon within a gene. Running DEXseq at a transcript-level considerably improves performance as fewer features per gene require fitting of coefficients."
- page 14, after the line "dxr <- as.data.frame(dxr[,columns]": showing head(dxr) could help in clarifying the output.
- page 15, in the code "paste0("suppa/group1.tpm")": the paste function is not necessary here.
- Section 'Evaluation of methods for DTU': could the authors offer an explanation why SUPPA2 only reported one DGE gene as DTU?
- Could the y and x axes on the plots on pages 17-20 and 25 be made consistent with each other? Also, very minor point, but these plots have some jpeg artefact. Could pdf or png plots be used instead?
- page 19 "DRIMSeq [...] performed slightly better": could a metric be referenced in how the package performed better?
- page 22: "We can repeat the same analysis...": 'same analysis' is misleading as this section tests only DGE.
- page 24: could the authors formally introduce or describe EBSeq and SAMseq packages, preferably earlier in the manuscript?
- page 26: could the authors use 'compute time' instead of 'timing'?

We identified the following typographical errors and grammatical issues:

- page 5: "We recommend [constructing] a CSV file..."
- page 6: "We suggest for DTU analysis to generate counts from abundance..." reword to "For DTU analysis, we suggest generating counts from abundance..."
- page 16: "DEXSeq controlled [the FDR] except for..."
- page 16: "DRIMSeq had [an] observed FDR.."
- page 16: "...reported 2 extra genes more than..." change to "reported two more genes than"
- page 16: "...DEXseq were the most sensitive methods [for] recovering"
- page 19 "...DRIMSeq and DEXSeq[,] [in] this additional simulation"
- page 19: "Again, we caveat our comparative evaluation of DRIMSeq and DEXSeq by noting that we do not know..." change to "Again, a caveat of our comparative evaluation of DRIMSeq and DEXSeq is that we do not know..."
- page 24: "did not have [adequate] sensitivity to detect DGE"
- page 24: "while those two method[s] had"

We have read this submission. We believe that we have an appropriate level of expertise to confirm that it is of an acceptable scientific standard, however we have significant reservations, as outlined above.

# Response

**Published:** 2018-09-11

**Authors:** Michael Love

**Affiliations:** University of North Carolina at Chapel Hill, USA

We thank all reviewers for their insightful comments and suggestions that we feel have greatly improved the readability and usefulness of the workflow. We summarize the main changes and then address reviewer-specific comments point-by-point:

- We have addressed all minor text or grammatical suggestions by the reviewers.
- We have re-organized the article into distinct and more separated Workflow and Evaluation sections, which was suggested by all reviewers. We begin the article with a clear outline, titled: "Structure of this article", which outlines the Workflow part and the Evaluation part. This outline has direct links to relevant sections and subsections which follow. We have also included an overview diagram of the methods and packages included in the Workflow section, and how they are interconnected.
- We have added to the Introduction more motivational text on why a DTU analysis is relevant for biology and biomedical research.
- We have added a large section describing the methods DEXSeq and DRIMSeq, before the Workflow section.
- We have expanded the original sections discussing counts-from-abundance and their use in the workflow, to make our use of the tximport method more clear.
- For the DEXSeq section, we have corrected an earlier incorrect use of nbinomLRT(), which is now replaced with the correct testForDEU(). The practical result is that DEXSeq performs somewhat less conservatively, but the original code was incorrect, and the fix is necessary. The incorrect use of nbinomLRT() in this context will now produce an error in future releases of Bioconductor, to avoid possible incorrect usage.
- We have added RATs to the DTU Evaluation.
- We now apply stageR to all DTU methods that are evaluated: DRIMSeq, DEXSeq, RATs, and SUPPA2. The RATs and SUPPA2 methods are described, but the code is not provided, as these packages are not part of the Workflow.
- We use consistent x-axes and y-axes whenever possible, and use PDF instead of JPG to reduce compression artifacts. When a consistent x-axis is not used in the main text, we include Supplementary Figures with the same plots with outlying methods dropped to keep the x-axis consistent.
- We use a palette in which colors are more discernable for color-blind readers
- In the Evaluation sections, we include additional plots which examine the simulated gene type source of false positives for the DTU, DGE, and DTE analyses.
- We added a new evaluation to examine performance differences between DRIMSeq and DEXSeq, using the identical simulated data that was used in Soneson et al (2016) and Nowicka and Robinson (2016).
- We have added a 2 vs 2 simulation for the DTU Evaluation.
- We added a brief overview description of all methods assessed in the DGE and DTE Evaluations.
- We have added more recommendations in the Discussion.

Reviewer-specific comments:

- We have tried to separate and clarify the Workflow section and the Evaluation section. We now include an overview diagram, as helpfully suggested here.
- We have expanded the section on counts-from-abundance, added a section before the counts are imported, and clarified the sentences highlighted by the reviewers.
- We have clarified a number of the "not evaluated" sentences in the original workflow.
- The description of txdf is given in the section where it is constructed, under the heading "Transcript-to-gene mapping".
- We have clarified the OFDR description in the sentence highlighted by the reviewers, and have removed the "this" vs "other" sentence, as the history of DEXSeq method development is not necessary or useful for the readers of this workflow.
- We have added `head(dxr)` to demonstrate the output.
- We have removed the SUPPA2 code, as now the workflow focuses on the Bioconductor package DRIMSeq and DEXSeq, which have live code examples (SUPPA2 is a python package and so cannot have live code examples in a Bioconductor workflow).
- We have made the x- and y-axes consistent whenever possible.
- We have revised the Workflow and Evaluation sections following all of the reviewers' helpful comments, error spotting, and suggestions on improved wording.

# Peer review: Referee response for version 1

**DOI:** 10.5256/f1000research.16780.r35548

**Published:** 2018-07-24

**Authors:** Kristoffer Vitting-Seerup, Malte Thodberg

**Affiliations:** 1 Department of Biology, Biotech Research and Innovation Centre, University of Copenhagen, Copenhagen, Denmark

**Summary**

 In “Swimming downstream: statistical analysis of differential transcript usage following Salmon quantification” Love *et al* presents a combined workflow and benchmark for differential transcript usage. This is a vital paper as there is no consensus on which differential transcript usage tools works better (here addressed by the benchmark part) and very few people analyze differential transcript usage – something the workflow can hopefully help with. Of special note is the extent to which open source have been embraced by Love *et al* – an approach that is commendable (and copy worthy). Although the manuscript has a lot of potential it can, in its current form, be challenging to read and the benchmark of differential transcript usage part needs to be extended. Revisions are therefore required.

**Preface**

- Malte Thodeberg helped me review this paper – thanks Malte!
- Since neither of us are native English speakers/writers we have not attempted to corrected for potential gramma and/or spelling mistakes
- I'm the developer of IsoformSwitchAnalyzeR.

**General comments**

- The article switches between describing a workflow, which users can follow to perform differential transcript usage on their own data, and a benchmark of differential expression/usage tools. The two sections should be much more clearly separated and each should be more concisely written.
  - One solution would be to have the benchmark first and the workflow afterwards. It would then be natural that workflow used the tool(s) deemed better by the benchmark.
- The main problem with the workflow part of the manuscript is the intermixing of the workflow and benchmarking (and the intro/methods) sections which makes it necessary to include a lot of callouts, omissions and special cases. This has the unintended effect of cluttered the workflow making it hard to read and/or follow. This would however be solved by the above suggested re-structuring. If such restructure were implemented it would also seem more natural that the workflow consistently only use a small dataset (either a subset of the simulated data or another dataset entirely) whereby the workflow could be simplified a lot.
- Although the benchmark is of high quality it still needs to be a bit more exhaustive.
- (Even with the suggested re-structure) The whole article would highly benefit from an overview paragraph and/or figure to give the reader the high-level overview of the outline before jumping into it (something like a table/figure/description of content). This could also be a table of content (with links included to enable easy jumping in the article).

**Title**

- The title should reflect it is a workflow and/or benchmark. The current title suggests the authors developed a new tool for differential transcript usage which were specifically designed to integrate with Salmon. Furthermore, it could be considered to change the title so it also indicates the differential gene/transcript expression performed in the manuscript.

**Introduction**

- The introduction lacks a section describing why differential transcript usage are of interest in the first place.
- Large parts of what would normally be in the introduction and methods have been moved into the results. Introduction to tools and methods including descriptions of how they work belongs in the introduction. Description of parameter choice for e.g. scaling during tximport also belongs in intro/methods.
  - Optional suggestion: include a lay-man introduction to how the tools work (the technical part are in the original papers for people interested).
- In the section where tools for DTU are mention please remove (or argue for inclusion of) BITSeq and stageR. StageR is for post analysis of p-values (no test). Although BITSeq is mentioned in some of the BiocViews of alternative splicing neither the article nor the vignette shows anything but DTE (aka no DTU). Mention that SGSseq wraps DEXSeq.
- The test build into IsoformSwitchAnalyzeR in not rank-based – but it is obsolete and will be removed from the next update – so it could be skipped entirely (along with the other non-maintained tests).
- Please reference IsoformSwitchAnalyzeR for its main purpose: the downstream analysis of functional consequences of identified isoform switches. Consider also mentioning other tools for downstream analysis (some can be found at https://www.bioconductor.org/packages/devel/BiocViews.html#___AlternativeSplicing ).
- To be more user-friendly please insert a link when mentioning the IsoformSwitchAnalyzeR vignette.

 

**Methods**

- Please add in the number of transcripts considered expressed (>= 10 estimated fragment counts)
- The simulations performed should either be named or numbered to allow for clear reference to which of the simulated datasets are used.
- In the countSimRepport please compare the simulated data to the 12 samples which were used for the basis of the simulation (comparing 12 to hundreds of samples is not easy to interpret).
- Please elaborate on discussion of the different options for scaling-from-TPM-to-counts. It is unclear what the difference is and when it matters. Furthermore you write “if we used lengthScaledTPM transcript counts, then a change in transcript usage among transcripts of different length could result in a changed total count for the gene, even if there is no change in total gene expression” is there a mixup here? If not, why do you then use lengthScaledTPM in the DGE/DTU section? Please include a recommendation of when to use which option for analysis of DGE/DTE, DTU and if both are present in the data.
- Modifications
  - Include a paragraph on quantification before introducing the modifications. If any expression filtering was done (as fig 1 indicate and mention above) it should be clearly stated.
  - Currently it is unclear how many genes were modified in which way. To remedy that please provide a table indicating the number genes modified for DTU or DGE by each of the changes you introduce (as well as the total number of genes modified.
  - Why both simulate DTU with a modification of a single isoform and a switch of two isoforms if you are not investigating whether it makes a difference - seems redundant? (more on that in the DGE benchmark).

 

**In the workflow**

- Please add a comment of why DRIMSeq have NA as p-values (that will confuse many people)

 

**Post-hoc filtering on DRIMSeq**

- What is the reasoning beheading this filtering step? And is it statistically valid to do this filtering – the proportions and p-values are not independent. Is the modified p-value distribution still uniform in the interval [0.05-1[ enabling proper FDR correction?
- If the filtering is statistically sound why not also do it for the other methods?

 

**Evaluation of methods for DTU**. This is the major selling point of the article and the part that require most work.

- To reflect a very common-use case scenarios the benchmark should also be formed with 2 replicates. Since the benchmark presented here show quite subtle differences (in TPR vs FDR) between 9 and 12 replicates the 2-replicate scenario could for replace either of them.
- The benchmark simulation should not only be performed once (one time) as the exact samples used in that run will have a large effect (especially for the smaller comparisons). Instead 25 simulations should be performed and the average iCOBRA plot could be shown (possibly extended to also show variation across the simulations).
- The benchmark must also include a run on unmodified simulated data to test how many false positives are found if there truly are no DTU (which might be the case for some datasets).
- Be consistent and concise in the use of stageR. Either use with no tools or use with all tools (or both to also enable a benchmark of stageR). Else the transcript level FDR between tools are not comparable). Highlight the difference between perGeneQValue and stageR (or only use one of them) or highlight where each is used. For example, it is not clear whether stageR was used in figure 3 and if it was whether it was for all tools.
- Given the success of repurposing DEXSeq to DTU, and the good performance of limma for DTE/DGE, the current benchmark could also test a repurposing of limma’s (and edgeR’s) differential exon usage test. This is optional – but it would be a huge step forward for testing differential isoform usage as it would bring a lot of clarity to the field.
- Use same axis for the 4 iCOBRA plots to illustrate improvement with increasing number of samples. Please include group sizes (e.g. 3 vs 3, 6 vs 6 etc.) in the figure to make it easier to read - could be instead of the rather uninformative “overall” facet title.
- Please comment:
  - On the large performance increase from “Kallisto + DEXSeq” in Soneson el al, Genome Biology 2016 (where FDR performance was quite poor) to the current “Salmon + DEXSeq” which performs rather good.
  - On the differences between your benchmark (indicating DEXSeq works better) and the benchmark performed by Nowicka et al in the DRIMSeq paper (indicating DRIMSeq) works better.
- Please move the evaluation with fixed per-gene dispersion to supplementary material as it is just a sanity check.
- Please end section with a recommendation of what tool to use.

 

**Evaluation of DTU vs DGE**

- This section belongs in the workflow part of the article.

**Evaluation of DGE/DTE**

- The reason for (re)doing a DGE/DTU benchmark here need to be clearly described (which is to test how tools perform when there are also underlying DTU as hinted in Soneson 2016, F1000Research).
- To reflect a very common-use case scenarios the benchmark should also be formed with 2 replicates. The 2-replicate scenario could replace either the 9 or 12 replicates
- Table with runtime should be moved to supplementary as it can be summarized as “sleuth is slower”.
- The TPR vs FDR figures are unreadable due to too many lines on top of one another – this must be fixed. Furthermore, use same axis for the 4 iCOBRA plots to show improvement with increasing number of samples. Please include group sizes in the figure to make it easier to read - could be instead of the “overall” facet title.
- The DGE results are quite surprising – in other recent benchmarks most tools handle FDR quite well – which is not the case here.
  - I suspect this might be due to the DGE where only a single isoform was changed (meaning the overall gene expression could change only marginally). Therefore, the authors should investigate how the benchmark result differ when only considering either the DGE introduce with one isoform upregulated or the DGE with all isoforms were upregulated.
  - If the results hold op a comment on how this compare to recent DGE benchmarks is necessary
- If the problem rather seems to be the presence of DTU this should be highlighted and discussed.
- For figure S2 please include the sleuth result on the main simulated data as well else a direct comparison (to judge the effect of the GC content) is not feasible
- Please end section with a recommendation of what tools to use.

 

**Discussion**

- There also needs to be a discussion around the benchmark part of the paper – it is currently completely missing.

Please don't hesitate to contact me if anything was unclear.

We have read this submission. We believe that we have an appropriate level of expertise to confirm that it is of an acceptable scientific standard, however we have significant reservations, as outlined above.

# Response

**Published:** 2018-09-11

**Authors:** Michael Love

**Affiliations:** University of North Carolina at Chapel Hill, USA

We thank all reviewers for their insightful comments and suggestions that we feel have greatly improved the readability and usefulness of the workflow. We summarize the main changes and then address reviewer-specific comments point-by-point:

- We have addressed all minor text or grammatical suggestions by the reviewers.
- We have re-organized the article into distinct and more separated Workflow and Evaluation sections, which was suggested by all reviewers. We begin the article with a clear outline, titled: "Structure of this article", which outlines the Workflow part and the Evaluation part. This outline has direct links to relevant sections and subsections which follow. We have also included an overview diagram of the methods and packages included in the Workflow section, and how they are interconnected.
- We have added to the Introduction more motivational text on why a DTU analysis is relevant for biology and biomedical research.
- We have added a large section describing the methods DEXSeq and DRIMSeq, before the Workflow section.
- We have expanded the original sections discussing counts-from-abundance and their use in the workflow, to make our use of the tximport method more clear.
- For the DEXSeq section, we have corrected an earlier incorrect use of nbinomLRT(), which is now replaced with the correct testForDEU(). The practical result is that DEXSeq performs somewhat less conservatively, but the original code was incorrect, and the fix is necessary. The incorrect use of nbinomLRT() in this context will now produce an error in future releases of Bioconductor, to avoid possible incorrect usage.
- We have added RATs to the DTU Evaluation.
- We now apply stageR to all DTU methods that are evaluated: DRIMSeq, DEXSeq, RATs, and SUPPA2. The RATs and SUPPA2 methods are described, but the code is not provided, as these packages are not part of the Workflow.
- We use consistent x-axes and y-axes whenever possible, and use PDF instead of JPG to reduce compression artifacts. When a consistent x-axis is not used in the main text, we include Supplementary Figures with the same plots with outlying methods dropped to keep the x-axis consistent.
- We use a palette in which colors are more discernable for color-blind readers
- In the Evaluation sections, we include additional plots which examine the simulated gene type source of false positives for the DTU, DGE, and DTE analyses.
- We added a new evaluation to examine performance differences between DRIMSeq and DEXSeq, using the identical simulated data that was used in Soneson et al (2016) and Nowicka and Robinson (2016).
- We have added a 2 vs 2 simulation for the DTU Evaluation.
- We added a brief overview description of all methods assessed in the DGE and DTE Evaluations.
- We have added more recommendations in the Discussion.

Reviewer-specific comments:

General comments

 We believe we have made the separation between Workflow and Evaluation much more clear now, and have added an outline to the beginning of the article with hyperlinks to subsections and with an overview diagram, as usefully suggested here.

Title

 We believe the title is appropriate and does not suggest a new tool. The fact that existing tools are leveraged in the workflow is clear from the abstract and the main text.

Introduction

 The Bioconductor workflows do not have typical structure with Introduction, Methods, Results and Discussion, but instead a prolonged section where relevant concepts are typically introduced as needed. See, for example, the DESeq2 workflow: https://bioconductor.org/packages/rnaseqGene. We have now added overview descriptions of the methods DEXSeq and DRIMSeq before the Workflow section begins.

 We have removed BitSeq. We believed earlier that cjBitSeq, which is a new DTU method, was implemented in the Bioconductor package BitSeq, but it is a separate GitHub package (https://github.com/mqbssppe/cjBitSeq). Since we are listing Bioconductor packages that can be used for DTU, we now do not list BitSeq. We now have a separate sentence describing stageR and its connection to the DTU methods, and SGSeq (and we mention its leveraging of DEXSeq or limma). 

 We no longer mention the statistical test from Vitting-Seerup and Sandelin (2017). We use the suggested purpose description for IsoformSwitchAnalyzeR, link to the AlternativeSplicing BiocViews, and include a link to the IsoformSwitchAnalyzeR vignette.

Methods

 We now include the number of transcripts with estimated counts greater than 10 in the Simulation. We name the various simulations, and use their name when referring to them in the main text or captions.

 Our purpose in using the countsimQC report is to compare the joint distribution of estimated  parameters (mean, dispersion) from the simulation and from the dataset from which the estimates were derived. We therefore compare the 24 simulated samples to the 458 non-duplicated GEUVADIS samples that were used for the estimation of the mean and dispersion parameters. We have made this more clear in the caption of the countsimQC Supplementary Figure.

 We have elaborated on discussion of the different options for counts-from-abundance, including the sentence about change in total counts. We include details on the recommended counts-from-abundance options through the text and in the overview diagram, Figure 1.

 We state whenever any expression filtering was done. The only expression filtering in the DTU section is performed by the filtering functions in DRIMSeq, and the TPM > 1 filter to speed up SUPPA2 on the command line. We mention the various expression filters used by the different DGE and DTE methods in the Evaluation section for those methods. We include in the Simulation section the exact number of genes modified by simulated DGE, simulated DTE, and simulated DTU.

 We have added a comment on the NA p-values for DRIMSeq in the section in the workflow where they are replaced with a p-value of 1. The text now reads:

 "From investigating these NA p-value cases for DRIMSeq, they all occur when one condition group has all zero counts for a transcript, but sufficient counts from the other condition group, and sufficient counts for the gene. DRIMSeq will not estimate a precision for such a gene. These all happen to be true positive genes for DTU in the simulation, where the isoform switch is total or nearly total. DEXSeq, shown in a later section, does not produce NA p-values for any genes. A potential fix would be to use a plug-in common or trended precision for such genes, but this is not implemented in the current version of DRIMSeq."

 We now perform post-hoc proportion SD filtering on the adjusted transcript p-values for DRIMSeq directly, which has little effect on the results. The SD of proportions and the p-values may possibly be independent under the null hypothesis of no DTU, which is the requirement for proper Type I error control of an independent filter [Bourgon (2010)], but we do not attempt to provide empirical evidence to support this. Importantly, we apply the post-hoc filtering because we have empirical evidence that DRIMSeq was not providing uniform p-values for null transcripts on the simulated data explored in this article. Therefore, we begin with a non-uniform distribution of p-values for the null transcripts. The filtering is shown empirically to improve the FDR control.

 We do not perform the simulation multiple times, and we have not extended iCOBRA to support multiple iterations on a single plot, which is beyond the scope of this article. We are most interested in the relative performance of the various methods, and their general location on the TPR-FDR plots, which is achieved with the current evaluation. We did explore running DEXSeq 25 times on the 3 vs 3 "main" simulation, and the inter-simulation variation in the TPR-FDR plot was minimal. We have uploaded all 24 of the simulated paired-end reads to Zenodo, and the dataset is already quite large. We do not run the methods on entirely null datasets, which is beyond the scope of this article.

 We have now used stageR on all methods. stageR accepts gene-level p-values (or adjusted p-values) and transcript-level p-values. If gene-level p-values are not provided by a method then DEXSeq's perGeneQValue was used to generate gene-level adjusted p-values, for use with stageR.

 We do not evaluate other methods for exon usage, as we focus in the workflow on Bioconductor methods that have been already proposed and evaluated for DTU analysis in publications.

 We now use consistent axes, and include the group size in the strip titles.

 We now evaluate DRIMSeq and DEXSeq on the identical simulation dataset used in both Soneson et al (2016) and Nowicka and Robinson (2016). We find similar performance of DEXSeq as reported in those papers using a less stringent transcript filter, but when we use DRIMSeq count and proportion filters as recommended in this workflow, the performance of DEXSeq is greatly improved, to levels consistent with what we see in the "main" simulation.

Evaluation of DGE/DTE

 We clarify why a DGE and DTE evaluation is included.

 We do not perform a 2 replicate DGE or DTE evaluation, as this is beyond the scope of the article.

 We now breakdown the DGE and DTE results by simulated gene type. We do not see any strong enrichment of one simulated gene type in the false positive breakdown plots. We believe our evaluation may differ from others in exploring the consistency of results as sample size increases.

Discussion

 We now include in the Discussion some recommendations on tool usage and performance.
